# Molecular Epidemiology of Salmonellosis in Florida, USA, 2017–2018

**DOI:** 10.3389/fmed.2021.656827

**Published:** 2021-04-22

**Authors:** Nitya Singh, Xiaolong Li, Elizabeth Beshearse, Jason L. Blanton, Jamie DeMent, Arie H. Havelaar

**Affiliations:** ^1^Animal Sciences Department, Emerging Pathogens Institute, Food Systems Institute, University of Florida, Gainesville, FL, United States; ^2^Department of Environmental and Global Health, Emerging Pathogens Institute, University of Florida, Gainesville, FL, United States; ^3^Bureau of Public Health Laboratories, Florida Department of Health, Jacksonville, FL, United States; ^4^Independent Researcher, Orlando, FL, United States; ^5^Food and Waterborne Disease Program, Florida Department of Health, Tallahassee, FL, United States

**Keywords:** hierarchical clustering, outbreak detection, phylogeny, SNP, cgMLST, mlst, whole genome sequencing, *Salmonella enterica*

## Abstract

The state of Florida reports a high burden of non-typhoidal *Salmonella enterica* with approximately two times higher than the national incidence. We retrospectively analyzed the population structure and molecular epidemiology of 1,709 clinical isolates from 2017 and 2018. We found 115 different serotypes. Rarefaction suggested that the serotype richness did not differ between children under 2 years of age and older children and adults and, there are ~22 well-characterized dominant serotypes. There were distinct differences in dominant serotypes between Florida and the USA as a whole, even though *S*. Enteritidis and *S*. Newport were the dominant serotypes in Florida and nationally. *S*. Javiana, *S*. Sandiego, and *S*. IV 50:z4, z23:- occurred more frequently in Florida than nationally. Legacy Multi Locus Sequence Typing (MLST) was of limited use for differentiating clinical *Salmonella* isolates beyond the serotype level. We utilized core genome MLST (cgMLST) hierarchical clusters (HC) to identify potential outbreaks and compared them to outbreaks detected by Pulse Field Gel Electrophoresis (PFGE) surveillance for five dominant serotypes (Enteritidis, Newport, Javiana, Typhimurium, and Bareilly). Single nucleotide polymorphism (SNP) phylogenetic-analysis of cgMLST HC at allelic distance 5 or less (HC5) corroborated PFGE detected outbreaks and generated well-segregated SNP distance-based clades for all studied serotypes. We propose “combination approach” comprising “*HC5 clustering,”* as efficient tool to trigger *Salmonella* outbreak investigations, and “*SNP-based analysis,”* for higher resolution phylogeny to confirm an outbreak. We also applied this approach to identify case clusters, more distant in time and place than traditional outbreaks but may have been infected from a common source, comparing 176 Florida clinical isolates and 1,341 non-clinical isolates across USA, of most prevalent serotype Enteritidis collected during 2017–2018. Several clusters of closely related isolates (0–4 SNP apart) within HC5 clusters were detected and some included isolates from poultry from different states in the US, spanning time periods over 1 year. Two SNP-clusters within the same HC5 cluster included isolates with the same multidrug-resistant profile from both humans and poultry, supporting the epidemiological link. These clusters likely reflect the vertical transmission of *Salmonella* clones from higher levels in the breeding pyramid to production flocks.

## Introduction

Nontyphoidal *Salmonella enterica* (hereafter referred to as *Salmonella*) is a significant public health problem and one of the most frequent cause of bacterial gastroenteritis worldwide (1, 2). *Salmonella* is a leading cause of foodborne illness in the United States, causing about 1.2 million annual cases annually (3). The state of Florida has consistently observed incidence rates of salmonellosis that are approximately twice as high as the national average (4) Annually, 6,000–7,000 cases of salmonellosis are confirmed in the state, causing a high burden on surveillance systems including detection of outbreaks and implementation of control measures.

Several well-established and standardized methods have been commonly used for the subtyping of *Salmonella*, including sero- and phage typing, pulsed-field gel electrophoresis (PFGE), multi-locus variable numbers tandem repeat analysis (MLVA), and multi-locus sequence typing (MLST). PFGE has been the gold standard method for the subtyping of *Salmonella* (5), and gained widespread adoption under PulseNet USA, a surveillance network of public health laboratories across the United States and other countries (6). However, the limited resolution of PFGE for closely related strains, specifically those involved in outbreaks has impeded its utility as an effective subtyping tool. For example, for *S. enterica* serotype Enteritidis, reported as one of the most prevalent serotypes among laboratory confirmed cases (7), a considerable number of isolates (45%) received by PulseNet USA belonged to indistinguishable PFGE patterns using the XbaI restriction enzyme (8), whereas, 74% of *S*. Enteritidis isolates were found to comprise of only three unique PFGE patterns in another study (9). Although, additional restriction enzymes could be used to increase the discriminatory power of PFGE (10), the laboratory protocol would also become more time consuming and expensive. Similarly, despite offering better discrimination, MLVA has had limited use because of its inability to differentiate common patterns (11), and standardized PulseNet protocols have been established for serotypes Enteritidis and Typhimurium only. Legacy (7-gene based) MLST has been a valuable tool for inferring evolutionary descent and clonal diversification among genetic lineages within bacterial population structures (12, 13). However, the use of only seven housekeeping genes fails to capture genetic heterogeneity within strains of the same serotype and suffers from lower discriminatory power (14).

In recent years, decreasing costs of sequencing technologies and advances in bioinformatics tools have greatly facilitated the development of whole-genome sequencing (WGS) methods that have been transformational for the surveillance of foodborne pathogens in the public health infrastructure (15). With the ultimate subtyping resolution delivered by interrogating entire genomes, an expansion of studies have demonstrated the utility of WGS to enhance our capacity to detect outbreaks (16–18), conduct surveillance (19) and understand the genomic population structure of *Salmonella* (8, 14).

Core genome MLST (cgMLST) offers a reliably fast method for identifying closely related strains within the same serotypes and is used e.g., by the Centers for Disease Control and Prevention (CDC) for outbreak detection (20). Precompiled assemblies and genotyped strains for all *Salmonella* WGS reads submitted to NCBI are available at Enterobase (http://enterobase.warwick.ac.uk/), an online resource for analyzing and visualizing genomic variation within enteric bacteria (14, 21). This offers an attractive solution to identify closely related genetic isolates, which can be further evaluated as potential outbreaks via more extensive SNP-based protocols. WGS data can also be highly valuable in source identification of clinical cases through comparison with isolates from food, animals and other non-clinical sources which are made available to NCBI mainly through GenomeTrakr (22).

The Florida Department of Health's Bureau of Public Health Laboratories (BPHL) started to implement WGS in 2016, with full coverage since 2019 and employed PFGE-based subtyping for molecular surveillance through 2018 in collaboration with CDC within PulseNet USA. In 2019, PulseNet transitioned to WGS-based methods and BPHL also ceased the use of PFGE for *Salmonella* isolate typing. WGS data are routinely shared with CDC, who analyze the data for multistate outbreaks, while detecting outbreaks restricted to the state of Florida is the responsibility of the Florida Department of Health (FDOH). An efficient tool for analyzing the high volume of sequence data is needed for this purpose. Phylogenetic methods, while highly specific, are using computationally intensive and time-consuming methods and thus present a challenge for efficient outbreak detection in the state. In the current work we are proposing a two-step “combination approach” (cgMLST-SNP phylogeny) which can be applied for different purposes like outbreak detection and identification of case- clusters and putative sources. As a first step, cgMLST can be used to narrow down the number of genetically related isolates at allelic level and in a second step, SNP-based phylogeny can identify genetic relatedness of isolates at a more detailed level.

Against this background, the research objectives of this study were to:

1. Understand the population structure of human *Salmonella* isolates in Florida, using molecular serotyping and multi-locus sequence typing (7-gene (legacy) MLST, core genome (cg) MLST);
2. Evaluate the potential of whole genome sequencing data analysis to improve detection of foodborne disease outbreaks for all serotypes of *Salmonella* while accounting for the large volume of isolates in Florida;
3. Understand the genetic relationship between clinical isolates from putative outbreaks and non-clinical *Salmonella* isolates from Florida and elsewhere in the United States collected during 2017–2018.

## Materials and Methods

### Data

Whole genome sequencing data used for the current work were downloaded from public repositories (NCBI and Enterobase), while metadata used for data collection and management were collected from FDOH and the US Department of Agriculture Food Safety and Inspection Service (USDA FSIS).

#### WGS Data Generation

BPHL in Jacksonville, FL receives *Salmonella* isolates from all diagnostic laboratories in Florida under updated Rule 64D-3.029 and generates WGS data (since 2016) for surveillance purposes, following a standardized CDC sequencing protocol (23) as follows: isolates were grown on trypticase soy agar plates with 5% sheep blood (Remel, Lenexa, KS, USA). DNA extraction was conducted using the Qiagen DNeasy Blood & Tissue Kit (Qiagen, Hilden, Germany). DNA libraries were constructed using Nextera XT DNA Library Prep Kit (Illumina, Inc., San Diego, CA, USA). The genomic libraries were sequenced on the Illumina MiSeq system using a combination of v2 (500 cycle) and v3 (600 cycle) chemistries. BPHL stores all sequence and related metadata in a Bionumerics database. All sequenced data are shared with CDC for surveillance purposes. These data are submitted to the NCBI BioSample database (https://www.ncbi.nlm.nih.gov/biosample/), under Bioproject number PRJNA230403 with limited metadata information.

#### Metadata Collection and Management

An integrated and deidentified metadata set from BPHL Bionumerics and FDOH Merlin database was made available by FDOH under a data sharing agreement, details are provided in Li et al. (4). We selected isolates from metadata received from FDOH, with a date of sample collection by the clinical laboratory from January 2017 to December 2018 and downloaded sequence data from Enterobase (https://enterobase.warwick.ac.uk) using PNUSA numbers specified in the metadata (https://dataverse.harvard.edu/dataverse/Salmonella_FL). After data cleaning [as detailed in Li et al. (4), involved removal of isolates with missing data or duplicated in the database] a curated set of 1,709 isolates was available for further analysis. We additionally included WGS data from Enterobase of 230 isolates collected from non-clinical sources (including soil/water, food, poultry, animal sources) in Florida and 1,317 *S*. Enteritidis isolates collected from poultry sources from other states during same time period, using identifiers from NCBI. We segregated clinical and non-clinical isolates into four different isolate sets, comprising:

1. “*all clinical,”* a set of all 1,709 clinical isolates for exploring outbreak detection by cgMLST clustering and SNP based phylogeny;
2. “*sporadic clinical,”* a set of 1,632 isolates including 1,595 sporadic cases and 37 first occurrences of isolated from outbreaks flagged by PFGE surveillance for exploring serotype, serotype diversity, and sequence type population structure;
3. “*sporadic clinical and non-clinical*,” a set of 1,862 isolates, obtained by adding non-clinical isolates (230) to the sporadic clinical isolates, for exploring any connection between clinical and non-clinical isolates;
4. “*Enteritidis poultry,”* a set of 1,341 isolates of *S*. Enteritidis collected during 2017–2018, from poultry sources across the USA (including 24 Florida isolates), for exploring possible genetic links between clinical isolates of *S*. Enteritidis in Florida and poultry isolates across USA.

Additional metadata (day, month, year of collection) for all non-clinical isolates were provided by the US Department of Agriculture Food Safety and Inspection Service through a Freedom of Information Act request.

All datasets were cleaned, organized, and visualized in the statistical language R version 3.6.0.

### Serotype Diversity

All isolates were serotyped by employing the *in-silico* typing resource SISTR (https://lfz.corefacility.ca/sistr-app/), as implemented in Enterobase. We submitted ambiguous serotype assignments to the developers, who resolved them using SISTR ver. 2 (24).

Rarefaction analysis was performed on “*sporadic clinical”* isolates set, based on Hill numbers (^*q*^*D*) to represent serotype diversity (25) for q = 0, 1, and 2 using R package “*rareNMtests*.” Hill numbers are also referred as *order of diversity* and differ in sensitivity of diversity values to rare vs. abundant serotypes. The exponent q modulates the type of weighted mean and with *q* = 0 ~ weighted harmonic mean (serotype richness), *q* = 1 ~ geometric mean (exponential Shannon index representing “typical” serotypes) and *q* = 2 ~ arithmetic mean (inverse Simpson index representing effective number of “dominant” serotypes). Rarefaction was done for all cases and separately for cases <2 and ≥ 2 years of age.

A tanglegram was created to compare the rankings of top 20 serotypes in Florida and in the whole USA. The USA data was obtained from the National Enteric Disease Surveillance: Salmonella Annual Report, 2016 (26), which is the most recent available report.

### Multilocus Sequence Typing

#### MLST-Based Phylogeny

Enterobase implements both the traditional MLST approach based on sequence profiles of seven housekeeping genes (legacy MLST) and core genome MLST (cgMLST). Based on legacy MLST, eBurst groups (eBG) are defined as clusters of isolates sharing at least 6 common allelic sites. A minimum spanning tree (MSTree) based on legacy MLST was generated using the Enterobase MSTreeV2 algorithm and was visualized in GrapeTree (21) for the “*sporadic clinical”* isolate set with color-coding by eBG and labeled by SISTR predicted serotype. We also cross-tabulated the eBG groups associated with effective number of serotypes in the state as identified by rarefaction and corresponding legacy MLST Sequence Types.

Core genome MLST profiles were created using the Enterobase platform (14) for all four samples sets and MSTrees were created for first three isolates sets and visualized as above, while isolate set four was further filtered and used in SNP based analysis as described in sections Association of Clinical and Non-clinical Isolates and Florida Clusters. For tree construction, the distance between genomes was calculated using the number of shared cgMLST alleles and genomes were linked using single-linkage clustering. The MSTree for the “*sporadic clinical”* isolates was color-coded by SISTR predicted serotypes and the MSTree for “sporadic clinical + non-clinical” isolates set was color coded by source niche (clinical and non-clinical sources, *viz*., poultry, soil/water, raw chicken, avian, food etc.,) as available at NCBI. If undefined at NCBI, the source niche field for clinical isolates was updated as human based on the FDOH metadata. Hierarchical clusters (HC) of closely associated isolates having cgMLST allelic difference of ≤ 2, 5, 10, and 20) were generated and stable cluster group numbers referred as HC2, HC5, HC10, and HC20, respectively, were assigned to isolates in the “*all clinical”* set.

#### cgMLST Hierarchical Clustering for Outbreak Detection

We compared PFGE-based outbreak detection as implemented in Florida in 2017–2018 with the potential results that could have been obtained with cgMLST-based hierarchical clustering analysis. This comparison was restricted to the five most commonly detected serotypes in the “*all clinical”* set. HC5 clusters with >2 isolates were selected for further analysis and joined with all outbreak-associated isolates for selected serotypes, as detected by PFGE surveillance. The selected serotypes included: Enteritidis (100 isolates), Newport (50 isolates), Javiana (13 isolates), Bareilly (38 isolates), and Typhimurium including the monophasic variant I 4, [5],12:i:- (32 isolates).

### SNP-Based Phylogeny of cgMLST Hierarchical Clusters

SNP based phylogenies were constructed for all selected serotypes in the previous step. Core-genome single-nucleotide polymorphisms (core-SNPs) were identified using the Snippy pipeline v4.3.8 (https://github.com/tseemann/snippy). All isolates of selected serotypes belonged to same eBG type, justifying the choice of one reference genome except *S*. Newport for which we chose a reference genome close to the dominant eBG type in the sample set. Reference genomes for each selected serotype were *S*. Enteritidis (P125109, accession number: AM933172), *S*. Newport (AML0800465, accession number: AANRFQ010000001), *S*. Bareilly (CFSAN000189, accession number: CP006053), *S*. Javiana (CFSAN001992, accession number: NC_020307), *S*. Typhimurium ( LT2, accession number: NC_003197), and their respective annotated GenBank files were downloaded from NCBI. Assembled genomes for all serotypes were mapped against the corresponding reference genome using *BWA-mem* (27) and *minimap2* (28), and SNPs were called using *SAMtool*s (29) and *FreeBayes* (https://github.com/ekg/freebayes). SNPs located in repetitive and recombinogenic regions were removed prior to phylogenetic analysis using *Gubbins v2.3.4*. (30) Only parsimony informative polymorphic sites extracted from the *Gubbins* output were used for further analysis. SNP distance matrices were calculated using the *snp-dists* tool (https://github.com/tseemann/snp-dists) and imported in R along with metadata information about HC5 cluster labels, isolate dates, PFGE profile, and zip codes. Maximum Likelihood Trees with 1,000 bootstrap replications were generated using *iqTree V1.6.10* package with best fitting nucleotide evolutionary model identified by *ModelFinder* (31). Bootstrap supported trees were imported into R and were annotated/color-coded using metadata to reflect isolate date and HC5 cluster and were visualized using package *ggtree* (32). Further, for all major HC5 clusters SNP distance-based heatmaps and annotated trees were generated using *ComplexHeatmap* (33), *ape* (34), and *ggtree*. A detailed manual comparison was done between outbreak detection based on PFGE profiling and HC5 clusters, while taking into account phylogenetic relationships as defined by SNP analysis.

### Spatial Distribution of HC5 Clusters

Isolates assigned to HC5 clusters of size >10 in the “*all clinical”* set were plotted on a map of Florida at the zip-code level (only available for clinical isolates) to explore their spatial distribution. Standard distance was used to quantify the spatial distribution of zip-code areas involved in each HC5 cluster. Similar to the concept of standard deviation, standard distance is a metric in spatial statistics measuring the dispersion of features around their geographical mean center which is constructed from the mean x-coordinate and mean y-coordinate (35). The standard distance is given as:

(1) $$\begin{array}{l} \text{SD}=\;\sqrt{\frac{\overset{n}{\underset{i=1}{\sum }}{\left(x_{i}-\;\bar{X}\right)}^{2}}{\text{n}}+\frac{\overset{n}{\underset{i=1}{\sum }}{\left(y_{i}-\;\bar{Y}\right)}^{2}}{\text{n}}} \end{array}$$

where *x*_*i*_ and *y*_*i*_ are the coordinates of feature *i* and $\bar{X}$ and $\bar{Y}$ represent the mean center of features. Euclidean distances between each pair of centroids of zip-code areas within clusters were calculated, and the distance matrices were used to plot heatmaps for visualization.

### Association of Clinical and Non-clinical Isolates

MSTrees for the “*sporadic clinical and non-clinical”* isolates were constructed as described in section WGS Data Generation to aid the comparative visualization of possible shared sources of isolates. We compared HC5 cluster profiles of 1,862 Florida isolates to identify any matching non-clinical isolates in HC5 clusters of clinical isolates.

As a proof of principle, we applied the proposed combination approach for investigating genetic links between clinical and non-clinical isolates of *S*. Enteritidis (the most prevalent serotype in Florida and across the USA) (26). One seventy six clinical and 24 non-clinical *S*. Enteritidis isolates collected from Florida were first filtered for shared HC5 cluster profiles and were then further investigated by SNP based phylogenetic analysis, as above.

Guided by the results of this step, we additionally created HC5 cluster profiles of 1,317 poultry isolates of *S*. Enteritidis collected during 2017–2018 from other states and included them in above comparison to explore the infection sources across the US. Additionally, presence of antimicrobial resistance (AMR) genes was investigated using NCBI's AMRFinder (36), using online server (37), for clinical and poultry isolates that shared HC5 cluster profiles.

## Results

### Serotype Diversity

**Figure 1:**
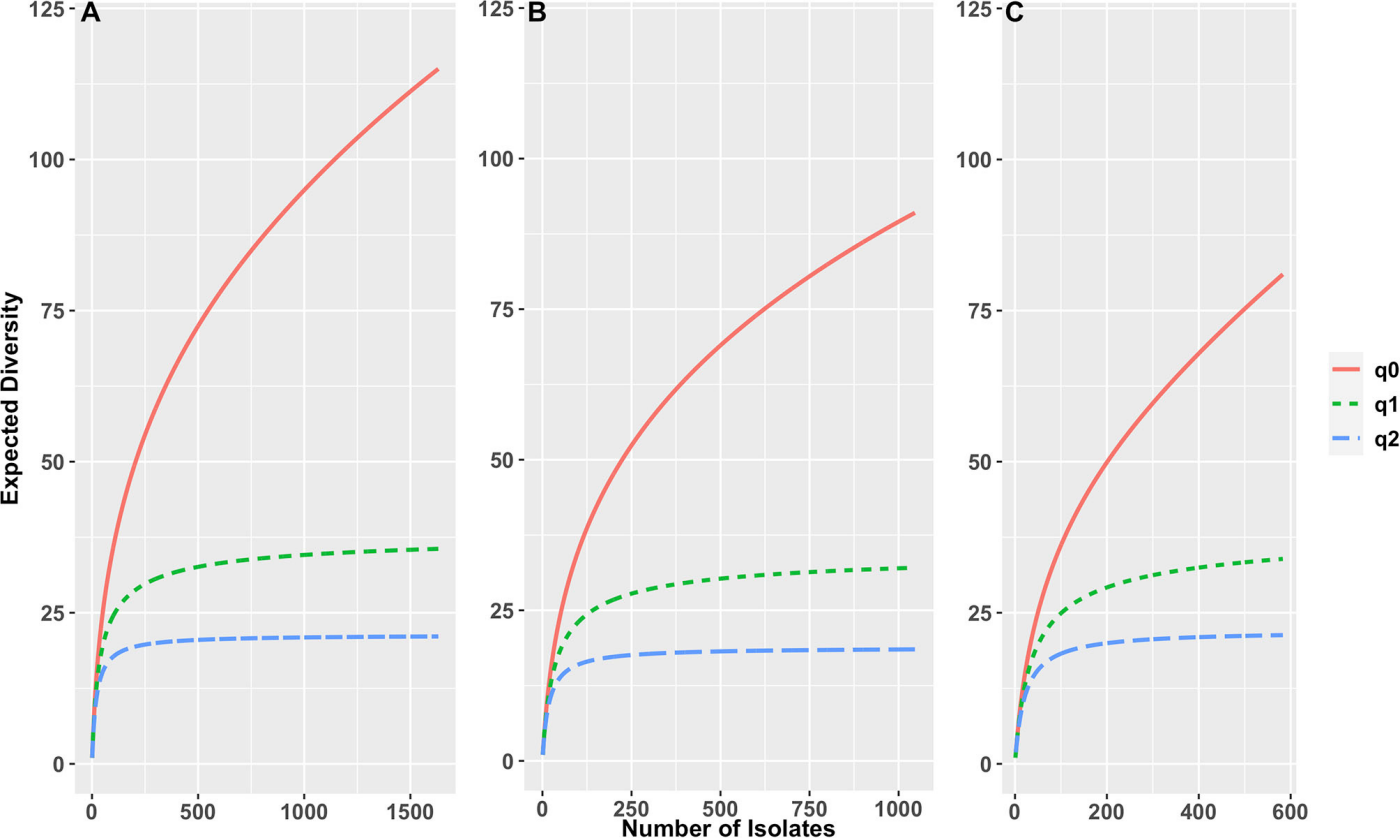
Rarefaction curves based on sample size-based diversity accumulation, for **(A)** 1,632 *S*. *enterica* isolates from all ages, **(B)** 1,155 isolates of age ≥ 2 years, **(C)** 477 isolates of age < 2 years, from Florida, USA 2017–2018. X-axis shows number of isolates, y axis shows expected diversity using three Hill numbers indicating the total number of serotypes in the set (q = 0), the number of “typical” serotypes (q = 1) and the number of “dominant” serotypes (q = 2).

The serotype diversity was calculated using the “*sporadic clinical”* set of 1,632 clinical isolates (Figure 1). There were 115 serotypes in the set (^0^*D*), the rarefaction curve suggests that significantly more serotypes are circulating in Florida as the Hill curve for *q* = 0 continued to increase with increasing number of isolates in the set. Many of the observed serotypes were rare, as demonstrated by the much lower number of ~36 typical serotypes (*q* = *1*), while there were ~22 dominant serotypes (*q* = *2*). Among 1,155 cases of age ≥ 2 years, there were 95 serotypes (*q* = 0), ~32 typical serotypes (*q* = *1*) and ~18 dominant serotypes (*q* = *2*), while for 477 cases of age <2 years, there were 76 serotypes (*q* = 0), with ~32 typical serotypes (*q* = *1*) and ~20 dominant serotypes (*q* = *2*).

**Figure 2:**
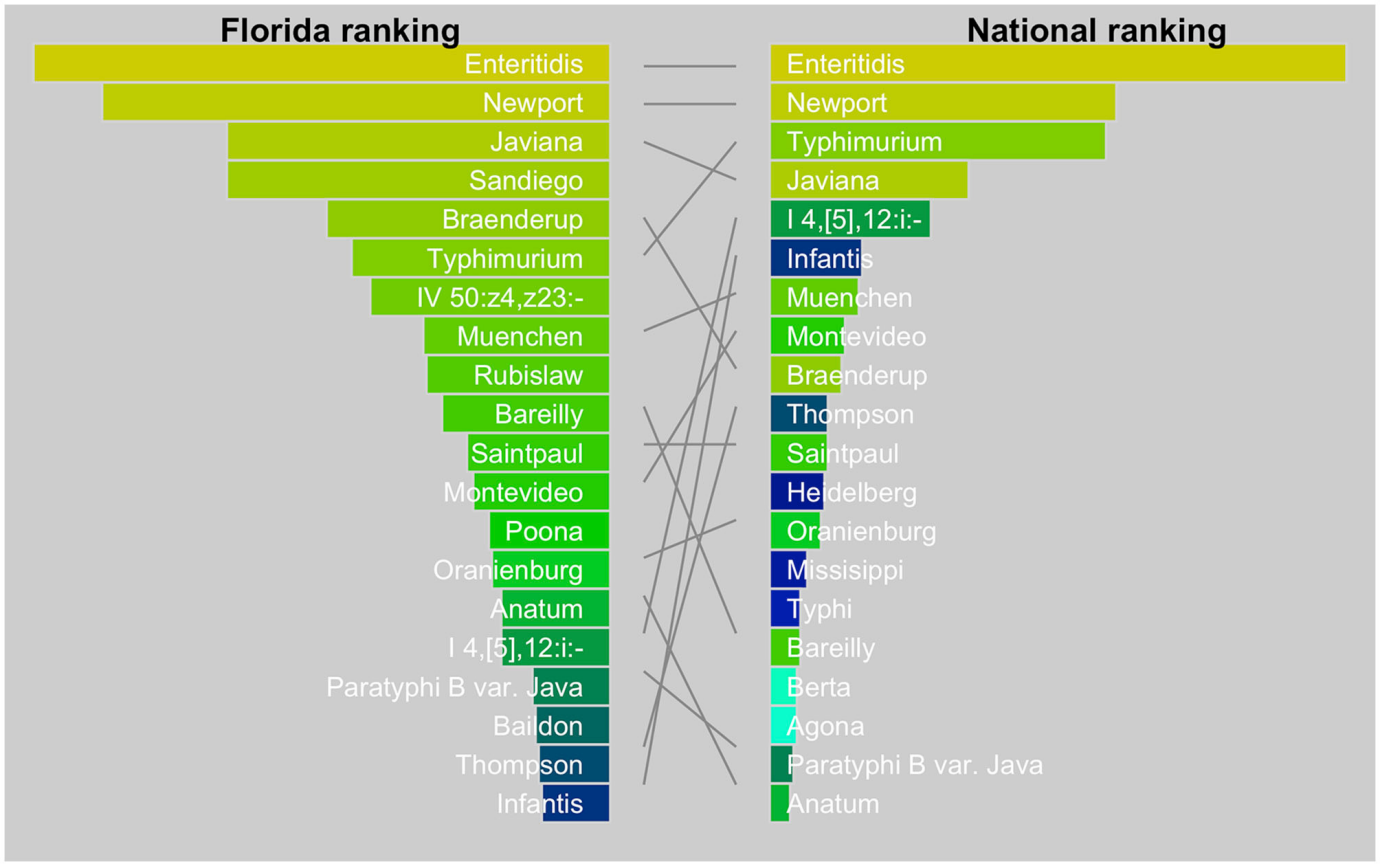
Tanglegram comparing top 20 *Salmonella* serotypes in Florida (2017–2018) with the USA (2016). The national data were from the National Enteric Disease Surveillance: Salmonella Annual Report, 2016 (Available at https://www.cdc.gov/nationalsurveillance/pdfs/2016-Salmonella-report-508.pdf) (accessed December 15, 2020). The width of the bars is scaled by the proportion of serotypes among all isolates.

The diversity of *Salmonella* isolates in Florida appeared to be higher than in the US as a whole (Figure 2). In our dataset, the top-5 serotypes comprised 27% of all isolates, whereas, in the US this was 47% (26). There is also a striking difference in the dominance of serotypes between Florida and the US. The top two serotypes were the same (Enteritidis and Newport) while Javiana ranked third in Florida *vs*. fourth in the US. Sandiego, the fourth ranking serotype in Florida did not even appear in the top-20 serotypes in the US. Likewise, serotype IV 50:z4, z23:- (formerly Flint) was a frequent occurrence in Florida but not nationally. On the other hand, serotypes I 4, [5],12:i:- and Infantis ranked relatively low in Florida.

### MultiLocus Sequence Typing

#### Legacy MLST Analysis

**Figure 3:**
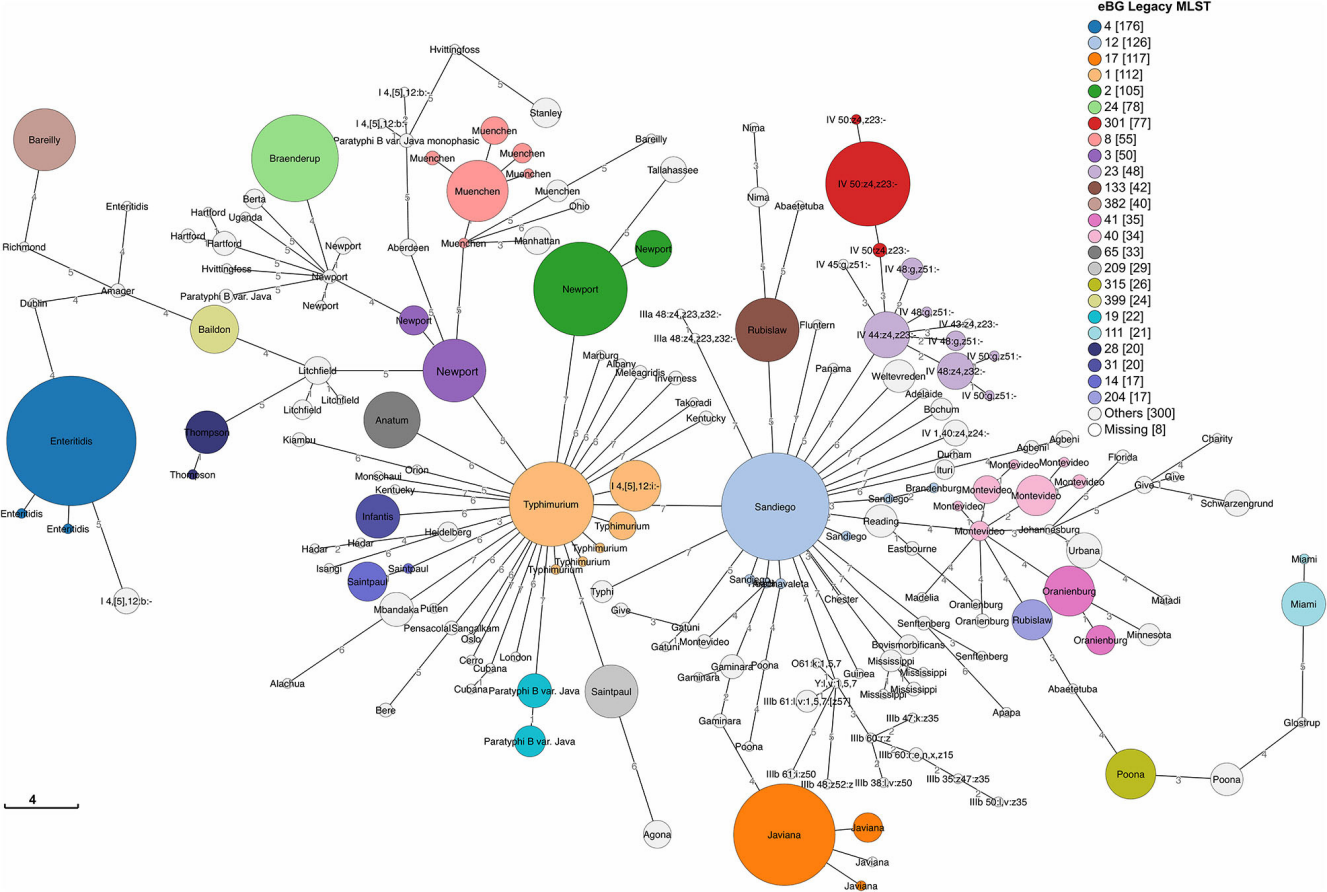
Minimum spanning tree of 1,632 sporadic clinical *S. enterica* isolates from Florida, USA 2017–2018 based on seven gene (legacy) MLST profiles. Top 24 eBG groups (representing 22 dominant serotypes according to rarefaction analysis) are colored and labeled with dominant serotypes in the eBG. Unlabeled STs groups share the dominant serotype with the adjacent ST in the same eBG. Legend shows number of isolates in corresponding eBG.

We detected 186 Sequence Types (ST) and 110 eBG among 1,632 sporadic isolates (Figure 3). The top 24 eBurst Groups (eBGs) associated with the 22 dominant serotypes and contributing STs are shown in Table 1. Many eBGs included only one serotype, and in most cases, one ST dominated in the eBG. For example, all except 2 *S*. Enteritidis isolates were ST11, *S*. Sandiego was dominated by ST126 while *S*. Javiana consisted mainly of ST24. *S*. Newport had most divergent ST types, including ST118, ST46, ST45, and ST5, and was spanning over two eBG, namely two and three. Note that *S*. Typhimurium and its monophasic variant (I 4, [5],12:i-) were included in the same eBG and both shared STs 19 and 34 interchangeably. eBG 23 included seven serotypes from *Salmonella enterica* subsp. *houtenae* (group IV). Serotypes like Rubislaw, Poona, and Saintpaul were grouped in two eBG.

**Table 1:** eBurst Groups (eBG) and sequence types (ST) corresponding to 22 dominant serotypes among 1,632 sporadic clinical *Salmonella* isolates collected during 2017–2018 in Florida, based on 7 gene MLST profiles.

**eBG**	**#Isolates in eBG**	Dominant serotype^*^	Other serotypes^*^	**Sequence type (#isolates in ST)**
4	176	Enteritidis		11 (174), 1925 (1), 1973 (1)
12	12	Sandiego	Arechavaleta Brandenburg Reading	126 (120), 2818 (1), 4026 (1), 5823 (1); 238 (1); 65 (1); 412 (1)
17	117	Javiana	-	24 (107), 1674 (9), 4750 (1)
1	112	Typhimurium	I 4, (5),12:i:-	19 (65), 34 (1), 213 (1), 313 (1), 3975 (8), 99 (1); 34 (26), 19 (9)
2	105	Newport	-	5 (14), 118 (91)
24	78	Braenderup	-	22 (78)
301	77	IV 50:z4, z23:-		2053 (74), 2405 (2), 4008 (1)
8	55	Muenchen		82 (1), 112 (39), 1567 (4), 2870 (8), 3795 (2), 4195 (1)
3	50	Newport		45 (9), 46 (41)
23	48	IV 48:g, z51:-	IV 48:z4,z32:- IV 44:z4,z23:- IV 50:g,z51:-	433 (14), 454 (1), 782 (1), 1869 (5); 2058 (14); 433 (8); 454 (3), 1209 (1), 2197 (1)
133	42	Rubislaw		94 (42)
382	40	Bareilly		2553 (40)
41	35	Oranienburg		23 (26), 1675 (9)
40	34	Montevideo		4 (8), 81 (17), 195 (4), 316 (2), 699 (1), 1531 (1), 4451 (1)
65	33	Anatum		64 (33)
209	29	Saintpaul		95 (29)
315	26	Poona		964 (26)
399	24	Baildon		2166 (24)
19	22	Paratyphi B		88 (10), 127 (12)
111	21	Miami	Sendai	80 (15), 4198 (1); 80 (5)
28	20	Thompson		26 (19), 4384 (1)
31	20	Infantis		32 (20)
14	17	Saintpaul		27 (1), 50 (16)
204	16	Rubislaw		1575 (16)
46	13	Poona		447 (11), 308 (1), 1069 (1)

* *Serotyped using SISTR (24)*.

#### Core Genome MLST Analysis

**Figure 4:**
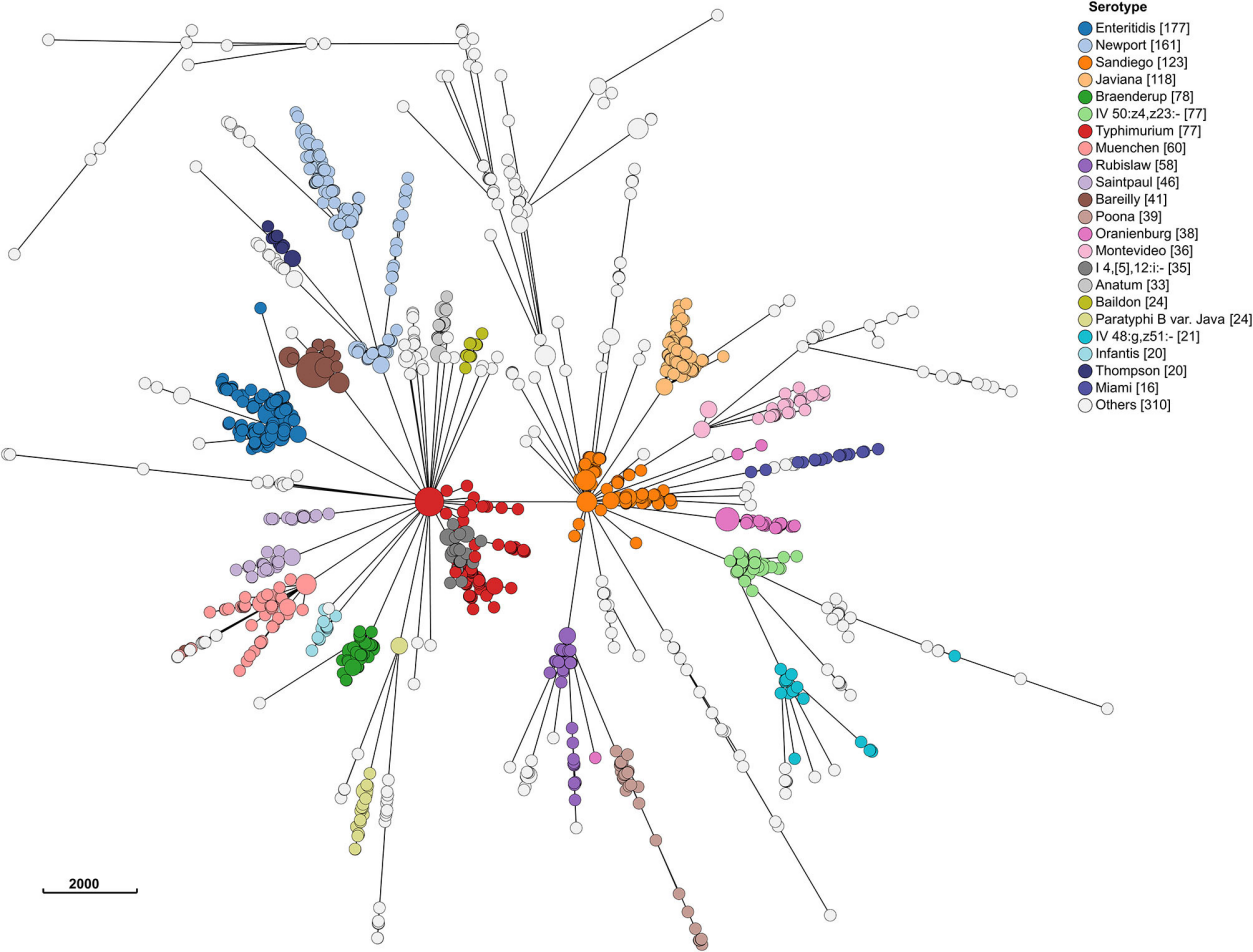
Minimum spanning tree of 1,632 sporadic clinical *S. enterica* isolates from Florida, USA 2017–2018 based on cg MLST profiles. Nodes are colored by dominant serotypes according to rarefaction analysis. Legend shows number of isolates in corresponding serotype.

Even at the cgMLST level, most serotypes were clustered together (Figure 4). Hierarchical clustering at HC2, HC5, HC10, and HC20 level identified 1,476, 1,360, 1,100, and 692 clusters, respectively. Of these, 29, 45, 77, and 104 included >2 isolates. MSTrees for all HC levels are provided in Supplementary Figure 1. The number of hierarchical clusters, even at the HC2 level, exceeded the number of detected outbreaks during the study period. This raised the question, which we investigated further, whether HC clustering can be a useful tool to select closely related isolates and reduce the size of isolate sets for more time consuming and computationally expensive SNP-based phylogenetic analysis. HC10 resulted in large clusters, and we considered this level as insufficiently discriminatory for outbreak detection, and based our analysis on HC5 clustering. HC2 clustering was evaluated after having performed phylogenetic analysis on all isolates in HC5 clusters (see section SNP Based Phylogenies and cgMLST Clustering for Outbreak Detection).

### SNP Based Phylogenies and cgMLST Clustering for Outbreak Detection

**Figure 5:**
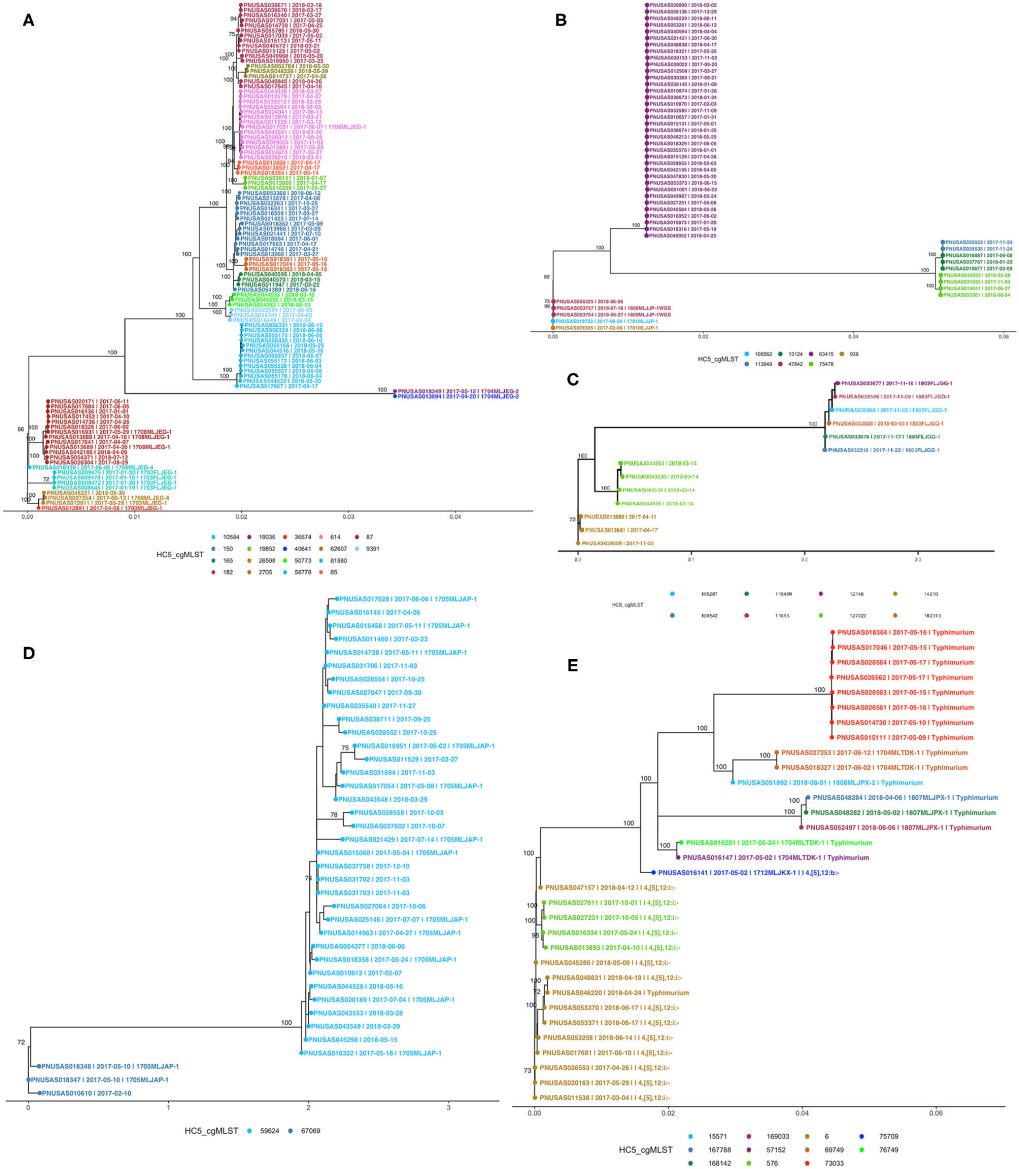
Phylogenetic trees of *Salmonella* isolates from five major serotypes in Florida, 2017–2018 from recognized outbreaks or by cgMLST HC5 clusters; **(A)** *S*. Enteritidis, **(B)** *S*. Newport, **(C)** *S*. Javiana, **(D)** *S*. Bareilly, **(E)** *S*. Typhimurium [including I 4i, [5], 12:i-]. Nodes are labeled with bootstrap support (≥ 70%). Tips are labeled by PNUSA number, isolate date and outbreak code (when available) and are colored by HC5 cluster.

We compared PFGE-based outbreak detection as implemented in Florida in 2017–2018 with the potential results that could have been obtained with cgMLST-based hierarchical cluster analysis. As a proof of principle, this comparison was restricted to the five most commonly detected serotypes while adding the monophasic variant of *S*. Typhimurium (I i4, [5], 12:i-). Figure 5 shows phylogenetic trees for each serotype; detailed SNP-distance trees for HC5 clusters are provided in the Supplementary Figures 2–6. The phylogenetic tree of 100 isolates of *S*. Enteritidis showed good agreement with HC5 clustering, including 14 well-segregated clades, with 5 large clusters (*87, 150, 182, 614, 10584*) comprising 13–14 isolates and 9 smaller clusters with 3–4 isolates. Isolates in all five large HC5 clusters had different SNP ranges, *87*: 0–24 SNPs, *150*: 0–21 SNPs, *182*: 0–28 SNPs, *614*: 0–16 SNPs, and *10584*: 0–12 SNPs. In all smaller clusters the isolates were 0–10 SNPs apart, ranging from 0–2 for HC5 *58778* and up to 4–10 for *50773*. In contrast, three smaller clades had multiple HC5 clusters: one, most distant from others, had 2 isolates in different HC5 clusters *19036, 40641* with 0 SNPs difference; second at the very bottom, had 4 isolates, 3 in HC5 *26508* within 2–5 SNPs and 1 at 12–14 SNPs from the rest in HC5 *536574*; third clade had HC5 cluster *58778* with 4 isolates within 0–2 SNPs and HC5 cluster *81880* at 70 SNPs form rest. Interestingly HC5 clusters *36574* and *81880* were labeled as same PFGE and were 33 SNPs apart. For *S*. Newport, three distant clades were observed corroborating their different eBG types. Among 50 isolates, 36 isolates corresponding to dominant eBG type clustered in one clade corresponding to HC5 cluster *63415*. The second clade included three HC5 clusters, *113949* and *13124* clustered together within 1–6 SNPs and the third cluster *75478* was ~250 SNPs apart from these. The third clade included five isolates, from two outbreaks (10–15 SNPs apart) and one related isolate. Three clades were observed for 13 isolates of *S*. Javiana; one clade included six isolates from six different HC5 clusters (2–31 SNPs apart) and two other clades included four and three isolates from the same HC5 cluster. Within these latter clusters, isolates were 8–27 SNPs different. Thirty-eight isolates of *S*. Bareilly clustered in two clades. One larger clade included 35 isolates from one HC cluster and included 12 isolates from recognized outbreaks. This cluster was quite heterogeneous with SNP distances ranging between 2 and 27. Interestingly, two isolates from one of these outbreaks and a related isolate were assigned to another HC5 cluster that was also clearly separated by SNP analysis. 17 isolates of *S*. Typhimurium clustered separately from 15 isolates of the monophasic variant *S*. I 4, [5],12:i:-, while one *S*. Typhimurium isolate clustered with the monophasic variant by both HC5 clustering and SNP analysis and *vice versa*. 8 *S*. Typhimurium isolates grouped in one clade, all belonging to the same HC5 cluster while the other isolates were singlets or doublets. Fifteen isolates of *S*. I 4, [5],12:i:- grouped in two clades supported by HC5 clustering, with one exception.

After comparing HC2 and HC5 cluster profiles (available in Supplementary Data Sheet 2) in all SNP -phylogenies, we observed that HC2 was too discriminatory, while HC5 clusters showed well-segregated clades, in SNP- based phylogenetic trees. Rationale for this can be given as 2 or less allelic difference at HC2 level was not sufficient to pick up enough SNP signals for creating reliable SNP phylogenies, while clustering at HC5 level provided an optimum solution for sample size and discriminatory power by SNP phylogeny.

We assigned isolates from clusters detected by either PFGE surveillance or HC5 clustering to different categories, depending on the degree of correspondence between different methods (Table 2). PFGE surveillance detected 12 clusters involved in recognized outbreaks (6 *S*. Enteritidis, 2 *S*. Newport, 1 *S*. Javiana, 2 *S*. Typhimurium, and 1 *S*. Bareilly).

1. Two outbreaks (1 *S*. Enteritidis, 1 *S*. Newport) included isolates within one HC5 cluster, and a maximum of 3 SNP distance.
2. Two outbreaks (1 *S*. Enteritidis, 1 *S*. Newport) were characterized by isolates with the same PFGE profile, and 0–2 SNP difference but nevertheless, belonged to different HC5 clusters. Isolates from the *S*. Enteritidis outbreak (1704MLJEG-2) were in the same HC10 cluster. Noticeably these two isolates were very distinct from the other 98 in *S*. Enteritidis isolates in the phylogenetic tree.
3. Four outbreaks (2 *S*. Enteritidis, 1 *S*. Javiana, 1 *S*. Typhimurium) included isolates with one PFGE profile, but two or more HC5 clusters that were supported by SNP analysis. Note that isolates from the two *S*. Enteritidis outbreaks (occurring ~2 months apart) had the same PFGE profile but belonged to three HC5 clusters.
4. One *S*. Typhimurium outbreak and one *S*. Bareilly outbreak included isolates that were different by PFGE profile, HC5 clusters, and SNP analysis. The *S*. Bareilly included isolates from patients up to 485 days apart. Within one HC5 cluster in this outbreak, there was one dominant PFGE profile, and isolates were up to 15 SNP different. The second HC5 cluster had the same PFGE profile but a different HC5; these isolates were more than 40 SNP different from the core cluster.
5. All isolates from five HC5 clusters were collected within a 60-day window (range 1–28 days) which is commonly used for detecting outbreaks of *Salmonella* (20). Three of these clusters showed more than one PFGE profile.
6. There were two *S*. Enteritidis outbreaks with multiple PFGE profiles but one HC5 cluster, supported by phylogenetic analysis. The HC5 clusters included 10 and 13 additional isolates, all closely related by SNP distance. Isolates in these clusters were obtained over a time span of 451 and 558 days, respectively.
7. We detected 14 HC5 clusters [8 *S*. Enteritidis, 3 *S*. Newport, 1 *S*. Javiana, and 2 *S*. i4, (5),12:i-] that were not included in previously recognized outbreaks, all supported by phylogenetic analysis. These clusters were prolonged in time (range between first and last isolate in the cluster 63 – 558 days) and 12 of these clusters showed more than 1 PFGE profile.

**Table 2:** Comparison of cgMLST and PFGE based outbreak detection for *Salmonella* in Florida, 2017–2018.

**Outbreak**	**HC5 cluster**	**Serotype**	**PFGE**	**^**^#^**^Ob**	**^**^#^**^Nob**	**First date**	**Last date**	**Duration** **(days)**	**Comments**
**1. Outbreak detected by PFGE, confirmed by HC5 and SNP**									
1703FLJEG-1	*58778*	Enteritidis	JEGX01.1057	4	0	20170119	20170120	2	1-2 SNPs
1809MLJJP-1WGS	*47842*	Newport	JJPX01.0010	2	1	20180606	20180718	43	1 additional case detected by HC5 (2–5 SNPs)
**2. Outbreak detected by PFGE, confirmed by SNP, more than one HC5**									
1704MLJEG-2	*40641* *19036*	Enteritidis	JEGX01.0049 JEGX01.0049	1 1	0 0	20170420	20170512	23	0 SNPs
1701MLJJP-1	*938* *106562*	Newport	JJPX01.0010 JJPX01.0010	1 1	0 0	20170206	20170624	139	1 SNPs
**3. Outbreak detected by PFGE, not confirmed by HC5 and SNP**									
1703MLJEG-1	*26508* *36574*	Enteritidis	JEGX01.0031 JEGX01.0031	1 1	0 0	20170328 20170408		12	2 HC5, 12 SNPs
1708MLJEG-4	*81880* *26508* *26508*	Enteritidis	JEGX01.0031 JEGX01.0031 JEGX01.0031	1 1 0	0 0 1	20170605 20170612 20180530		8	2 HC5, 46 SNP. One related isolate (3 SNPs) detected approx. 1 year later
1803FLJGG-1	*105287* *11615* *12146* *115499* *109542* *182313*	Javiana	JGGX01.0094 JGGX01.0094 JGGX01.0094 JGGX01.0094 JGGX01.0094 JGGX01.0094	1 1 1 1 1 1	0 0 0 0 0 0	20171102	20180305	124	6 HC5, 12–31 SNPs with 1 exception (2 SNP)
1807MLJPX-1	*167788* *168142* *169033*	Typhimurium	JPXX01.0003 JPXX01.0003 JPXX01.0003	1 1 1	0 0 0	20180406	20180606	62	3 HC5, 5–16 SNP
**4. Outbreak detected, not confirmed by PFGE, HC5 and SNP**									
1704MLTDK-1	*69749* *76749* *57152*	Typhimurium	JPXX01.0033 JPXX01.0033 JPXX01.0351	2 1 1	0	20170602 20170524 20170502	20170612	42	2 PFGE, 2 HC5, 2 SNP clusters (333 SNPs apart)
1705MLJAP-1	*59624* *67069*	Bareilly	JAPX01.0064 JAPX01.0216 NA	12	21 2 1	20170207	20180606	485	2 PFGE, 2 HC5, > 47 SNPs between clusters
**5. Outbreak not detected by PFGE, one cluster by HC5 and supported by SNP, all cases within 60 days**									
-^&^	*85*	Enteritidis	JEGX01.0004 JEGX01.0021	0 0	1 2	20170417	20170514	28	2 PFGE, 1–4 SNPs
-	*2705*	Enteritidis	JEGX01.0004 JEGX01.0034	0 0	2 1	20170516	20170516	1	2 PFGE, 1–2 SNPs
-	*113949*	Newport	JPX01.0036 JJPX01.0038	0	2 1	20171124	20171124	1	2 PFGE, 1 SNP
-	*127322*	Javiana	JGGX01.0012	0	4	20180314	20180314	1	1 PFGE, 1 HC5, 8–27 SNPs
-	*73033*	Typhimurium	JPXX01.5110 NA	0	7 1	20170509	20170517	9	1 PFGE, 0–4 SNPs
**6. Outbreak detected, multiple PFGE profiles, one HC5 cluster supported by SNP; prolonged case series**									
1706MLJEG-1	*614*	Enteritidis	JEGX01.0023 JEGX01.0021 JEGX01.0004 JEGX01.0034	1 0 0 0	10 2 1	20170312	20180605	451	1 recognized outbreak isolate (5–8 SNPs from 8 isolates), 4 PFGE
1708MLJEG-1	*87*	Enteritidis	JEGX01.0002 JEGX01.0020 JEGX01.0024 JEGX01.0184 JPPX01.0001	3	6 1 1 1 1	20170101	20180712	558	3 recognized outbreak isolates ( 2 at 0–7 SNPs from 6 isolates), 5 PFGE
**7. Outbreak not detected, multiple PFGE profiles, one HC5 cluster supported by SNP; prolonged case series**									
-	*62607*	Enteritidis	JEGX01.0021	0	3	20170426	20180530	400	1 PFGE, 2–7 SNPs
-	*182*	Enteritidis	JEGX01.0021 JEGX01.0023 JEGX01.0004	0 0 0	12 1 1	20170323	20180530	434	3 PFGE, multiple small SNPs clusters, maximum distance 28 SNPs
-	*19852*	Enteritidis	JEGX01.0021 JEGX01.0004	0 0	2 1	20170107	20170527	141	2 PFGE, 0 SNPs
-	*165*	Enteritidis	JEGX01.0004 JEGX01.0034	0 0	2 1	20170322	20180405	380	2 PFGE, 2–6 SNPs
-	*150*	Enteritidis	JEGX01.0004 JEGX01.0034	0 0	11 3	20170327	20180612	443	2 PFGE, multiple small SNPs clusters, maximum distance 21 SNPs
-	*9391*	Enteritidis	JEGX01.0004	0	3	20170404	20170605	63	1 PFGE (1–4 SNPs)
-	*50773*	Enteritidis	JEGX01.0004	0	3	20180315	20180613	91	1 PFGE (4–10 SNPs)
-	*10584*	Enteritidis	JEGX01.0005 JEGX01.0030	0	12 1	20170417	20180612	422	2 PFGE (0–12 SNPs)
-	*75478*	Newport	JJPX01.0384	0	4	20170627	20180623	362	1 PFGE (2–4 SNPs)
	*63415*	Newport	JJPX01.3716 NA JAAX01 JEGX01.0005 JGXX01.0006 JJPX01.5786 JLXX01.0483	0	28 3 1 1 1 1 1	20170126	20180623	514	≥7 PFGE profiles, many close clusters (22 isolates 0–7 SNPs), maximum distance 19 SNPs
-	*13124*	Newport	JJPX01.0032 JJPX01.0036 JJPX01.0400	0	1 1 1	20170309	20180125	323	3 PFGE, 4 SNPs
-	*14210*	Javiana	JGGX01.0012 JGGX01.0488 JGGX01.0013	0	1 1 1	20170411 20170417 20171105	20171105	209	3 PFGE profiles, 9–16 SNP
-	*576*	I 4, [5],12:i:-	JPXX01.2583 JPXX01.3877		2 2	20171001 20170410	20171005 20170524	179	2 PFGE, 0–10 SNP
-	*6*	I 4, [5],12:i:-	JPXX01.3034 JPXX01.1314 JPXX01.1314 JPXX01.3775 JPXX01.5522^*^	0	1 3 2 2 2 1	20170304 20170426 20180509 20180617 20180419 20180412	20170610 20180614 20180424	468	6 PFGE, 3 SNP clusters

#Ob, number of outbreak isolates;

# Nob, number of non-outbreak isolates.

& No outbreak detected.

* *1 isolate serotyped as Typhimurium*.

### Spatial Distribution of HC5 Clusters

**Figure 6:**
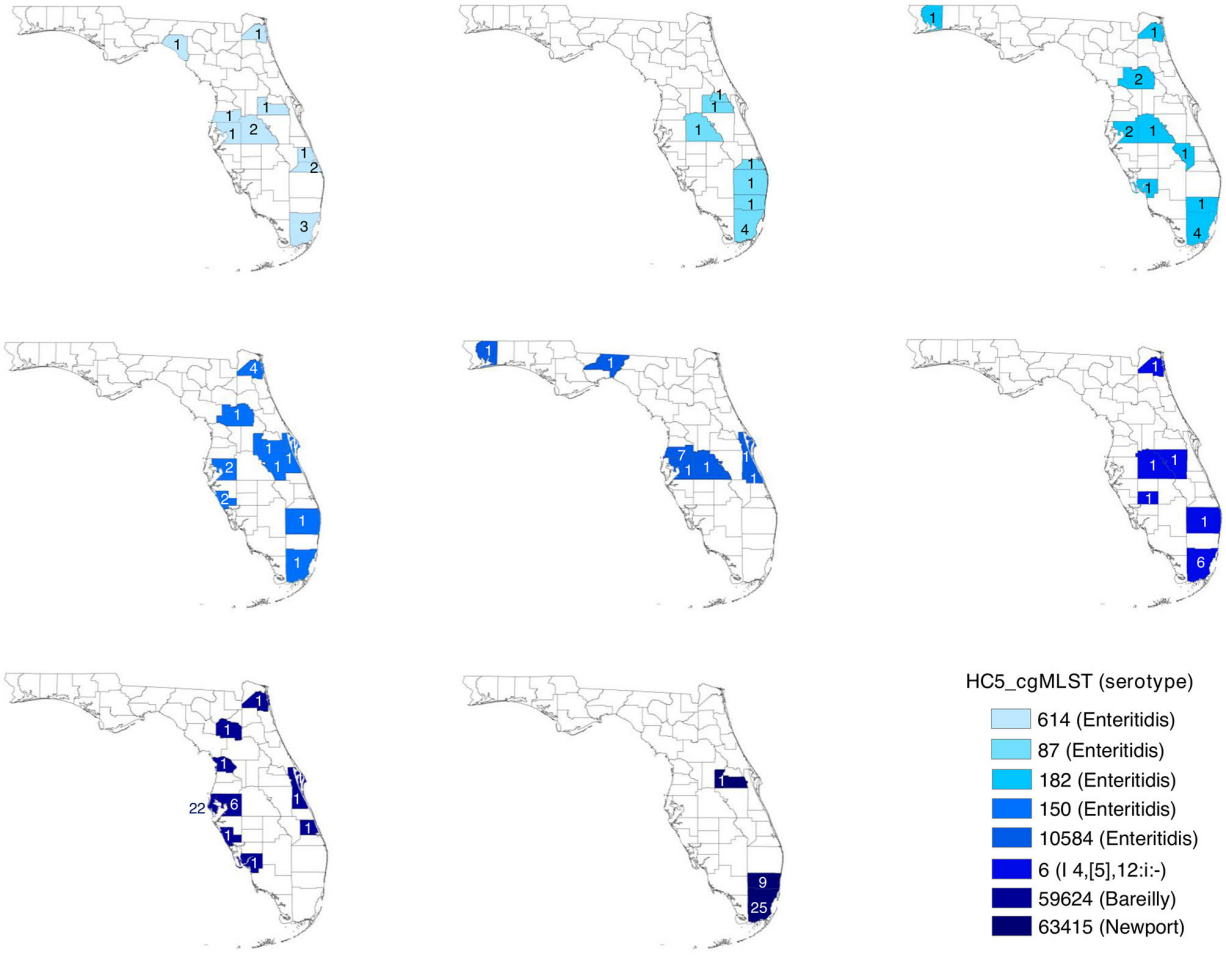
Spatial distribution of prolonged case series of *S. enterica* in Florida, USA 2017–2018; detected by hierarchical clustering of cgMLST profiles. Counties are colored by HC5 cluster and are labeled with the number of isolates detected in the corresponding county. Zip code information were not available for 5 isolates in 3 HC5 clusters: 1 in *614*, 3 in *87*, and 1 in *63415*.

Geospatial analysis of eight large (more than 10 isolates) prolonged case series of clinical isolates, detected by HC5 clustering, and confirmed by SNP analysis, suggested that while most clusters were confined to a specific region in Florida, there were considerable distances between the cases (Table 3, Figure 6, and Supplementary Figure 7). Clusters involved between 10 and 26 zip-code areas, and the standard distance of zip-code areas in each cluster varied between 66 and 244 km. Only three of these clusters were detected as outbreaks by PFGE surveillance.

**Table 3:** Geospatial distribution of 8 prolonged cases series (>10 isolates) based on HC5 clusters in Florida, 2017–2018.

**Serotype**	**HC5 cluster**	**#Isolates**	**#Zip code areas**	**Standard distance (km)**
Bareilly	59624^*^	35	25	92
Enteritidis	87^*^	13	11	155
	*150*	14	14	173
	*182*	14	14	244
	614^*^^&^	14	12	177
	*10584*	13	10	202
I 4, [5],12:i:-	*6*	11	11	173
Newport	*63415*	36	26	66

* Outbreak detected by PFGE surveillance, additional cases detected by HC5 clustering.

& *Prolonged case series*.

### Association of Clinical and Non-clinical Isolates

#### Florida Clusters

Source and serotype based color-coded MSTrees for the isolate set “sporadic clinical + non-clinical” is shown in Supplementary Figure 8. We detected 12 HC5 clusters that included isolates from clinical and non-clinical sources representing six serotypes (Table 4).

**Table 4:** Source matching between clinical and non-clinical isolates from Florida based on HC5 clusters.

**Serotype**	**HC5 Cluster**	**Source**	**#Isolates**
Braenderup	*68954*	Human	3
		Poultry	1
Enteritidis	*150^&^*	Human	14
		Poultry	4
	*182^&^*	Human	14
		Poultry	9
	*614^&^*	Human	14
		Poultry	1
	*3213*	Human	2
		Poultry	1
	*10584^&^*	Human	13
		Poultry	7
I 4, [5],12:i:-	*576*	Human	4
		Food	1
		Livestock	1
	*118459*	Human	1
		Poultry	1
Infantis	*4584*	Human	1
		Poultry	2
	*106860*	Human	1
		Poultry	1
Saintpaul	*9009*	Human	2
		Environment	1
Sandiego	*136994*	Aquatic Animal	2
		Non-clinical sponge	1

& *Prolonged case series*.

**Figure 7:**
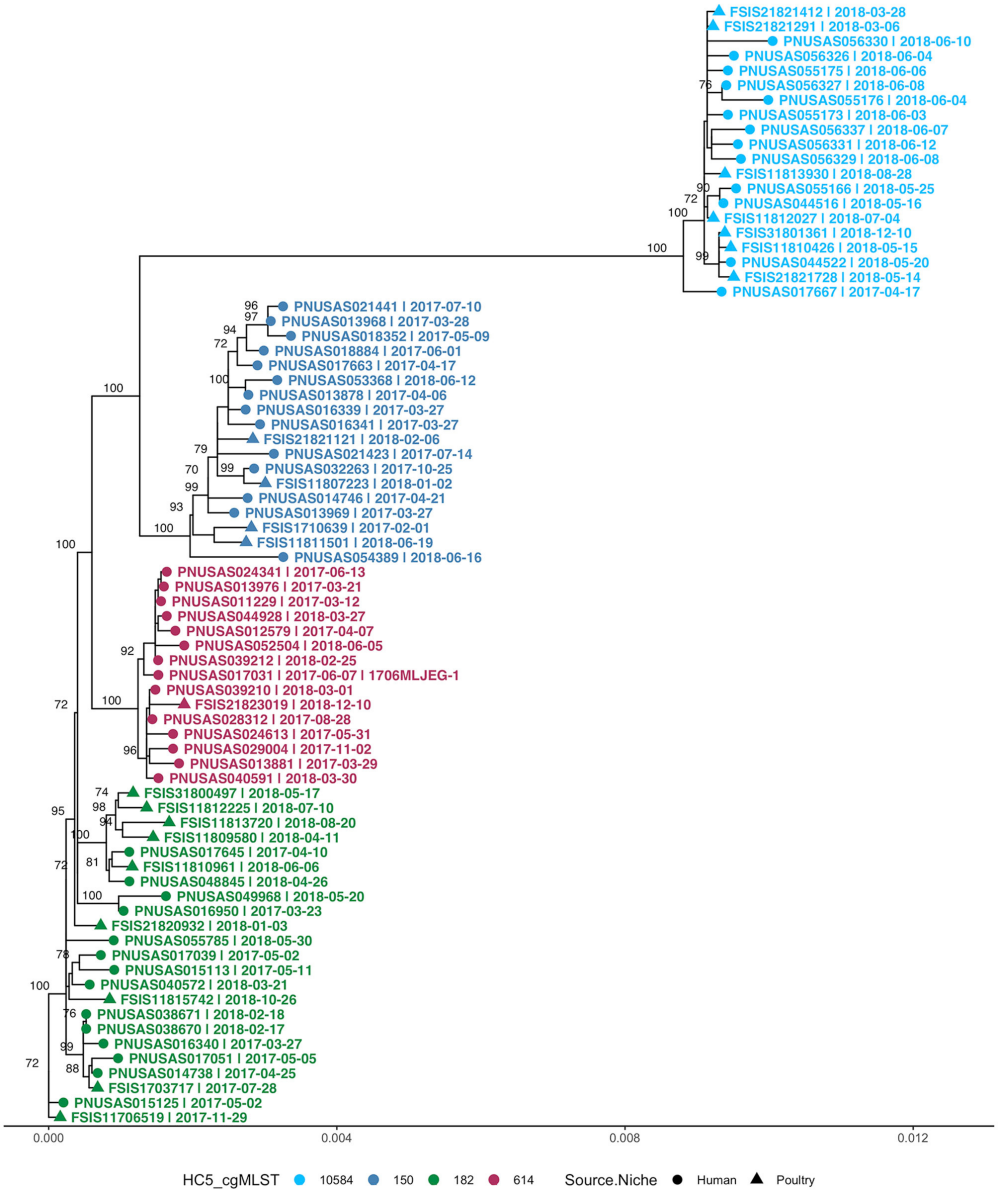
Phylogenetic tree of *S*. Enteritidis isolates from clinical and non-clinical (poultry) sources in Florida, collected during 2017–2018, including four cgMLST HC5 clusters with isolates from both sources. Nodes are labeled with bootstrap support (≥70%). Tips are labeled by PNUSA number (clinical isolates, dots), FSIS number (non-clinical isolates, triangles), isolate date and outbreak code (when available), and are colored by HC5 cluster.

Four clusters belonged to *S*. Enteritidis and all were prolonged case series as described above. All non-clinical isolates in these *S*. Enteritidis clusters were isolated from poultry. In total, these clusters (>2 clinical isolates) comprised 55 clinical and 21 non-clinical isolates and were investigated by SNP-based phylogeny as shown in Figure 7. A SNP distance heatmap for all 4 clusters is included as Supplementary Figure 9. Two clusters (*150* and *182*) were quite heterogeneous with SNP distances up to 30, while in one cluster (*10584*) SNP distances up to 13 were observed. Within each of these clusters, smaller subclusters with highly related human and poultry isolates were detected. One cluster (*614*) separated into two subclusters of which one included a poultry isolate.

#### Multistate S. Enteritidis Clusters

Because a large proportion of poultry meat consumed in Florida is produced in other states, we extended our HC5 profile comparison of 176 clinical *S*. Enteritidis isolates from Florida with 1,341 non-clinical isolates from across the USA (“*Enteritidis poultry” set*) and detected 8 HC5 clusters (>2 clinical isolates) including 67 clinical and 513 poultry isolates (21: Florida, 492: other states). Four of these clusters, *10584, 150, 182*, and *614*, were already identified as including poultry isolated from Florida, and increased with an additional 1, 171, 237, and 31 isolates from other states, respectively. Four additional HC5 clusters: *165* (3 clinical, 19 poultry), *62607* (3 clinical, 6 poultry), *85* (3 clinical, 15 poultry), and *9391* (3 clinical, 12 poultry) were detected in which all poultry isolates were from other states.

**Figure 8:**
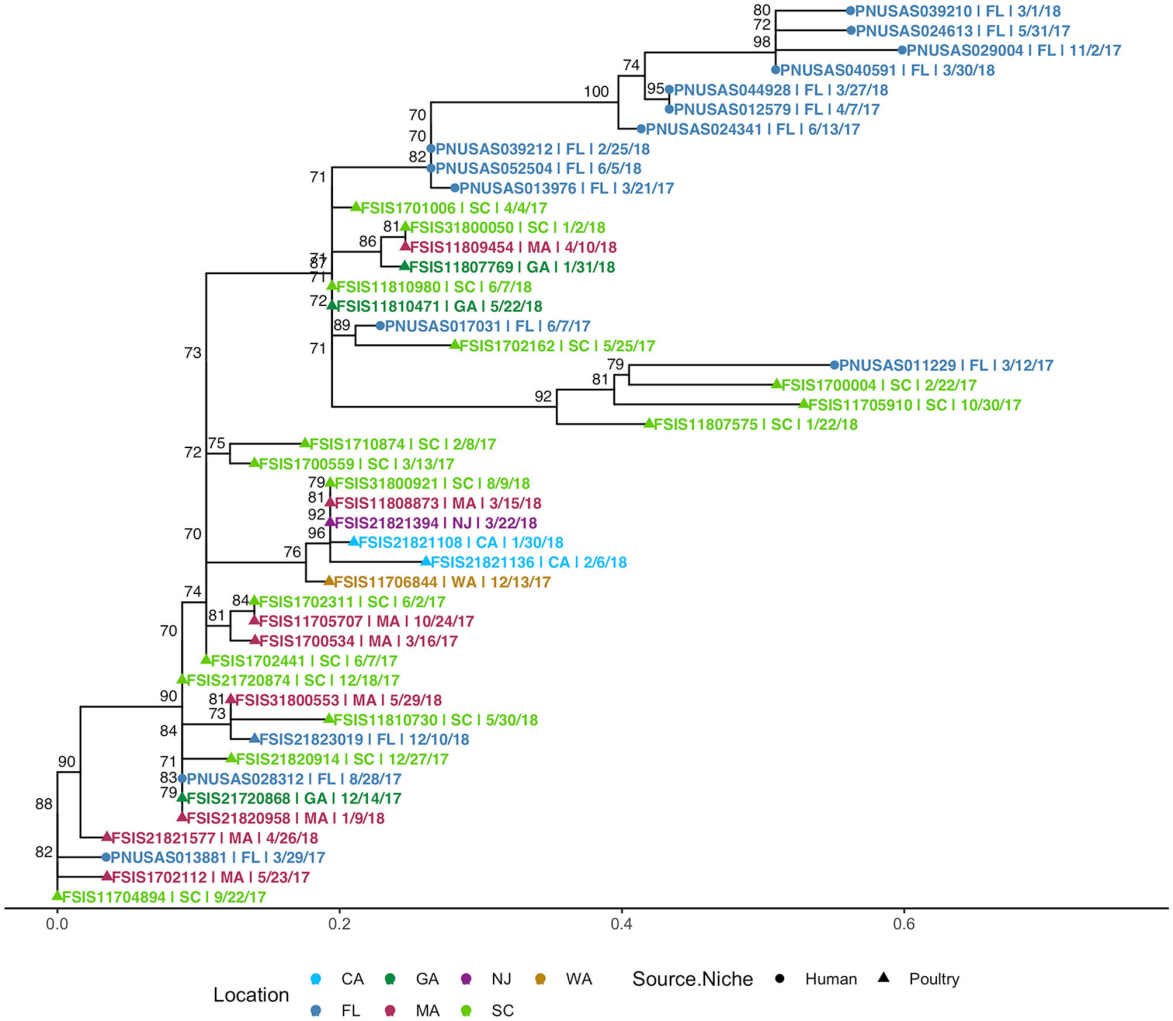
Phylogenetic tree of *S*. Enteritidis isolates clustered in HC5 614 cluster from clinical sources in Florida and non-clinical (poultry) sources in the USA, collected during 2017–2018. Nodes are labeled with bootstrap support (≥70%). Tips are labeled by PNUSA number (clinical isolates, dots), FSIS number (poultry isolates, triangles), USA State of sample collection, and isolate date and are colored by USA State of sample collection; CA, California; GA, Georgia; NJ, New Jersey; WA, Washington; FL-Florida; MA, Massachusetts; SC, South Carolina.

SNP-based phylogeny and maximum likelihood phylogenetic trees for 7 clusters are included in Supplementary Figure 10. A phylogenetic tree for HC5 cluster *614*, comprising 31 poultry isolates from 6 other states and 1 isolate from Florida, along with 14 clinical isolates from Florida, is depicted in Figure 8. SNP-distances in this cluster were in a broad range (0–35). The majority of clinical isolates (8) were clustered separately (5–15 SNP apart) from poultry isolates, while three isolates clustered closely (0–4 SNP distance) in three smaller subclusters of 3–11 isolates and three isolates clustered together with two poultry isolates. All other seven clusters had variety of SNP distance ranges, commonly up to 30, while large clusters like 150 and 182 included distant isolates of >30 SNP as well, as depicted in the SNP-distance heatmaps for all eight clusters in Supplementary Figure 11.

**Figure 9:**
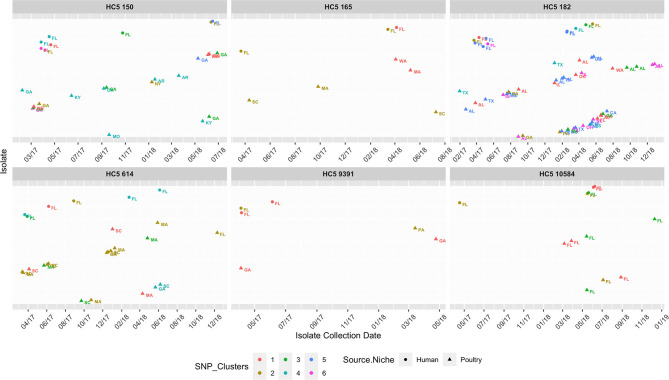
Isolate collection time series (on x-axis for 01/01/2017-12/31/2018) of isolates (y-axis as presence of isolates*), collected from clinical sources in Florida and non-clinical (poultry) sources in the USA, faceted for 8 HC5 clusters, depicting their corresponding SNP clusters (0–4 SNP distance). Isolates are represented as circle (clinical) and triangles (poultry), text labeled by USA State of sample collection and colored by SNP cluster (numbered specific to each HC5 cluster independently). Isolates are spaced along the y-axis and names are dropped for readability.

As *S. Enteritidis* is a highly clonal organism, to further zoom in to highly related clusters, we used a small range of 0–4 SNP distance as cutoff and obtained 2–6 close SNP clusters within all HC5 clusters, except *62607* and *85*. Table 5 enumerates clinical and non-clinical isolates from SNP sub-clusters in each HC5 cluster and involved HC2 clusters in each SNP cluster and Figure 9 depicts their time-series, stratified by state and collection source. In HC5 cluster *150*, all 6 SNP clusters were spread over time in 2017 and 2018 and suggested genetic relatedness of Florida clinical isolated with poultry isolates collected from 8 states, mainly Georgia. For HC5 cluster *165*, one SNP cluster was close in time, but poultry isolates were collected form two distant states and a second SNP cluster was spread out in time. HC5 cluster *182* had six large, interesting SNP clusters, comprising poultry, and clinical isolates collected in both 2017 and 2018. HC5 cluster *10584* had poultry isolates only from Florida in all 3 SNP clusters mainly collected in a span of 2–6 months in 2018 while HC5 cluster *9391* had three time-sparse SNP clusters with poultry isolates form Georgia and Pennsylvania. HC5 Cluster *614 is* heterogenous in terms of states of poultry isolates but SNP clusters shared time proximity with clinical isolates. Of 24 detected SNP clusters, 10 included only one HC2 cluster profile, while the remaining 14 SNP clusters included between 2 and 8 HC2 clusters, confirming our choice of HC5 as the optimal level of clustering.

**Table 5:** SNP clusters^†^ (0–4 SNP difference) of clinical isolates from Florida and poultry isolates of *Salmonella* Enteritidis from the USA, within HC 5 clusters.

**HC5 cluster**	**HC2 cluster** **(# of isolates)**	**SNP cluster^†^**	**Clinical isolates**	**Number of poultry isolates by state**
150	8440 (4)	1	PNUSAS014746 (FL|04/21/2017)	FSIS1700341 (MS|03/08/2017) FSIS31800585 (WA|06/04/2018) FSIS31800622 (WA|06/07/2018)
	8440 (3), 169289 (1)	2	PNUSAS053368 (FL|06/12/2018) PNUSAS013878 (FL|04/06/2017)	FSIS1700715 (GA|03/22/2017) FSIS21820955 (NY|01/09/2018)
	68567 (5)	3	PNUSAS032263 (FL|10/25/2017)	FSIS1700276 (GA|03/07/2017) FSIS21720256 (GA|09/13/2017) FSIS11811144 (GA|06/07/2018) FSIS31800742 (GA|06/28/2018)
	8440 (4), 72875 (1), 77030 (1), 88317 (1), 115025 (1), 126031 (1)	4	PNUSAS017663 (FL|04/17/2017) PNUSAS016341 (FL| 03/27/2017)	FSIS21821015 (AR|01/17/2018) FSIS21821366 (AR|03/20/2018) FSIS11810460 (KY|05/21/2018) FSIS1702583 (KY|06/14/2017) FSIS21720225 (OK|09/06/2017) FSIS11704839 (MO|09/20/2017) FSIS1710748 (GA|02/06/2017)
	123047 (2)	5	PNUSAS054389 (FL|06/16/2018)	FSIS31800470 (GA|05/09/2018)
	68858 (2)	6	PNUSAS013969 (FL|03/27/2017)	FSIS1700247 (OK|03/05/2017)
165	165 (3)	1	PNUSAS040595 (FL|04/05/2018)	FSIS21821669 (MA|05/09/2018) FSIS31800337 (WA|04/03/2018)
	165 (3), 138945 (1), 93887 (1)	2	PNUSAS040570 (FL|03/15/2018) PNUSAS011947 (FL|04/22/2017)	FSIS11812199 (SC|07/10/2018) FSIS1701123 (SC|04/11/2017) FSIS21720358 (MA|09/26/2017)
182	31321 (9), 135578 (1)	1	PNUSAS017645 (FL|04/10/2017)	FSIS11811483 (WA|06/09/2018) FSIS21821927 (WA|07/13/2018) FSIS11810961 (FL|06/06/2018) FSIS21820940 (IL|01/04/2018) FSIS21821386 (OK|03/21/2018) FSIS31800336 (AL|04/03/2018) FSIS11811422 (AL|06/09/2018) FSIS1700876 (AL|003/28/2017) FSIS21720176 (AL|08/29/2017)
	60809 (2), 83883 (3)	2	PNUSAS049968 (FL|05/20/2018) PNUSAS016950 (FL|03/23/2017)	FSIS1703737 (GA|07/24/2017) FSIS11704677 (GA|09/13/2017) FSIS11807603 (PA|01/23/2018)
	117653 (5)	3	PNUSAS048845 (FL|04/26/2018)	FSIS11808374 (MA|02/21/2018) FSIS11811751 (GA|06/26/2018) FSIS21822276 (AL|09/19/2018) FSIS21822629 (AL|10/24/2018)
	3334 (4)	4	PNUSAS040572 (FL| 03/21/2018)	FSIS1710634 (TX|01/31/2017) FSIS31800047 (TX|01/04/2018) FSIS11810277 (MS|05/15/2018)
	41659 (18), 125926 (1), 126076 (1)	5	PNUSAS038671 (FL|02/18/2018) PNUSAS038670 (FL| 2/17/2018) PNUSAS016340 (FL|03/27/2017) PNUSAS014738 (FL|04/25/2017) PNUSAS017051 (FL|05/05/2017)	FSIS11812210 (CA|07/10/2018) FSIS11810099 (NJ|05/10/2018) FSIS11810454 (MI|05/18/2018) FSIS31800499 (CO|05/17/2018) FSIS31800539 (AL|05/24/2018) FSIS21820981 (AL|01/10/2018) FSIS21821128 (AL|02/06/2018) FSIS11808362 (MS|02/20/2018) FSIS1703451 (MS|07/18/2017) FSIS21821090 (TX|01/31/2018) FSIS11807765 (TX|01/30/2018) FSIS11808961 (TX|03/20/2018) FSIS1701679 (TX|05/03/2017) FSIS1703717 (FL|07/28/2017) FSIS1700022 (AL|02/22/2017)
	64255 (7), 161204 (1), 164058 (1)	6	PNUSAS015113 (FL|05/11/2017)	FSIS11704369 (AL|08/28/2017) FSIS21923137 (AL|12/19/2018) FSIS21923159 (AL|12/27/2018) FSIS11809591 (OH|04/16/2018) FSIS11810473 (NC|05/22/2018) FSIS1703098 (IN|07/05/2017) FSIS21821483 (IL|04/11/18/2018) FSIS11808858 (IL|03/14/2018)
614	614 (6)	1	PNUSAS017031 (FL|06/07/2017)	FSIS1701006 (SC|04/04/2017) FSIS11810980 (SC|06/07/2018) FSIS31800050 (SC|01/02/2018) FSIS11809454 (MA|04/10/2018) FSIS11810471 (GA|05/22/2018)
	614 (5), 68955 (1), 72379 (1), 98146 (1), 103657 (1), 106428 (1), 152255 (1), 163446 (1)	2	PNUSAS028312 (FL|08/28/2017)	FSIS11705707 (MA|10/24/2017) FSIS1700534 (MA|03/16/2017) FSIS1700559 (SC|03/13/2017) FSIS1702311 (SC|06/02/2017) FSIS1702441 (SC|06/07/2017) FSIS21720874 (SC|12/18/2017) FSIS21820914 (SC|12/27/2017) FSIS21720868 (GA|12/14/2017) FSIS21820958 (MA|01/09/2018) FSIS31800553 (MA|05/29/2018) FSIS21823019 (FL|12/10/2018)
	614 (4)	3	PNUSAS013881 (FL|03/29/2017)	FSIS11704894 (SC|09/22/2017) FSIS1702112 (MA|05/23/2017) FSIS21821577 (MA|04/26/2018)
	614 (5)	4	PNUSAS052504 (FL|06/05/2018) PNUSAS039212 (FL|02/25/2018) PNUSAS013976 (FL|03/21/2017)	FSIS11810471 (GA|05/22/2018) FSIS11810980 (SC|06/07/2018)
9391	9391 (1), 86566 (1), 119878 (1), 74731 (1)	1	PNUSAS022594 (FL|06/05/2017) PNUSAS014741 (FL|04/05/2017)	FSIS21821561 (GA|04/24/2018) FSIS1701015 (GA|04/04/2017)
	62823 (1), 78219 (1)	2	PNUSAS016449 (FL|04/04/2017)	FSIS31800280 (PA|03/14/2018)
10584	10584 (5), 12492 (1), 151970 (1)	1	PNUSAS056327 (FL|06/08/2018) PNUSAS055176 (FL|06/04/2018) PNUSAS017667 (FL|04/17/2017)	FSIS21821412 (FL|03/28/2018) FSIS21821291 (FL|03/06/2018) FSIS11812027 (FL|07/04/2018) FSIS11813930 (FL|08/28/2018)
	10584 (2), 12492 (1)	2	PNUSAS044516 (FL|05/16/2018) PNUSAS017667 (FL|04/17/2017)	FSIS11812027 (FL|07/04/2018)
	10584 (4)	3	PNUSAS044522 (FL|05/20/18)	FSIS31801361 (FL| 12/10/2018) FSIS11810426 (FL|05/15/ 2018) FSIS21821728 (FL| 05/14/2018)
85			No cluster	
62607			No cluster	

† *Numbered independently for each HC5 cluster. AR, Arkansas; AL, Alabama; CA, California; CO, Colorado; GA, Georgia; IL, Illinois; IN, Indiana; KY, Kentucky; MA, Massachusetts; MI, Michigan; MS, Mississippi; NC, North Carolina; NJ, New Jersey; OK, Oklahoma; OH, Ohio; PA, Pennsylvania; TX, Texas; WA, Washington; FL, Florida; MA, Massachusetts; SC, South Carolina; WA, Washington*.

To further evaluate genetic relatedness between clinical and poultry isolates, we searched for the presence of genes conferring antimicrobial drug resistance in all 580 isolates in the eight common HC5 clusters, *via* AMRFinder. It is notable that AMRFinder considers complete genome information along with plasmids while HC5 clustering only considers the core genome. Twenty-four isolates carried the tetracycline resistant gene *tet(A)* and, out of these eight clustered in HC5 *62607* (poultry: 6 and human: 2) and two clustered in HC 5*150* and none of these 10 isolates were assigned to close SNP clusters. The other 14 isolates carried additional genes for beta-lactamase resistance (*bla*_*TEM*−1_), sulphonamide resistance (*sul2*), aminoglycoside 3'-phosphotransferase (*aph(3”)-Ib*) and aminoglycoside O-phosphotransferase (*aph(6)-Id*). All 14 isolates belonged to HC5 *9391* and six multi-drug resistant isolates were assigned to two SNP clusters (one cluster with two human and two poultry isolates, and one cluster with one human and one poultry isolate, depicted in Figure 9). A detailed table is included in Supplementary Data Sheet 3.

## Discussion

We present a retrospective analysis of whole genome sequencing data of *Salmonella enterica* isolates from Florida in the years 2017–2018. Our earlier work has shown that the sequenced isolates in 2017–18 were a relatively unbiased sample of confirmed clinical cases (4). The population structure of *Salmonella* is Florida was found to be highly diverse with 115 serotypes observed in a set of 1,632 sporadic clinical isolates obtained over this 2-year period. A larger dataset is expected to include a much higher number of serotypes as the rarefaction curve for serotype richness was far from reaching an asymptote. However, many serotypes, including unsampled ones, were rare as demonstrated by the much lower number of 36 typical serotypes, and 22 dominant serotypes. Both of these are well-sampled as demonstrated by plateaus reached in the rarefaction curves. As little as 250 isolates appeared to be sufficient to identify the dominant serotypes. Serotype diversity was similar in young children (<2 years) as compared to older children and adults (≥2 years). These results differ from those reported by Judd et al. (38) who observed substantially higher serotype richness in children <2 years of age for the USA as a whole. No previous analysis was available to compare Florida rarefaction results with national data.

The dominant serotypes circulating in Florida were strikingly different from those in the US as a whole. *S*. Javiana has previously been identified as a “Southern” serotype (39), e.g., in the CDC *Salmonella* atlas, which covers data from 1968 to 2011. The high incidence of *S*. Sandiego was unexpected. Previous data suggest this serotype occurred in low numbers throughout the USA between 1968 and 2011, without a clear geographic pattern. *S*. Sandiego has been involved in several outbreaks in the past decade. In 2013, eight outbreaks associated with pet turtles sickened 473 persons across the country. Multiple serotypes were involved, including two outbreaks with *S*. Sandiego (124 and 7 cases, respectively with *S*. Newport also involved in the larger outbreak). None of the *S*. Sandiego cases were from Florida (40). In 2018, an outbreak involving 101 cases was reportedly caused by Spring Pasta Salad purchased at Hy-Vee grocery stores. All cases resided in, or traveled to, states where these stores are located, i.e., Iowa, Kansas, Minnesota, Nebraska, and South Dakota. No cases we recognized in Florida (41). We detected a small cluster of three isolates of *S*. Sandiego from aquatic sources in Florida, but no human isolates were included in this cluster (Table 4). Further, studies are recommended to elucidate the reservoirs and transmission pathways of this serotype.

As previously observed (14), there was a high level of correspondence between eBurst Group assignments and serotype designations. Due to the dominance of a limited number of sequence types within an eBG, legacy MLST was of limited use for differentiating clinical *Salmonella* isolates beyond the serotype level, and thus, for identification of putative sources of infection. We therefore explored the use of core genome MLST as an alternative for epidemiological inference, which has also been reported by others as an effective method for surveillance purposes (42).

Our analysis suggests that hierarchical clustering of cgMLST profiles can be used as an effective screening to initiate more detailed phylogenetic analysis and is an efficient way to recognize potential outbreaks or prolonged case clusters in a large set of WGS data, as has been recognized as efficient approach in earlier studies (43). Nevertheless, comparison of results with PFGE surveillance was complex. WGS-based surveillance would have detected more putative outbreaks than PFGE surveillance, even though some outbreaks detected by PFGE surveillance would have been missed. Importantly, HC5 clustering detected additional clusters of clinical isolates that differed by isolation dates as much as 558 days. This suggests that there are persistent sources of *Salmonella* infection, that would not be detected by traditional outbreak detection, where a time window of 60 days is used to detect closely related isolates.

To investigate this hypothesis further, 14 clusters of clinical cases of *S*. Enteritidis with prolonged duration that were not related to detected outbreaks were investigated using SNP phylogenetic analysis. Eight of these clusters matched with non-clinical (all poultry) isolates from regulatory inspection by the United States Department of Agriculture Food Safety and Inspection Service (FSIS) through the GenomeTrakr Network. The phylogenetic results were supported by the presence of antimicrobial resistance genes in some clusters. Geospatial analysis suggested that these isolates were not only distant in time but also across locations throughout the whole US, supporting the hypothesis that some clones of *S*. Enteritidis persist in the broiler meat chain and sporadically cause human infections. Vertical transmission from parent stock or higher in the breeding pyramid is the most likely explanation of this widespread and sporadic occurrence of genetically similar isolates from poultry sources in different states and at different time points. Modern gene-based methods have recently been used to confirm vertical transmission in a vertically integrated poultry operation in Australia (44), and turkeys in the United States (45). Our study only included clinical isolates from Florida, and it would be interesting to explore if clinical isolates from other states can also be linked to these persistent clones. FSIS samples chicken carcasses at slaughter and finished products (chicken parts and ground chicken). Many establishments purchase carcasses for further processing into finished product, which obscures the origin of a common strain. Among 513 isolates in *S*. Enteritidis clusters, 30 were from animal-young chicken, 50 from chicken carcass, 144 from comminuted chicken, and 289 from raw intact chicken. No isolates from turkey were detected among these clusters.

Use of genomic diversity for assessing genetic and epidemiological linkages of pathogens has been widely used. SNP-based and gene-by-gene (MLST) methods (12) have gained more popularity, the choice being dependent on the intended application (46). Previous studies comparing the use of cgMLST and SNP-based phylogeny for surveillance purposes have suggested that cgMLST presents a reproducible and scalable approach for outbreak detection and is congruent with SNP analysis (43, 46–49), which uses one or several mutations across different alleles and results in intrinsically higher discriminatory power (50). Each approach has their strength and shortcomings. The gene-by-gene approach is helpful to collapse large genomic data sets into a compact and simpler form but might miss individual nucleotide variants within alleles. SNP-based approaches have extensively been used for outbreak detection (47, 51, 52) and provide great distinguishing power, differentiating isolates even at the level of single base pair substitutions (in theory). However, they often are computationally demanding and time consuming, and are sensitive to recombination, contamination and sequence coverage (52). Both these approaches further need epidemiological data support before confirming any outbreak or prolonged case series (53). In the current work, we have proposed a “combination approach” to utilize the merits of both cgMLST (compactness) and SNP- based approaches (high discriminatory power). We suggest the use of HierCC (54) method for screening genetically closer isolates, reducing the sample size considerably for SNP-based phylogeny. We found that Hierarchical Clustering at five or less allelic difference can be used as an efficient tradeoff between inclusiveness and specificity. As genetic diversity varies by *Salmonella* serotype, more experience with this method is needed and outbreak investigations should include building SNP-based phylogenies to support conclusions about genetic relatedness of isolates and possibly adapt the level of hierarchical clustering to address the specific research question or serotype. Such work may also support establishment of serotype-specific working thresholds for outbreak detection. Hierarchical clustering also appears to be an effective method to identify case clusters that are more distant in time and place than traditional outbreaks but may have been infected from a common source. The potential of this new approach to source identification would be strengthened by more systematic surveillance of *Salmonella* in non-human reservoirs. Current data for Florida is very limited. Isolates in NCBI were mainly deposited by FSIS, thereby biasing the available data to animal source foods. Isolates from other foods and non-clinical sources from Florida are very rare in the public domain and need more investigation.

## Data Availability Statement

The datasets presented in this study can be found in online repositories. The names of the repository/repositories and accession number(s) can be found in the article/Supplementary Material.

## Ethics Statement

The studies involving human participants were reviewed and approved by University of Florida Institutional Review Board. Written informed consent from the participants' legal guardian/next of kin was not required to participate in this study in accordance with the national legislation and the institutional requirements.

## Author Contributions

NS, XL, and AH conceived the study, performed the analyses, and drafted the manuscript. JB supervised the laboratory analysis. EB and JD performed the surveillance metadata collection and contributed to the writing. All authors critically revised the manuscript and gave final approval of the version to be published.

## Conflict of Interest

The authors declare that the research was conducted in the absence of any commercial or financial relationships that could be construed as a potential conflict of interest.
