# Supplementary material for: Molecular Epidemiology of Salmonellosis in Florida, USA, 2017–2018
Source: Front Med (Lausanne). 2021 Apr 22;8:656827. doi: 10.3389/fmed.2021.656827 (PMC8100233; doi:10.3389/fmed.2021.656827)
Supplement: Supplementary file 1 [file Data_Sheet_1.PDF]

Supplementary Figures

Supplementary Figure S1: Hierarchical clustering (HC) of 1,632 sporadic clinical isolates collected in Florida during 2017-18, nodes are colored by HC clusters for corresponding HC level , as shown in S1.1-4 for HC20, HC10, HC5 and HC2 respectively

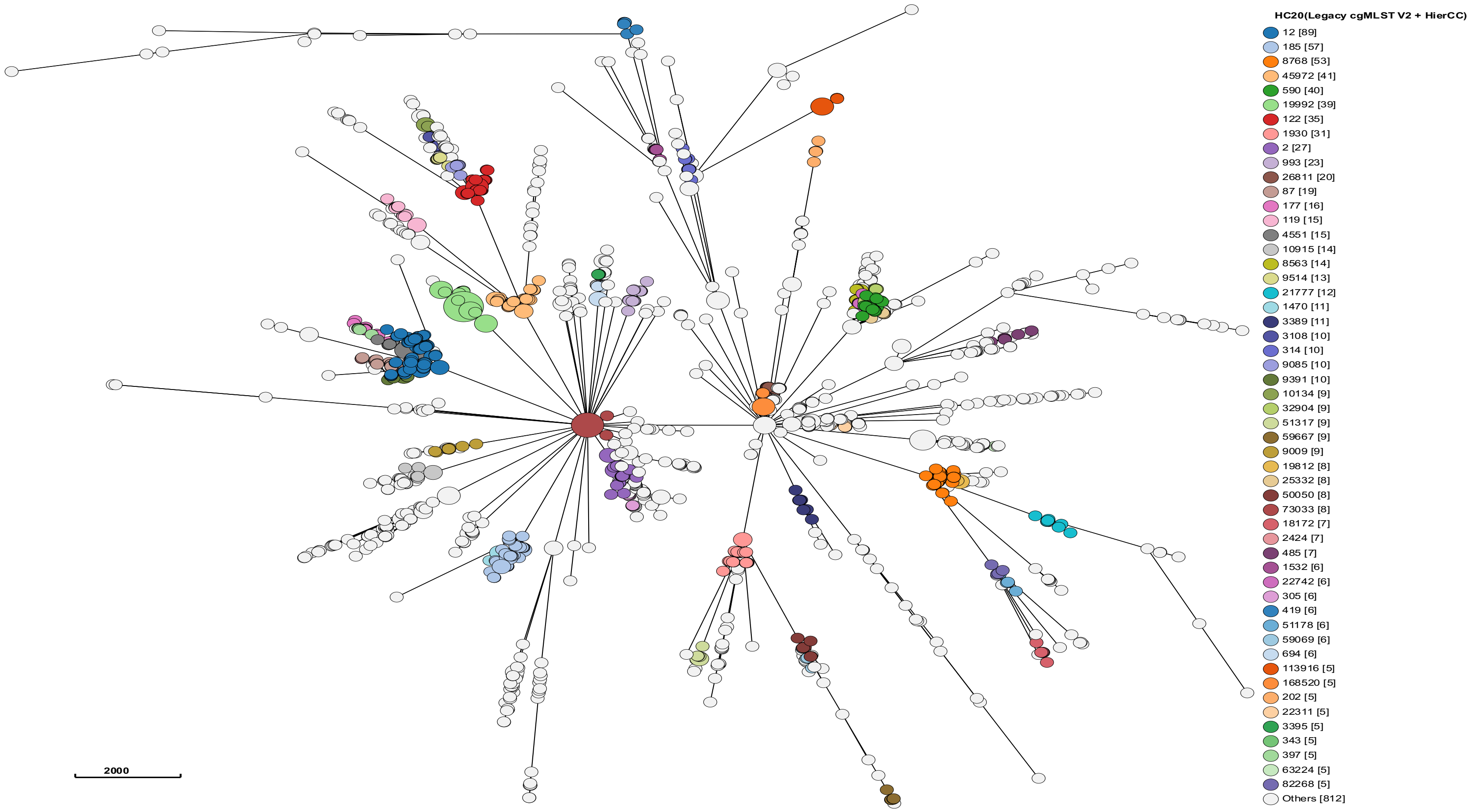

S1.1: Hierarchical clustering at HC20

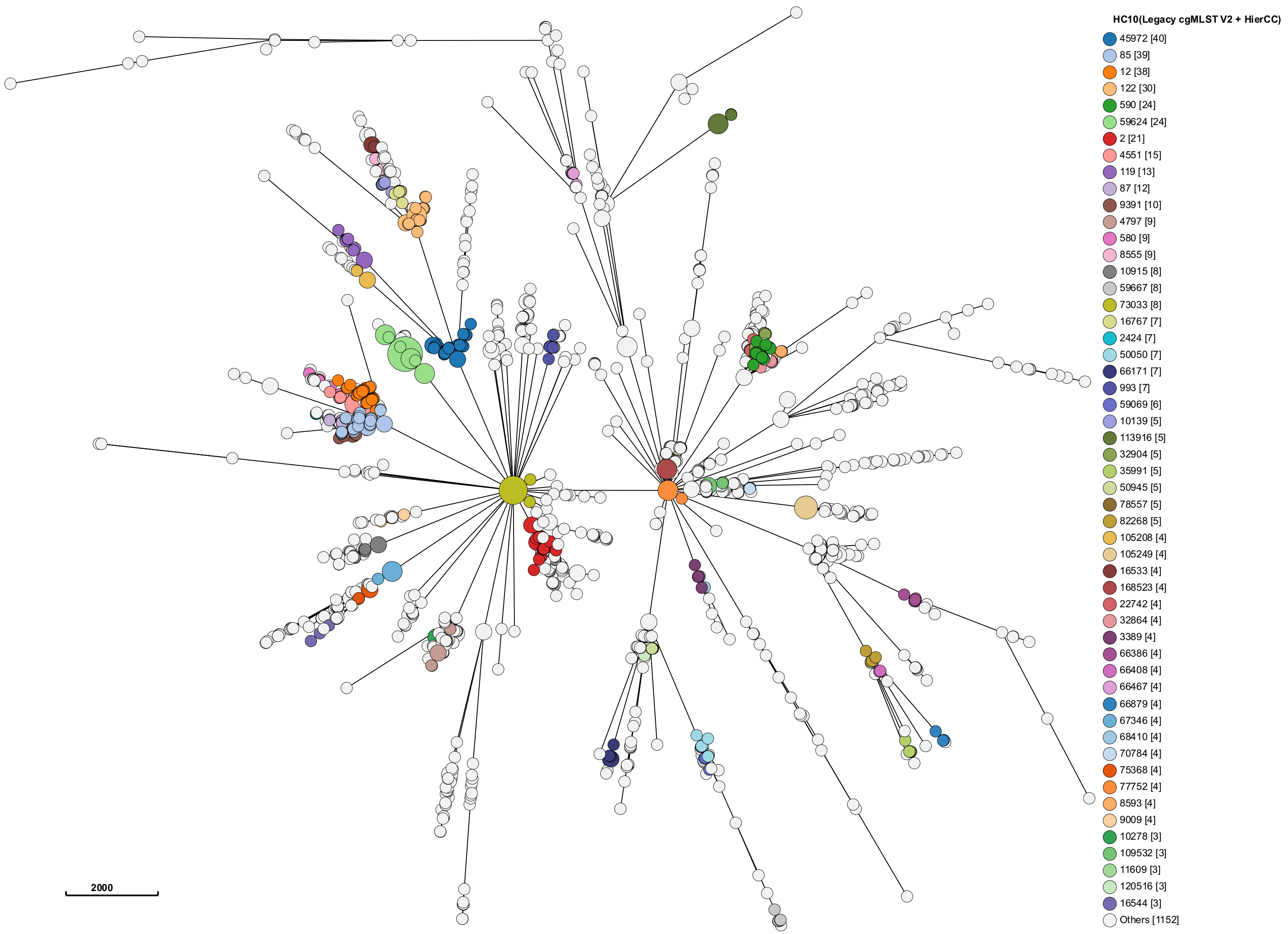

S1.2: Hierarchical clustering at HC10

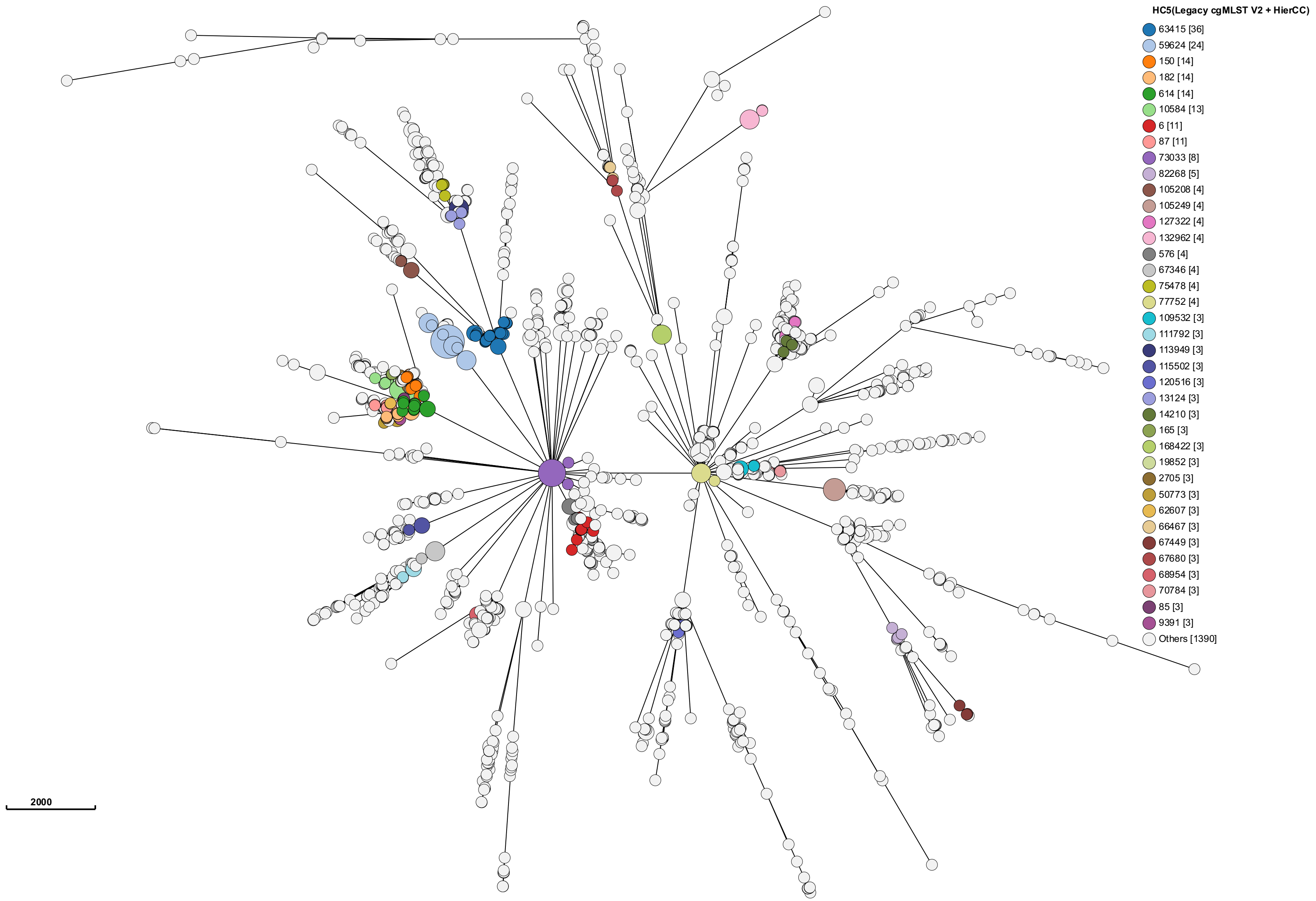

S1.3: Hierarchical clustering at HC5

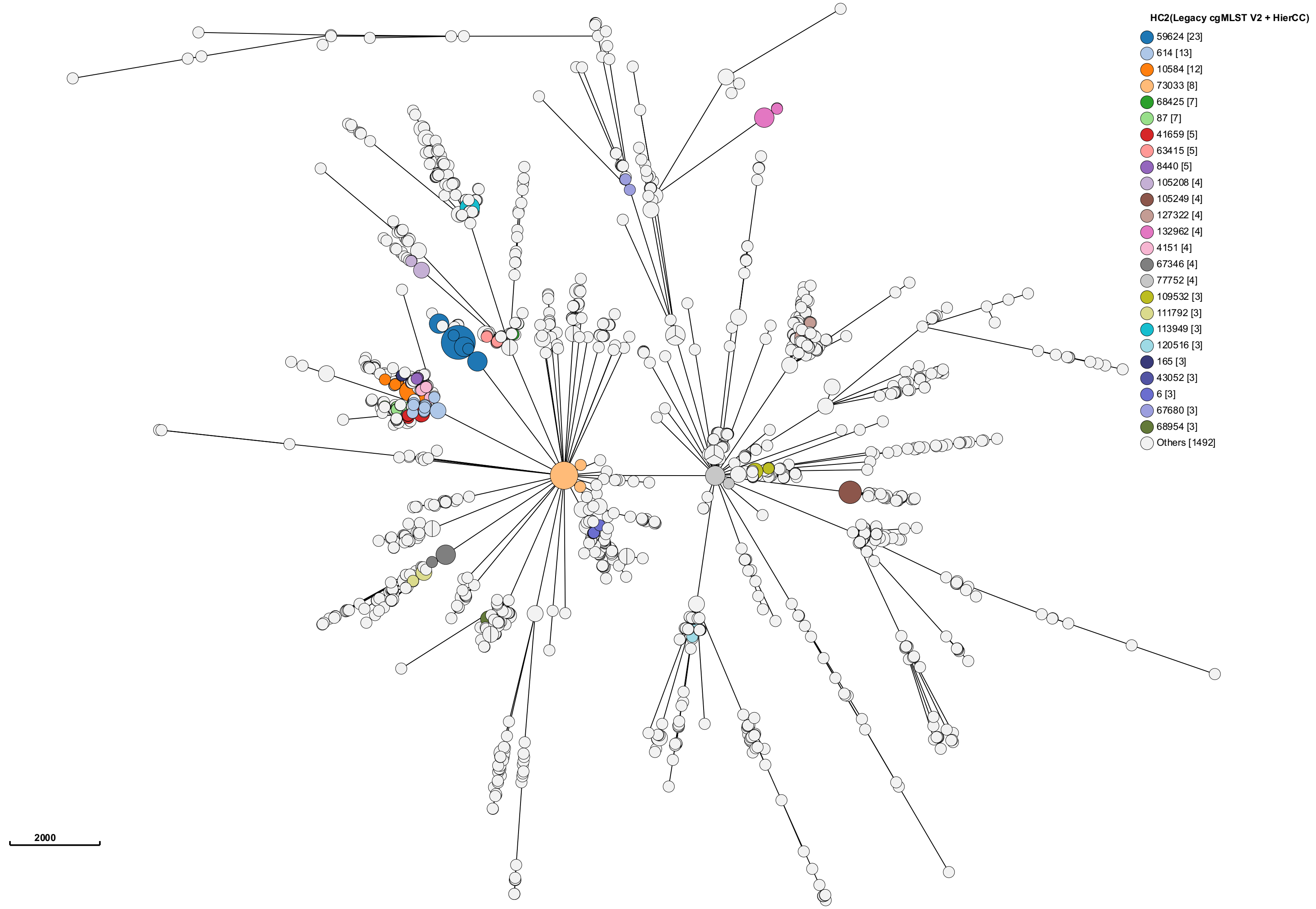

S1.4: Hierarchical clustering at HC20

Supplementary Figure S2:

Heatmaps of **S. Enteritidis** drawn using pairwise SNP distance for HC5 clusters (>2 isolates) are shown in S2.1-14:

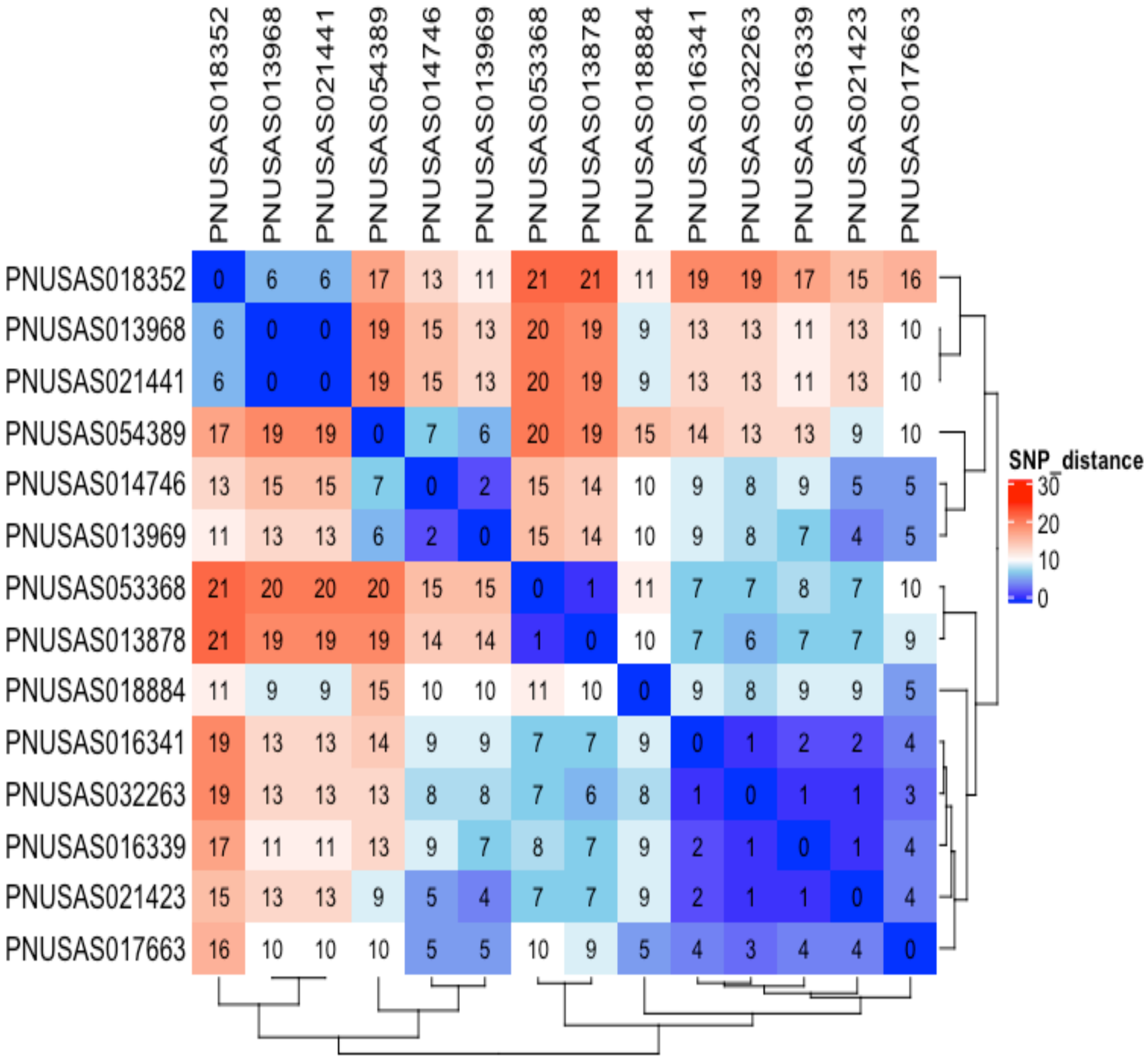

S2.1. SNP-distance Heatmap of HC5 150

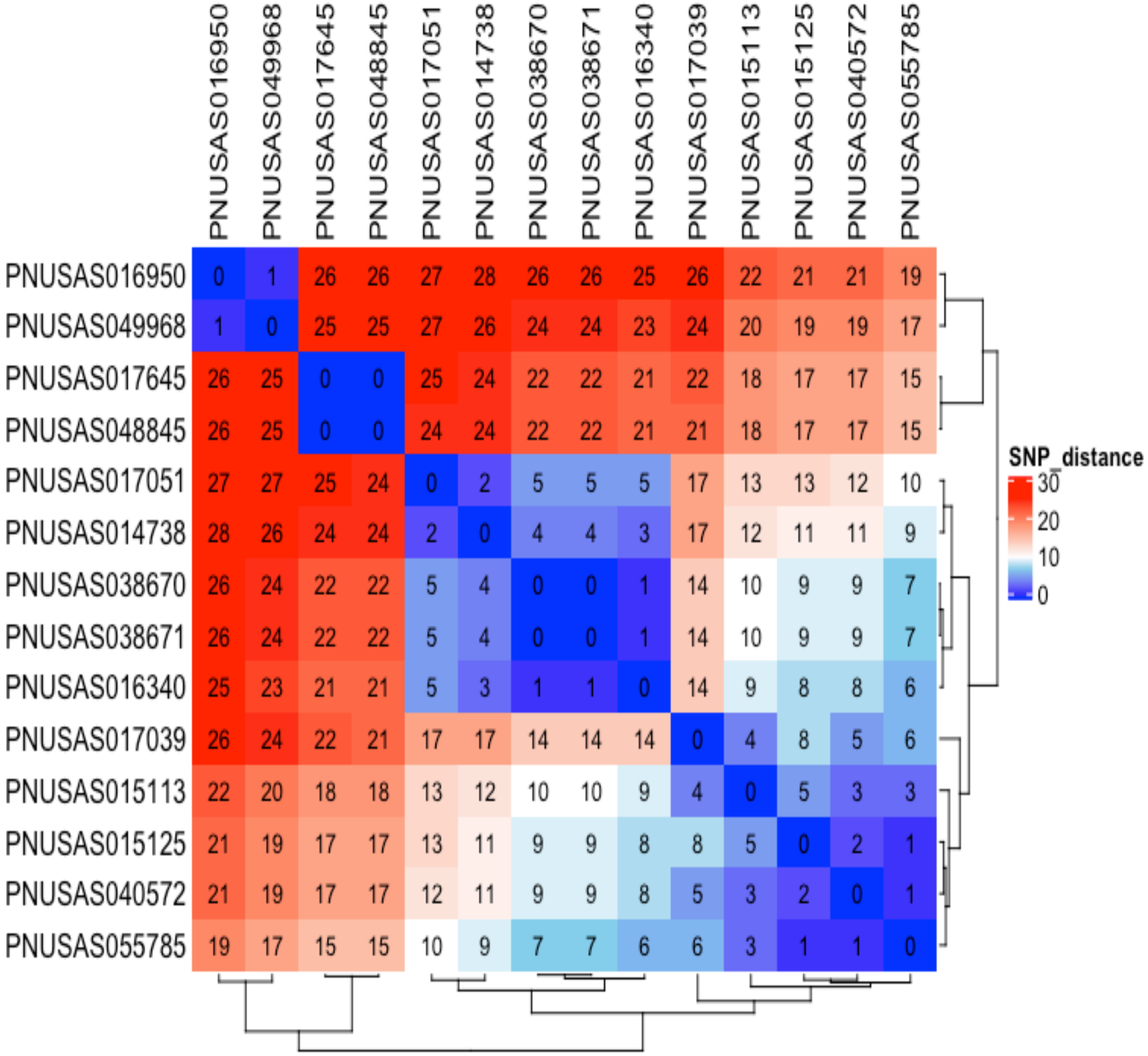

S2.2. SNP-distance Heatmap of HC5 182

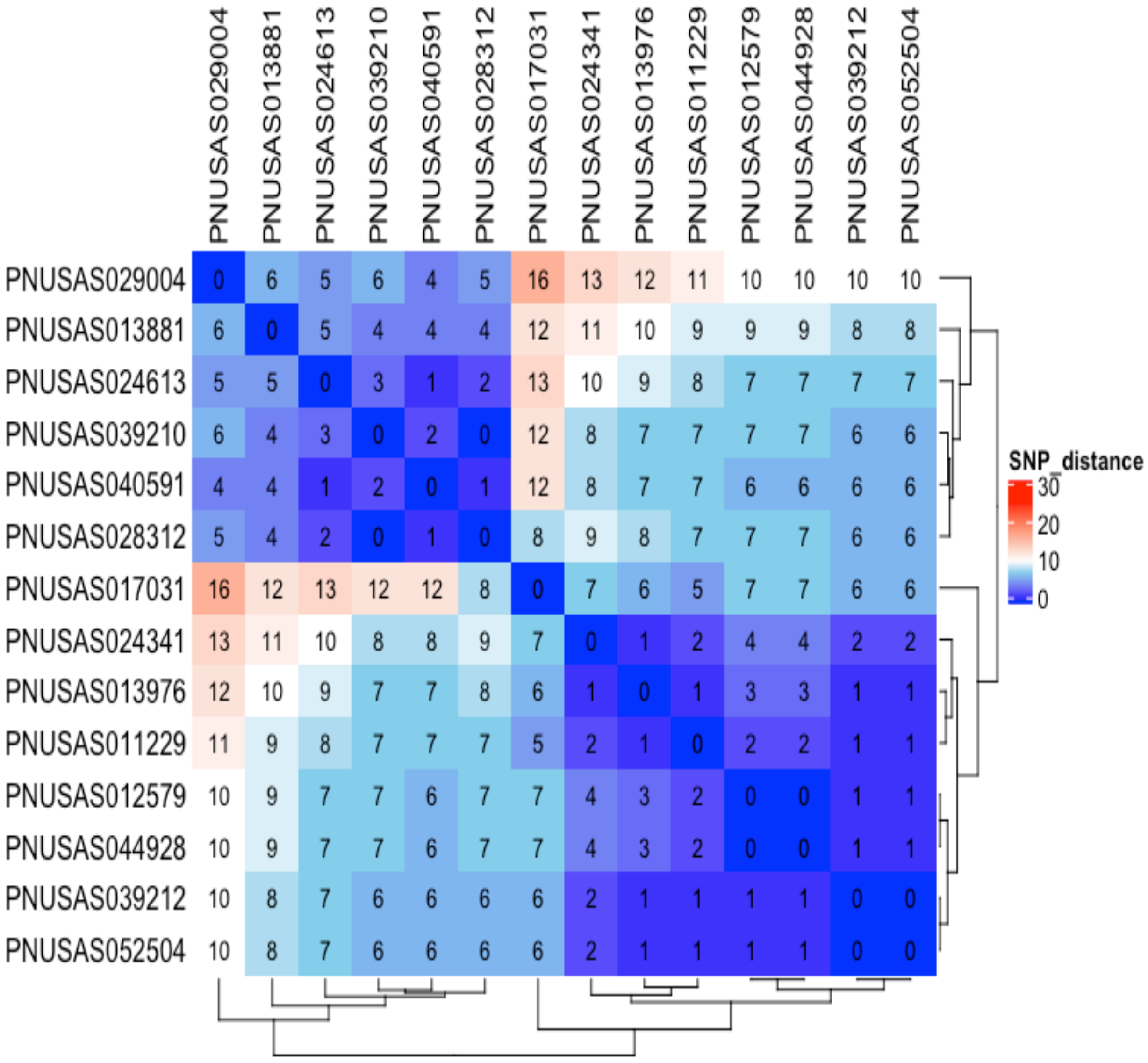

S2.3. SNP-distance Heatmap of HC5 614

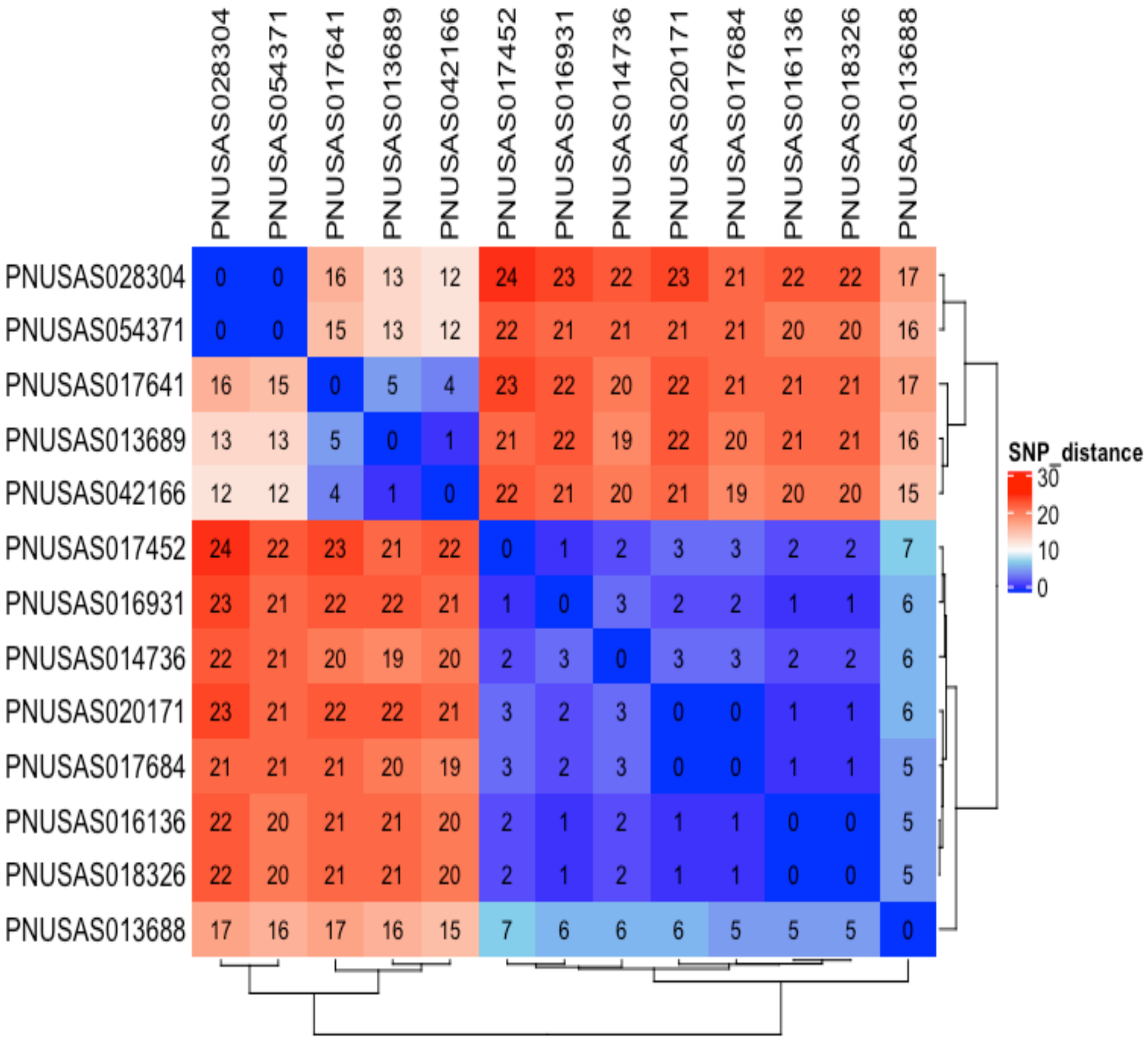

S2.4. SNP-distance Heatmap of HC5 87

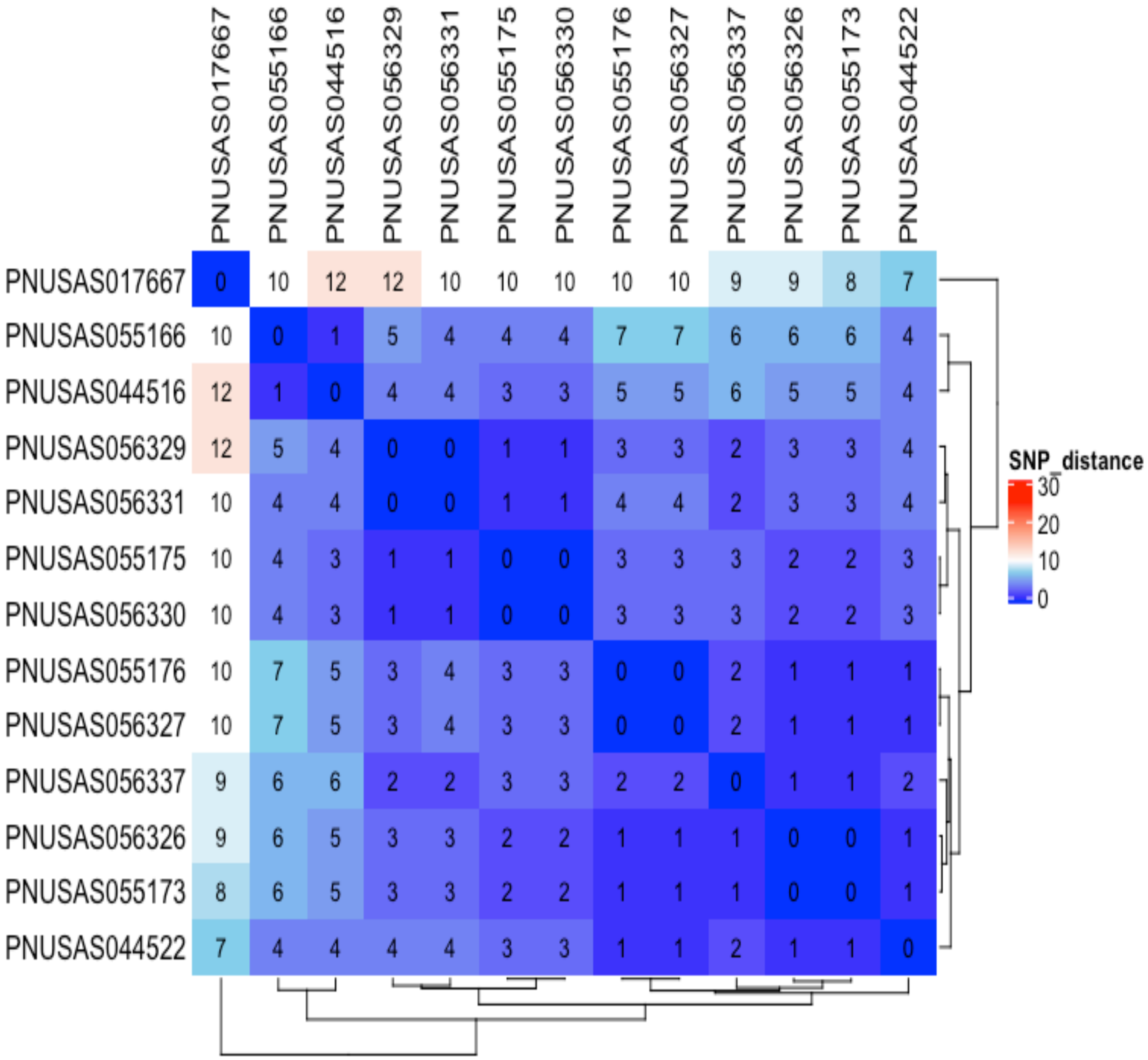

S2.5. SNP-distance Heatmap of HC5 10584

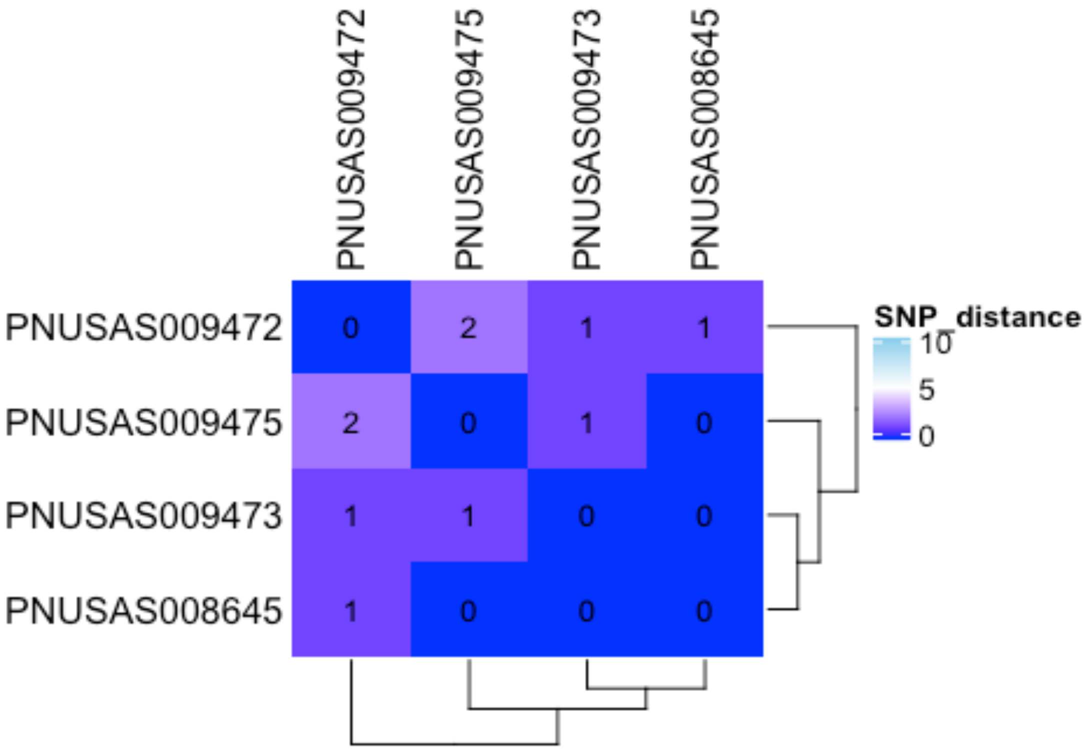

S1.6. SNP-distance Heatmap of HC5 58778

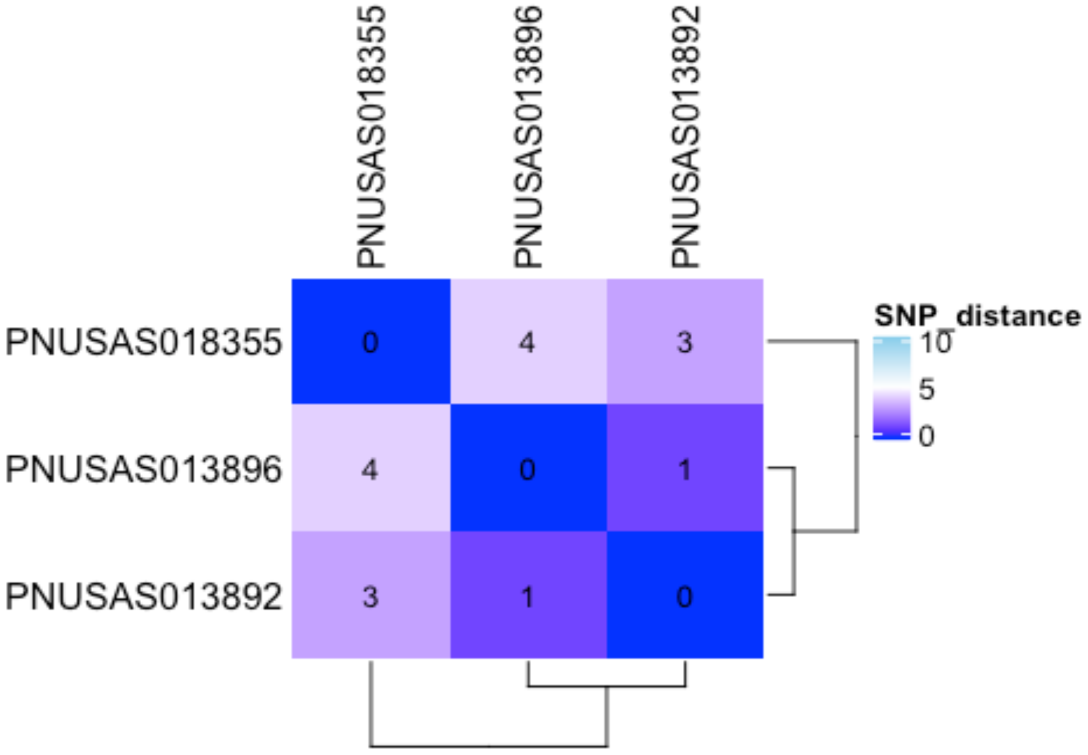

S2.7. SNP-distance Heatmap of HC5 85

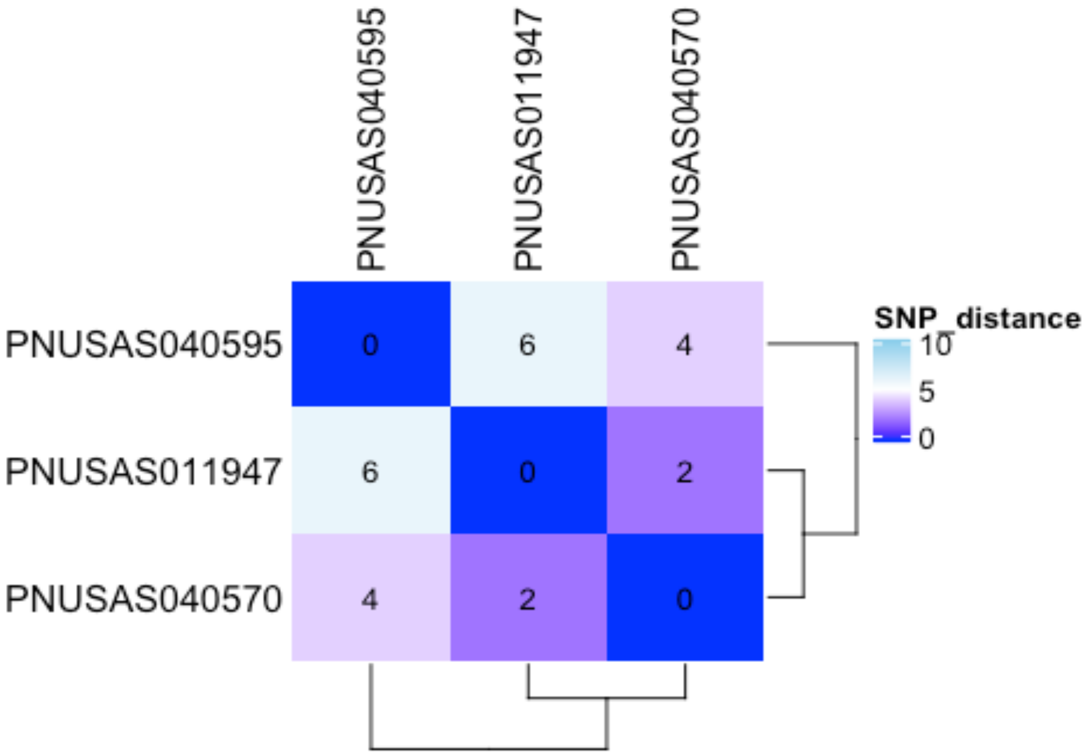

S2.8. SNP-distance Heatmap of HC5 165

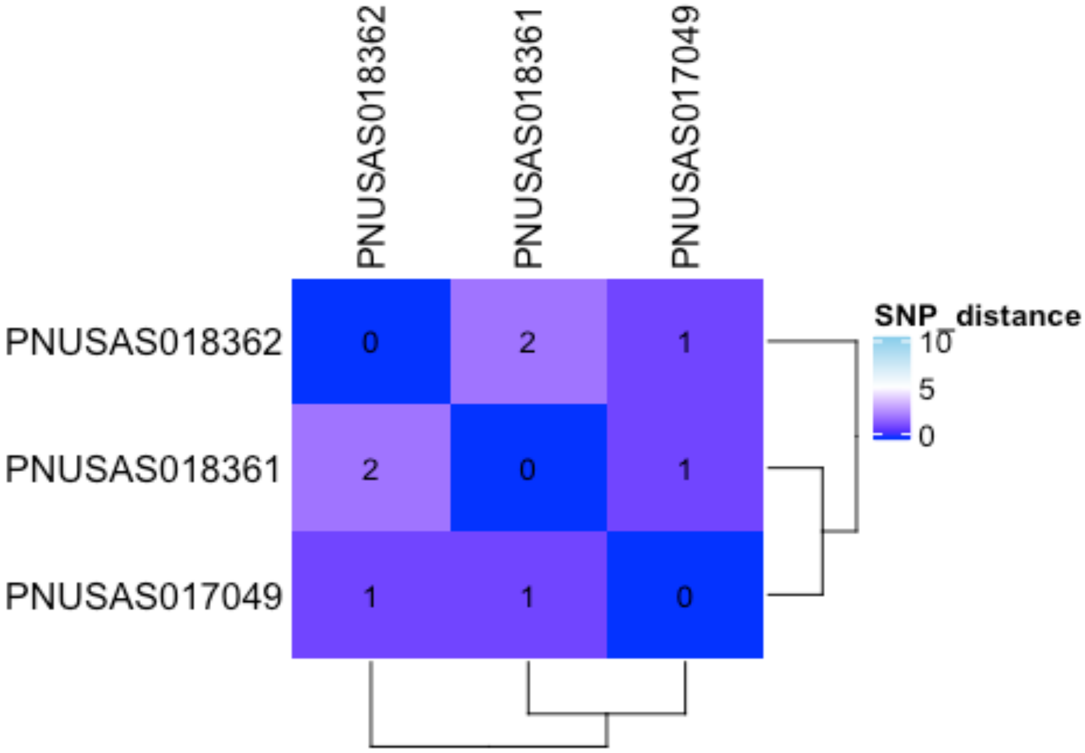

S2.9. SNP-distance Heatmap of HC5 2705

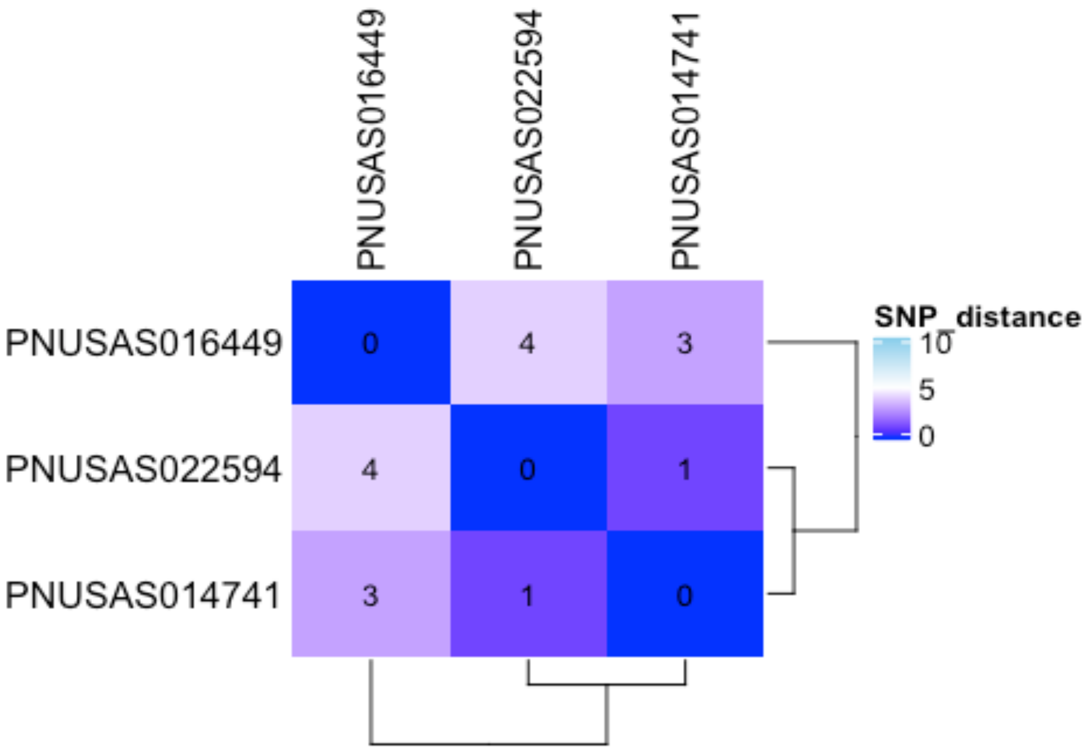

S2.10. SNP-distance Heatmap of HC5 9391

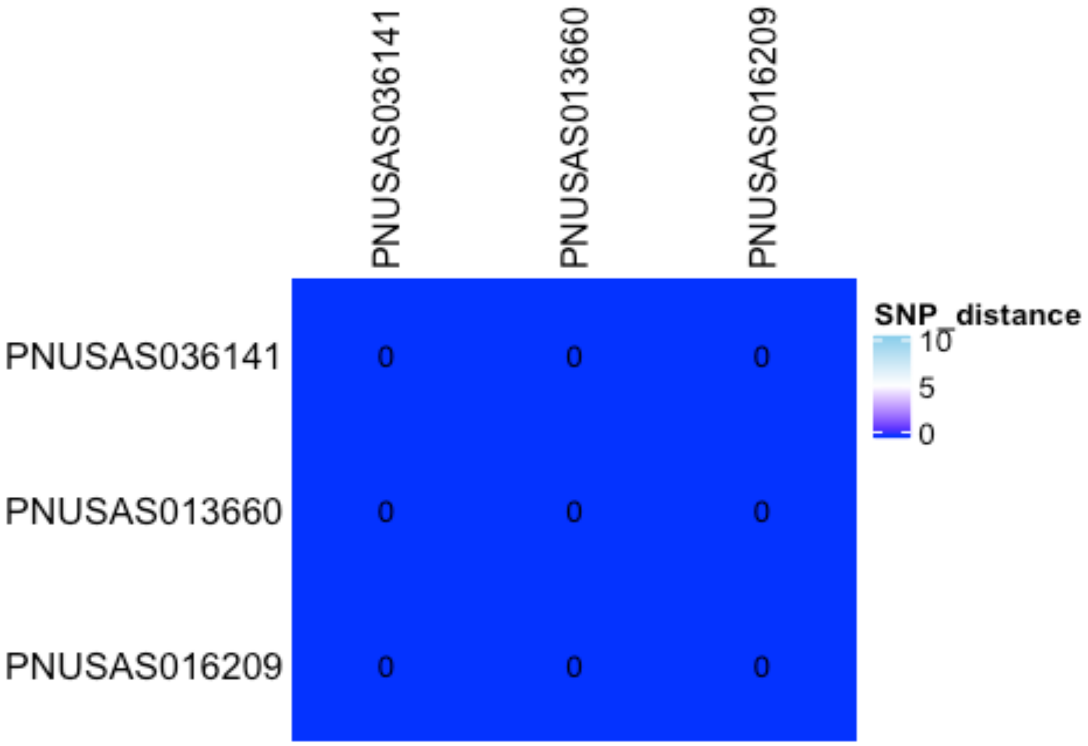

S2.11. SNP-distance Heatmap of HC5 19852

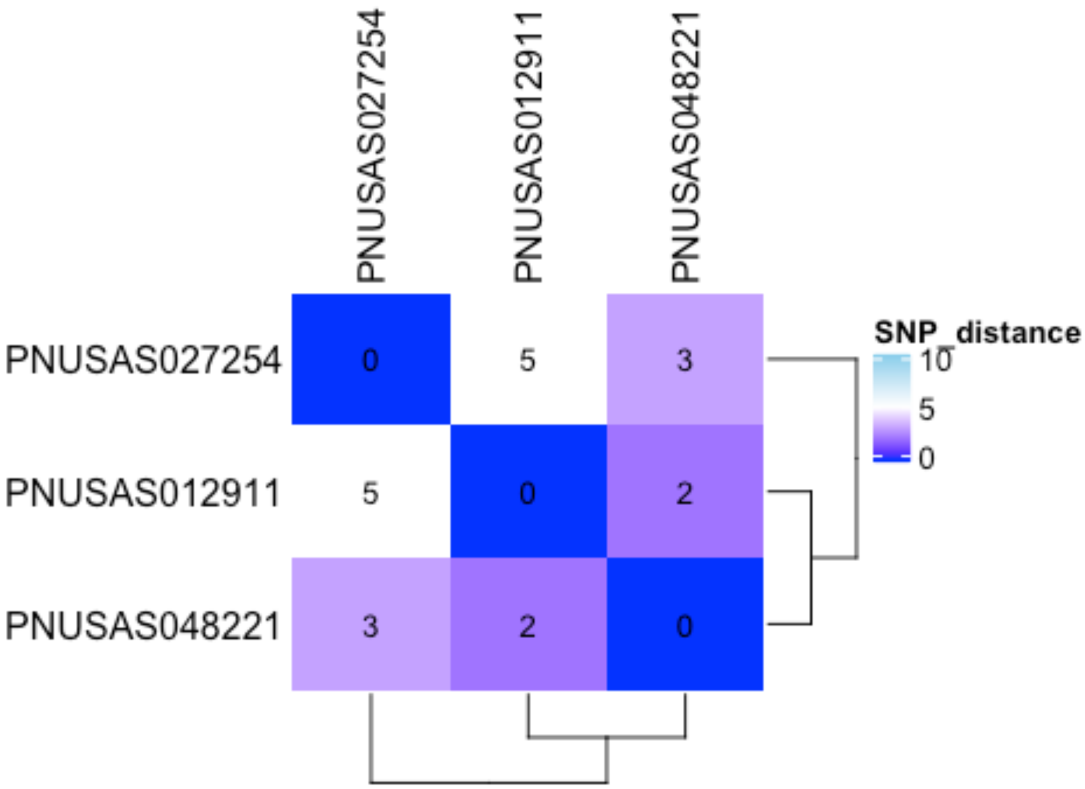

S2.12. SNP-distance Heatmap of HC5 26508

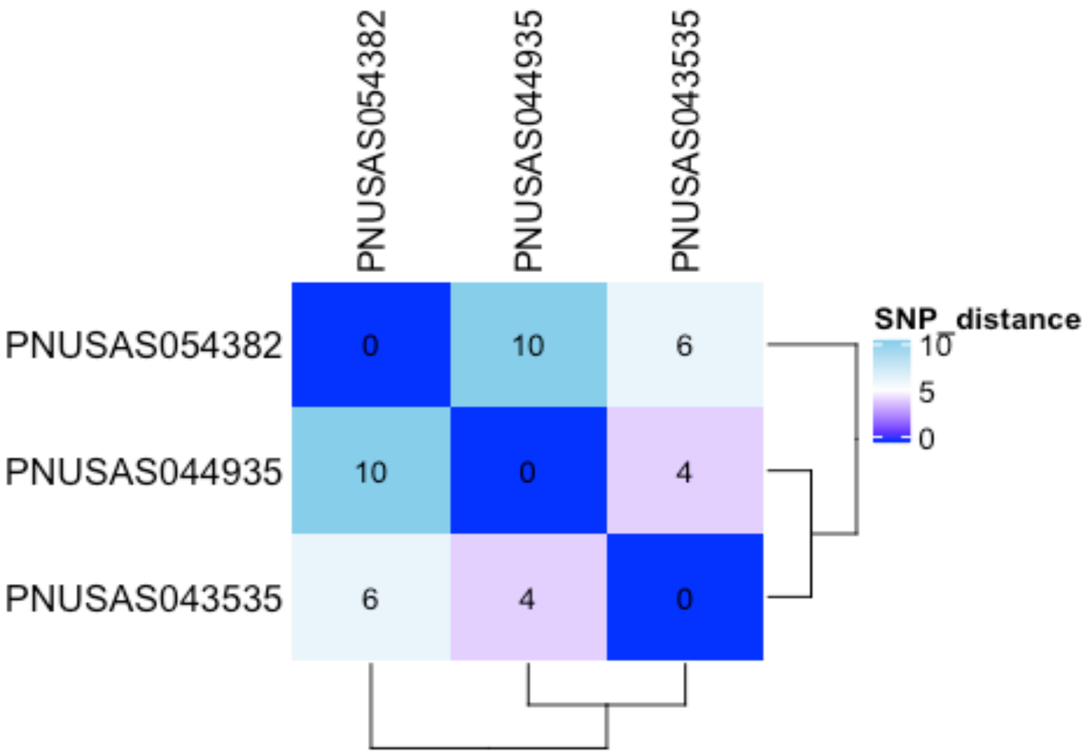

S2.13. SNP-distance Heatmap of HC5 50773

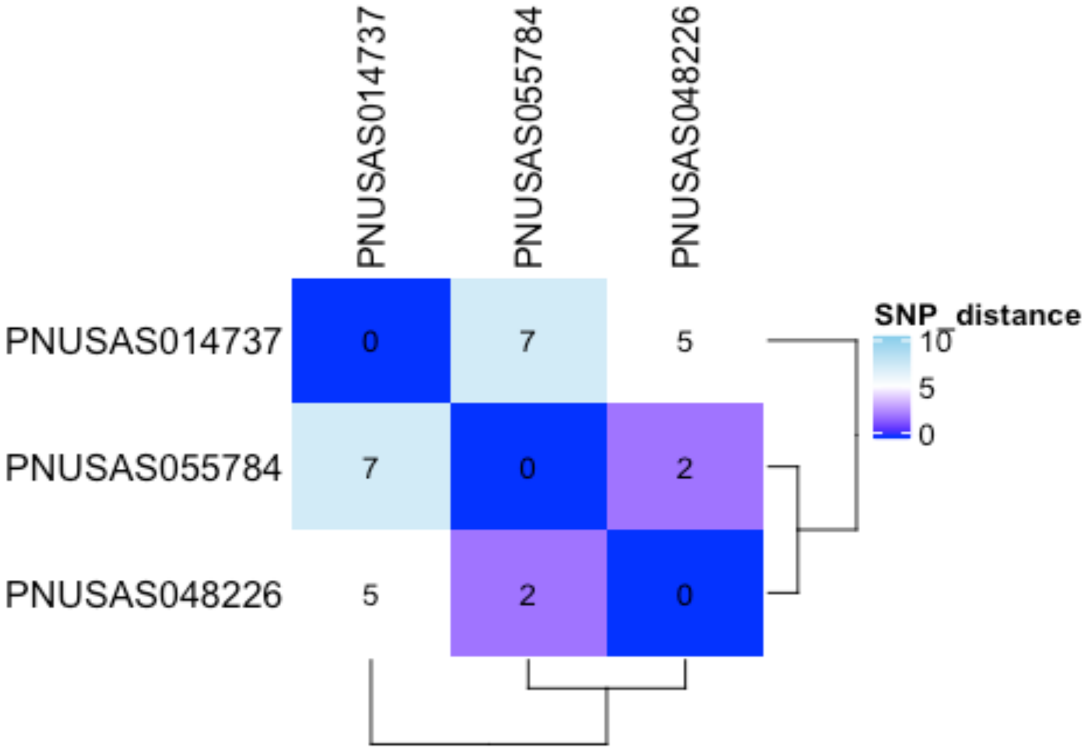

S2.14. SNP-distance Heatmap of HC5 62607

Supplementary Figure S3:

Heatmaps of **S. Newport** drawn using pairwise SNP distance for HC5 clusters (>2 isolates) are shown in S3.1-4:

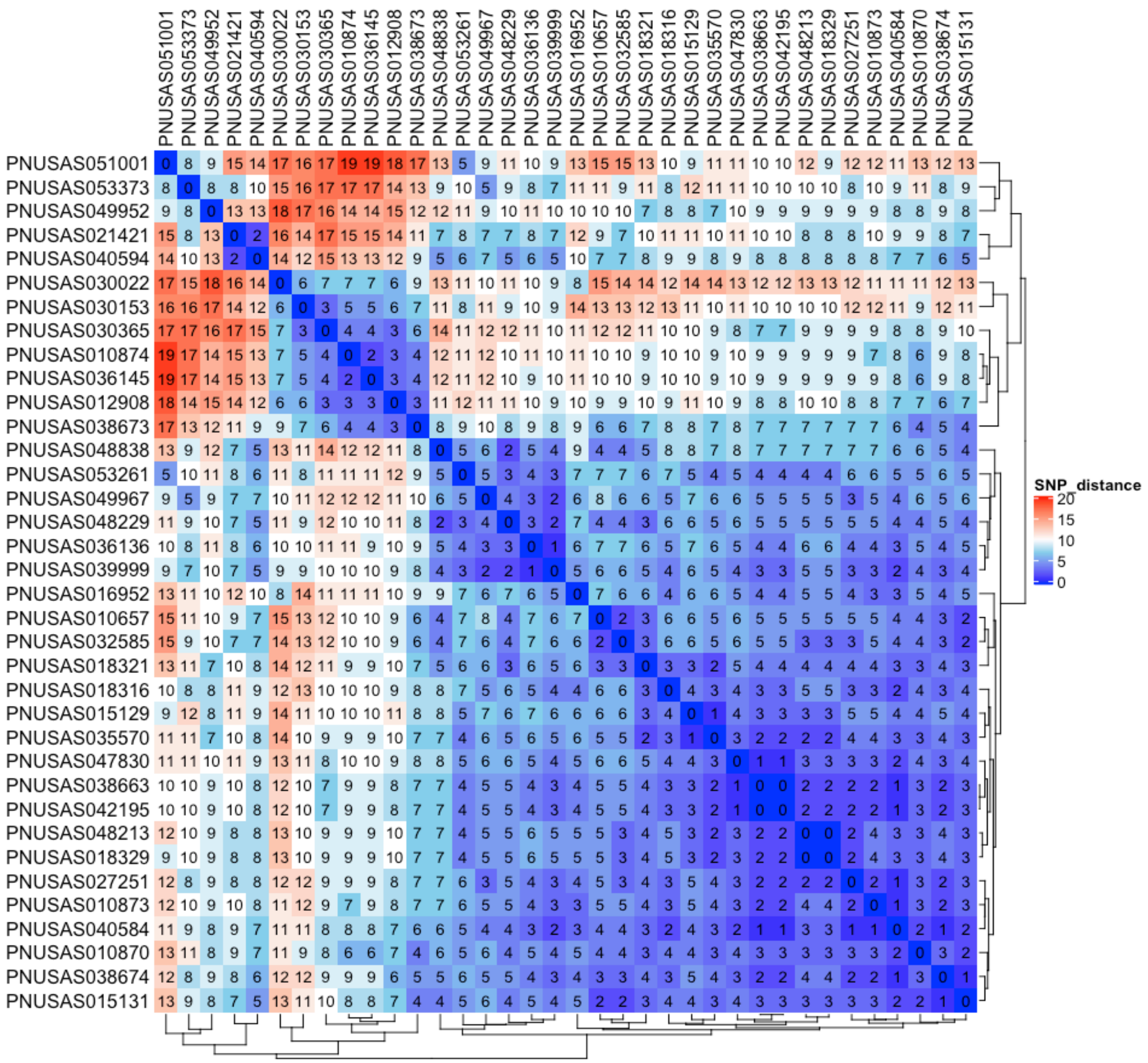

S3.1. SNP-distance Heatmap of HC5 63415

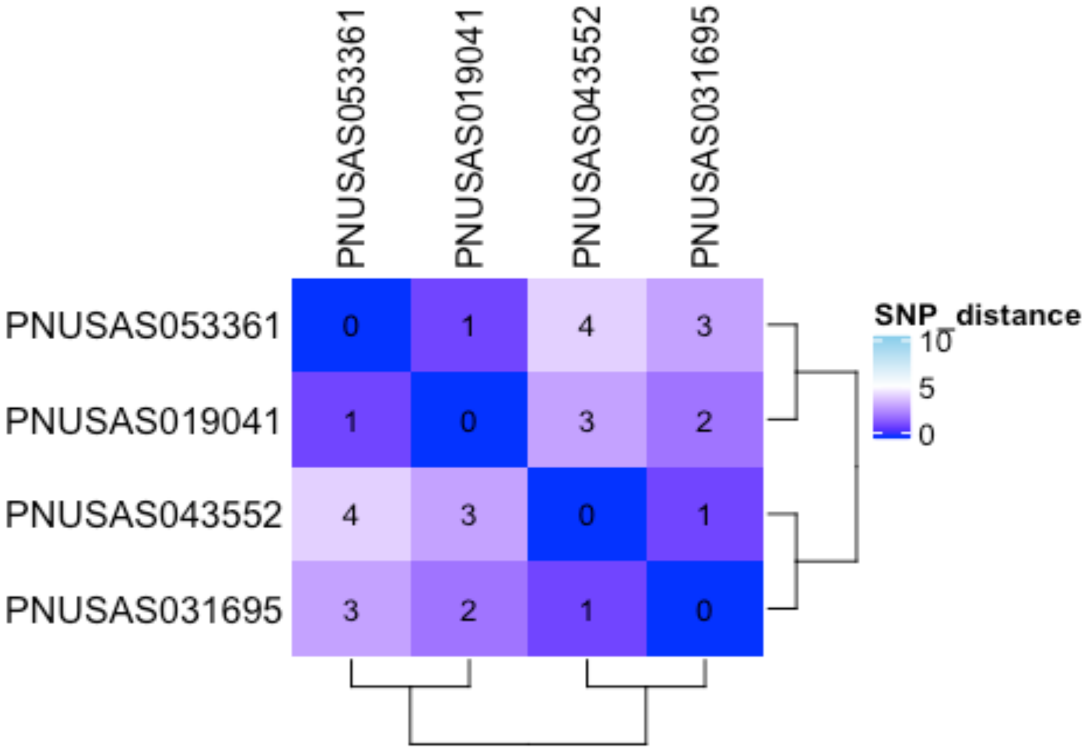

S3.2. SNP-distance Heatmap of HC5 75478

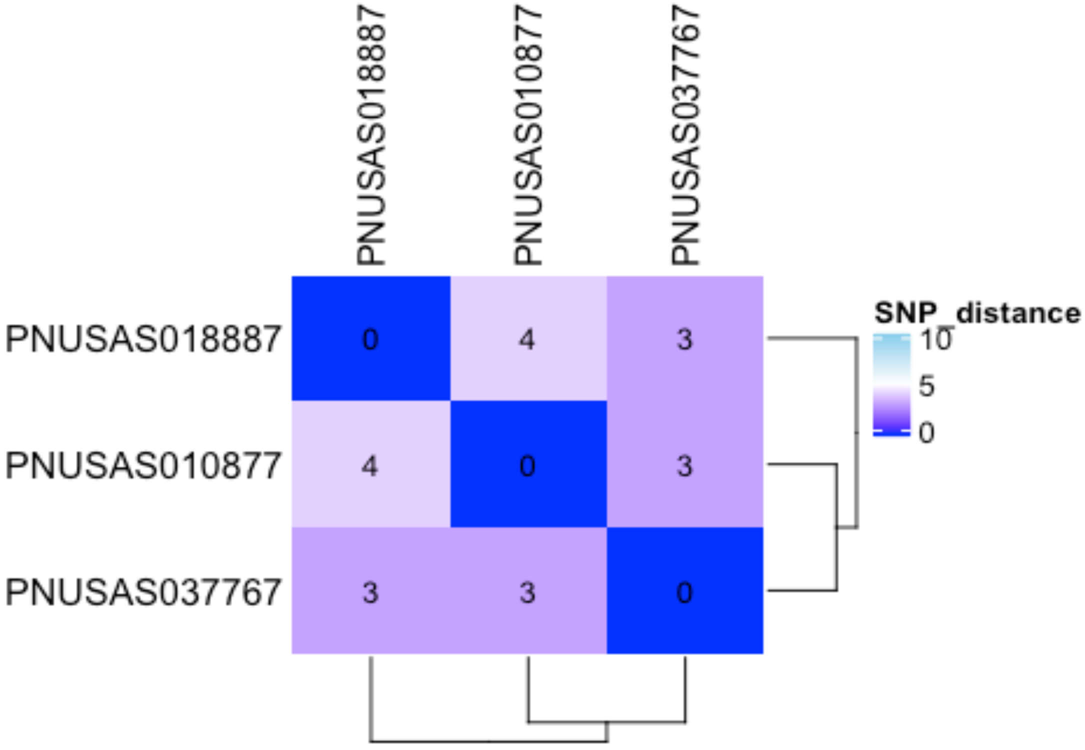

S3.3. SNP-distance Heatmap of HC5 13124

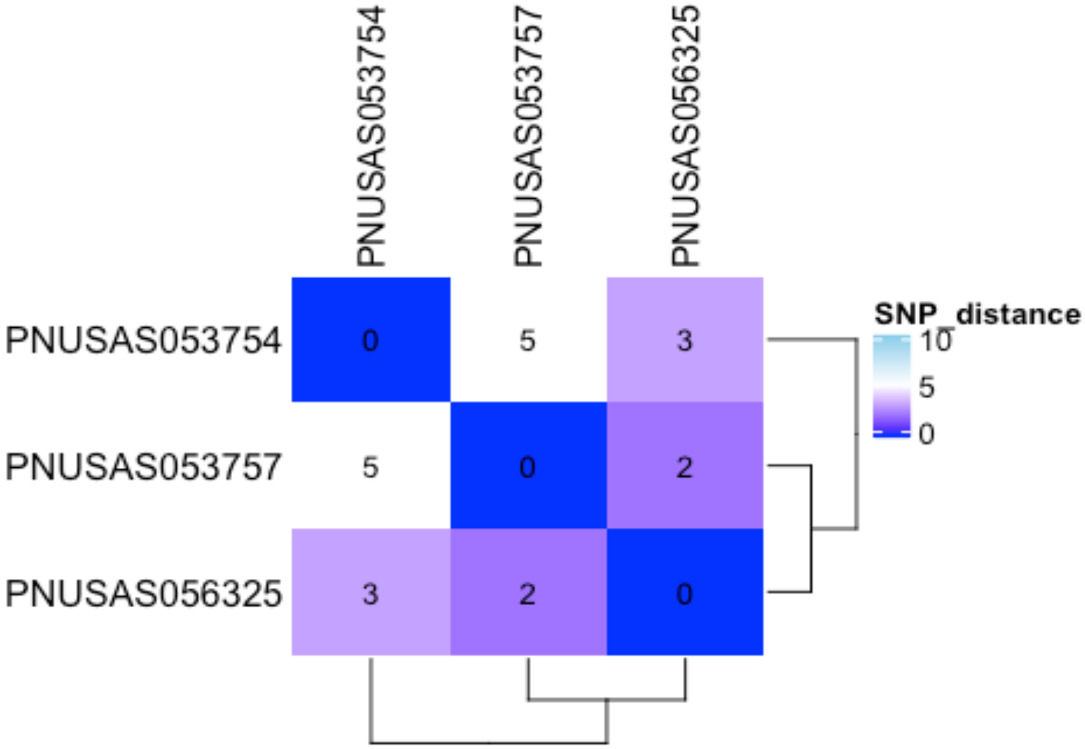

S3.4. SNP-distance Heatmap of HC5 47842

Supplementary Figure S4:

Heatmaps of **S. Javiana** drawn using pairwise SNP distance for HC5 clusters (>2 isolates) are shown in S4.1-2:

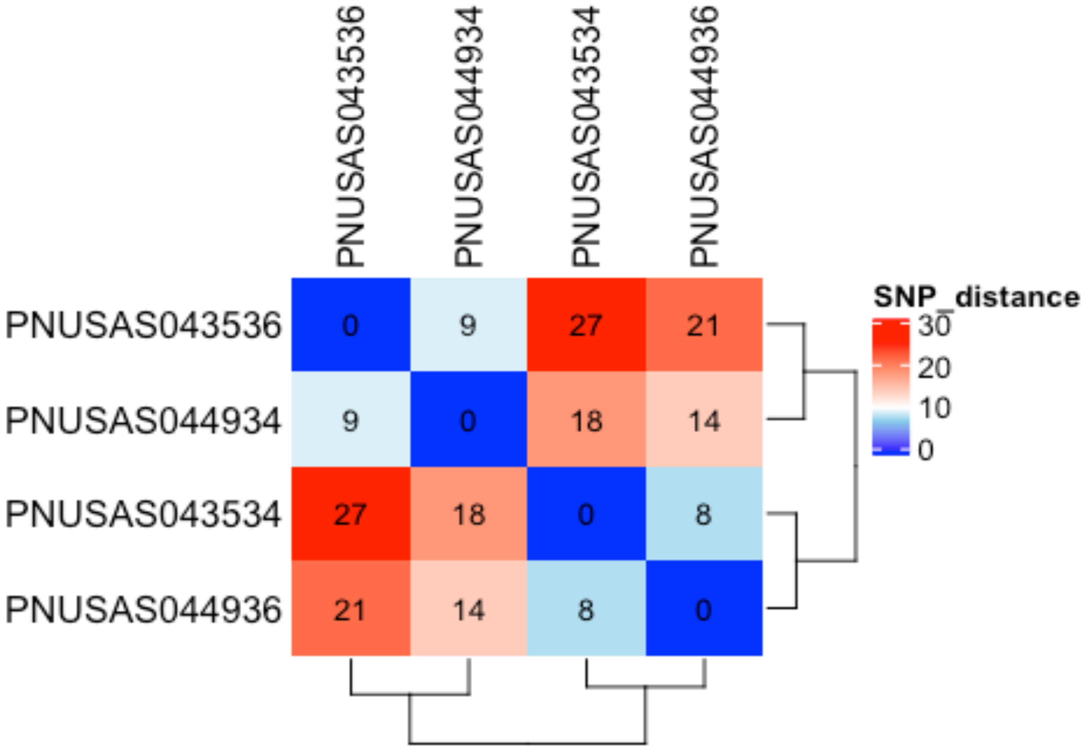

S4.1. SNP-distance Heatmap of HC5 127322

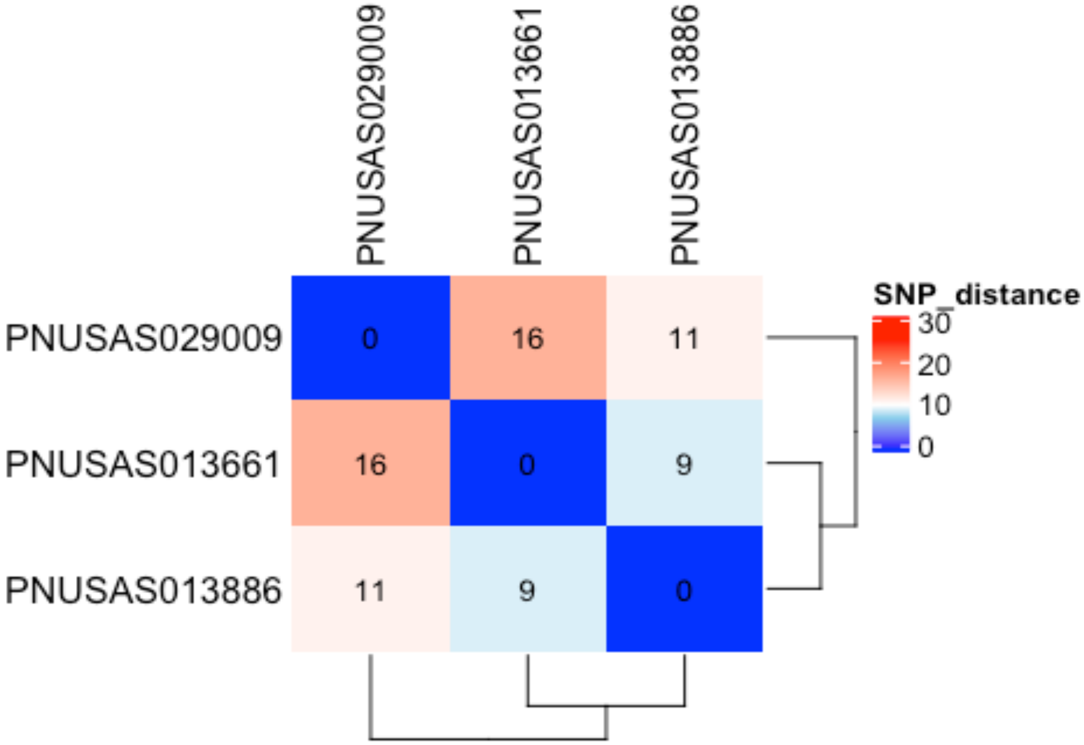

S4.2. SNP-distance Heatmap of HC5 14210

Supplementary Figure S5:

Heatmaps of **S. Bareilly** drawn using pairwise SNP distance for HC5 clusters (>2 isolates) are shown in S5.1-2:

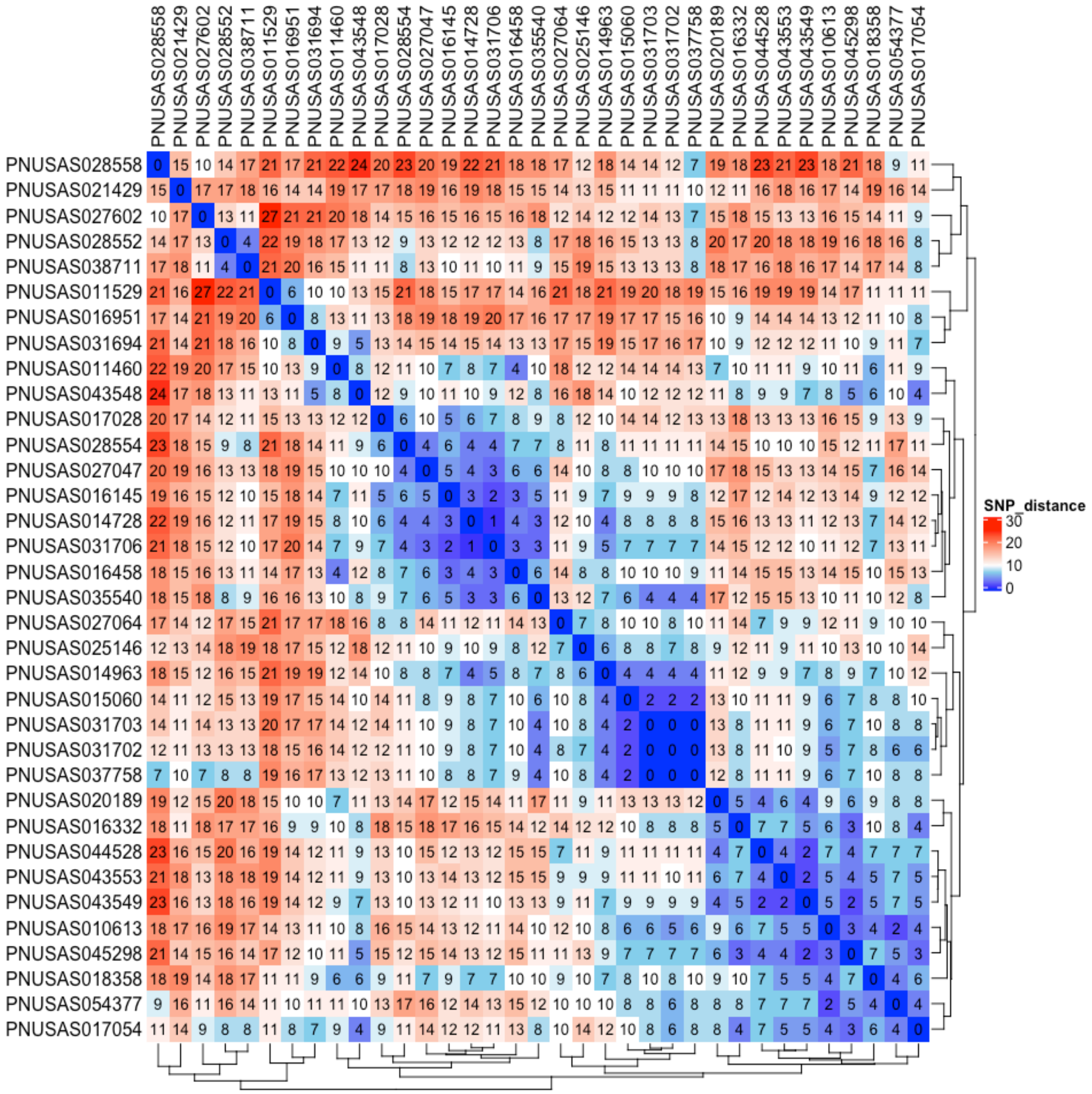

S5.1. SNP-distance Heatmap of HC5 59624

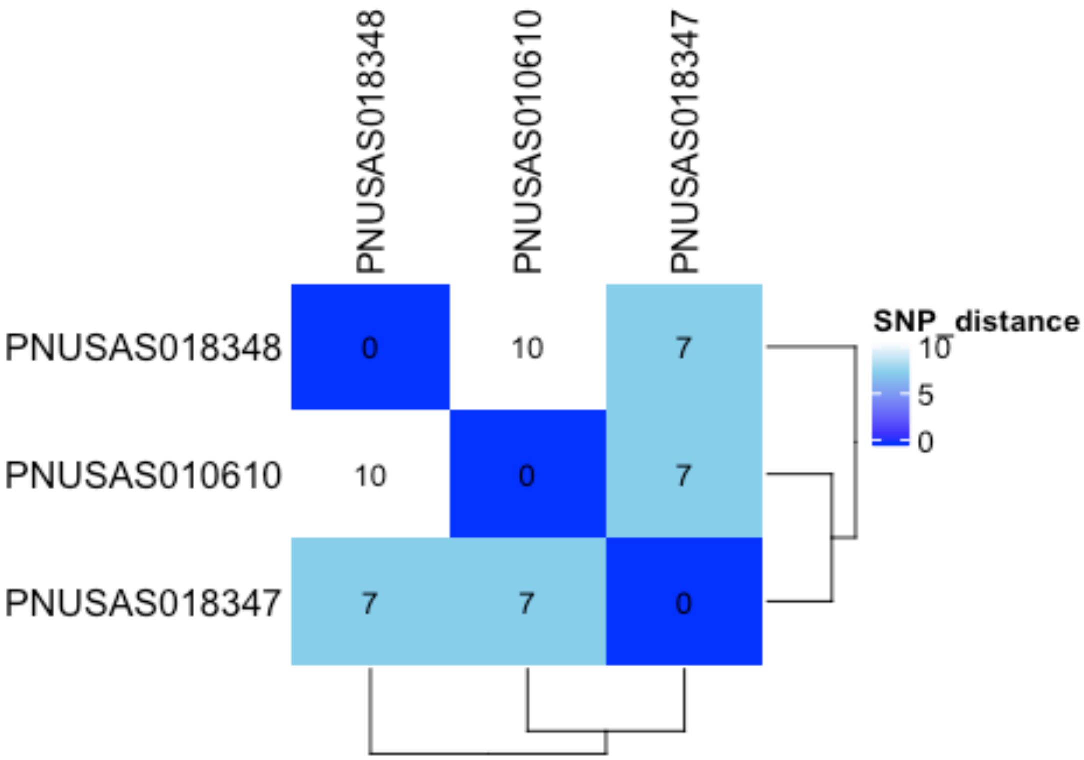

S5.2. SNP-distance Heatmap of HC5 67069

Supplementary Figure S6:

Heatmaps of **S. Typhimurium** and **I i4,[5],12:i-** drawn using pairwise SNP distance for HC5 clusters (>2 isolates) are shown in S6.1-3:

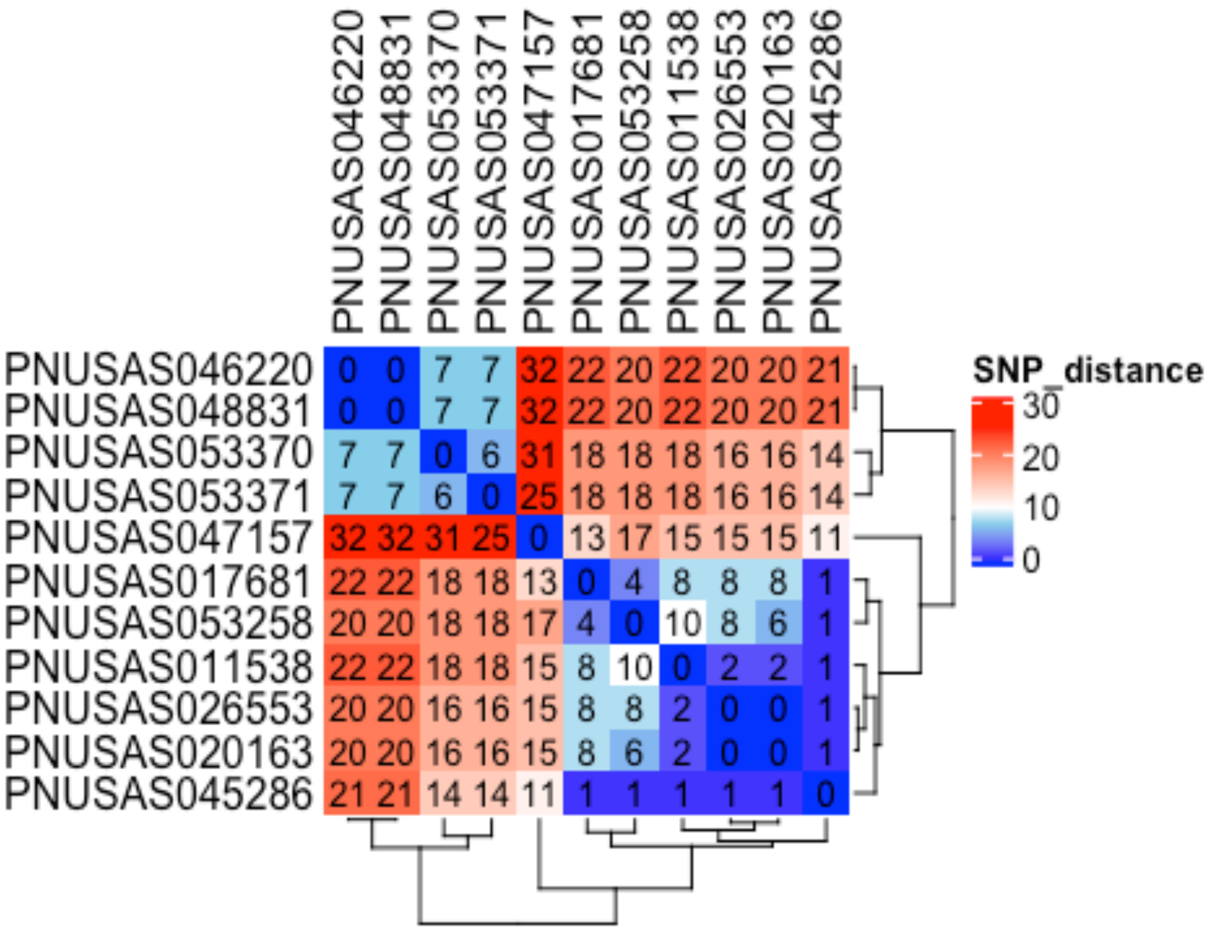

S6.1. SNP-distance Heatmap of HC5 6

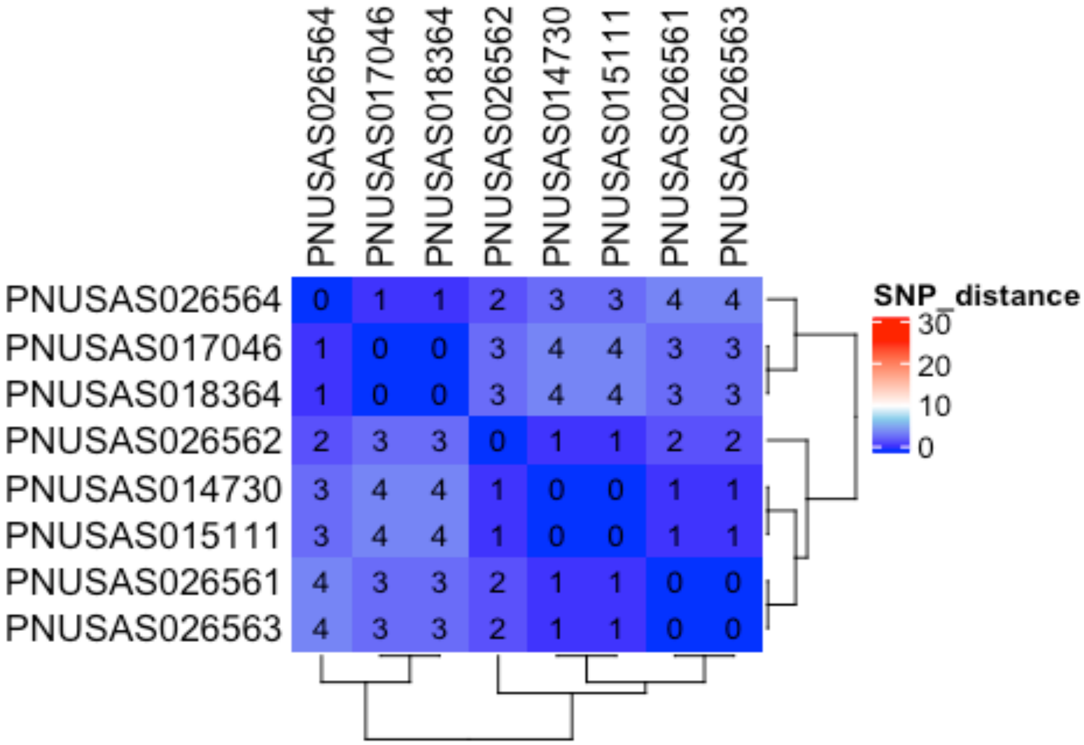

S6.2. SNP-distance Heatmap of HC5 73033

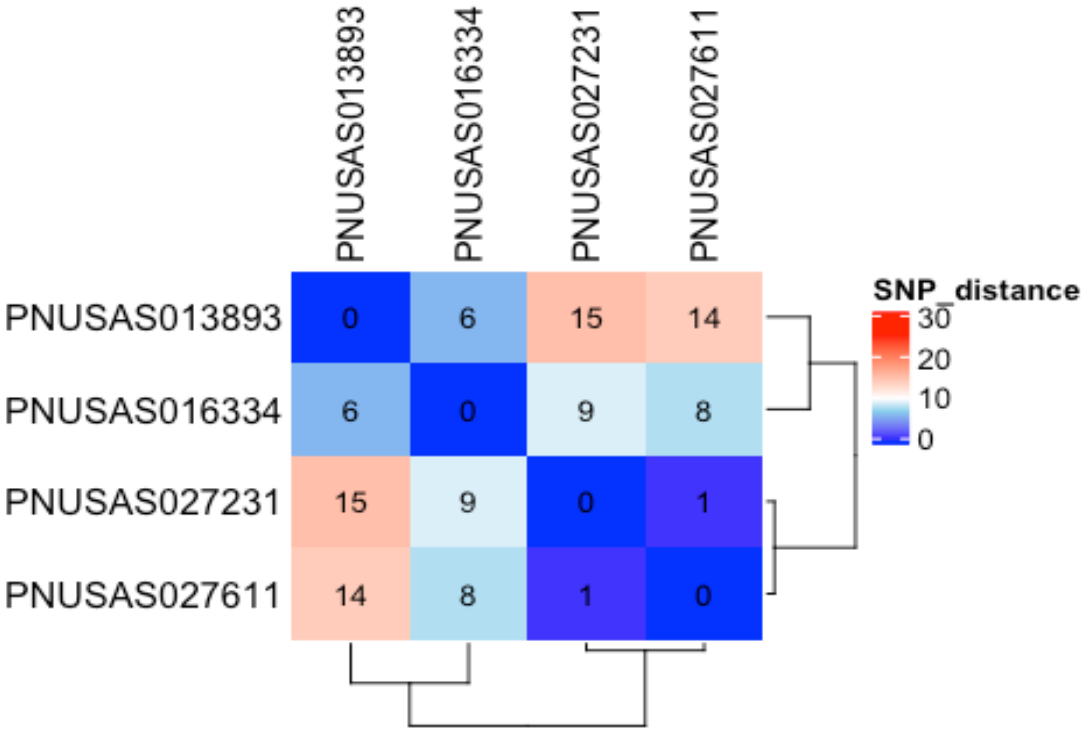

S6.3. SNP-distance Heatmap of HC5 576

Supplementary Figure S7:

Euclidian distance heatmap for 8 major HC5 clusters. The Euclidean distance between the centroids of each pair of zip code areas involved in HC5 clusters were calculated, and the distance matrix were illustrated in the heatmaps.

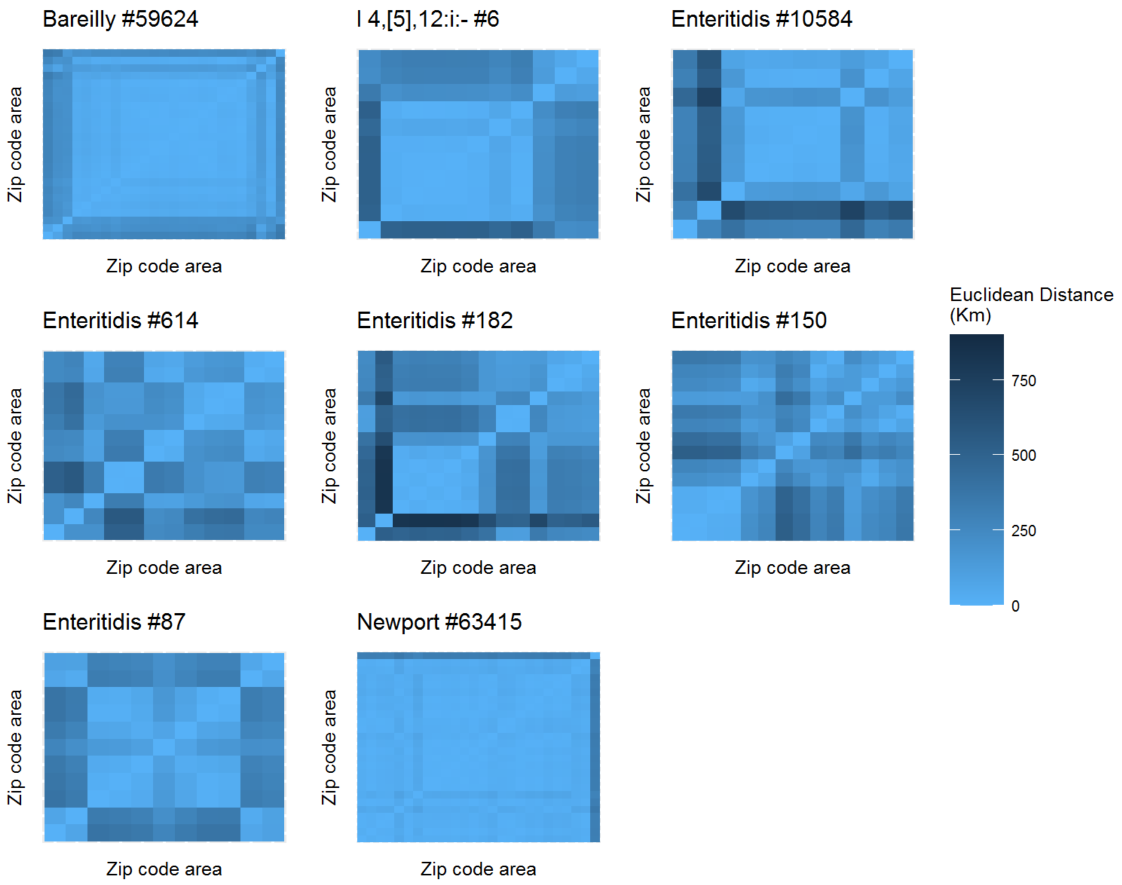

S7.1. Heatmap for distance matrix of zip code areas where major HC5 clusters occurred.

Supplementary Figure S8:

Clinical and non-clinical 1862 isolates collected from Florida during 2017-18, color codes by S8.1: Source and S8.2: Serotype

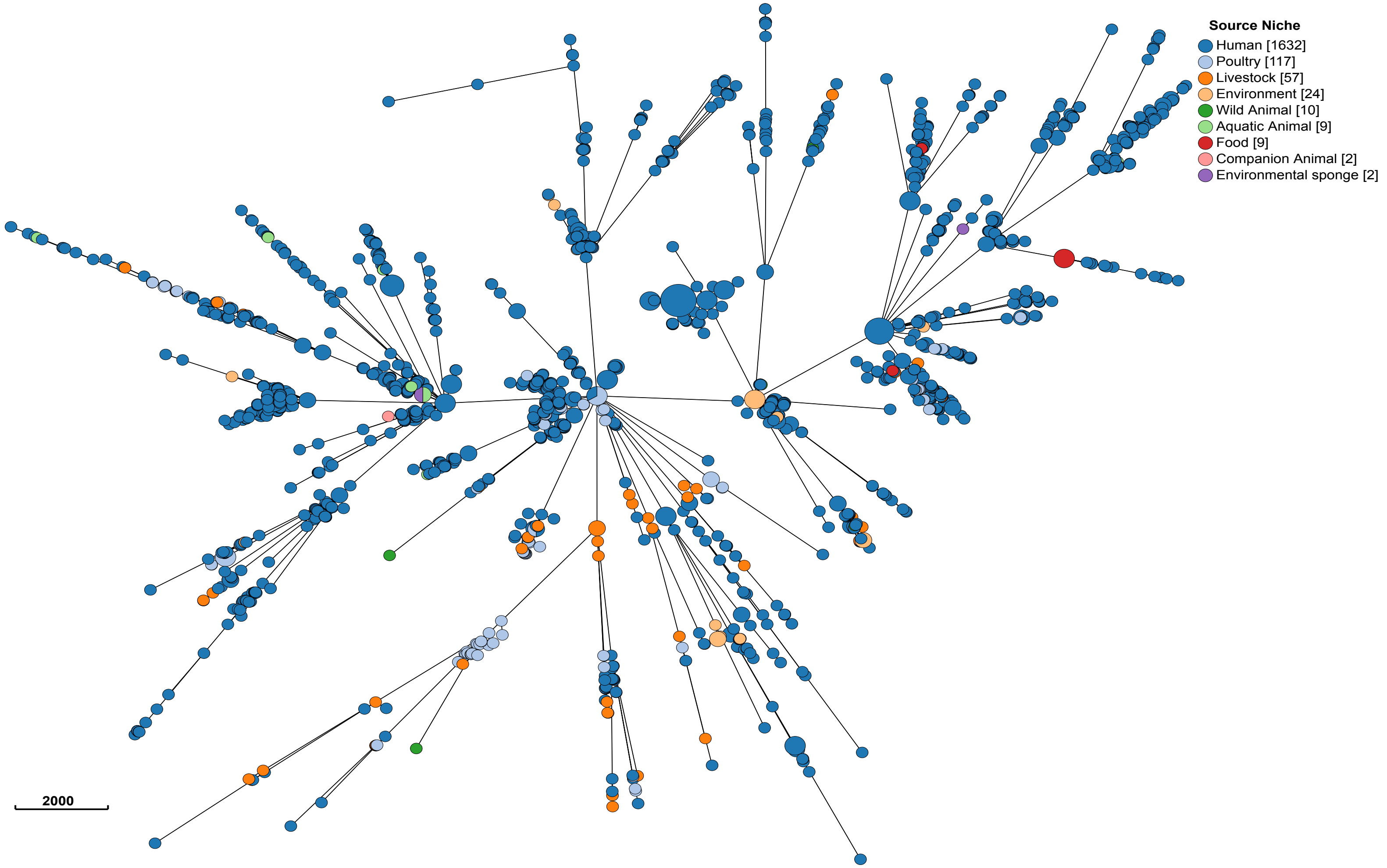

S8.1. Minimum Spanning tree of 1632 clinical and 230 non-clinical isolates in Florida 2017-18, nodes are color coded by Source and legend shows the corresponding number of isolates in each group.

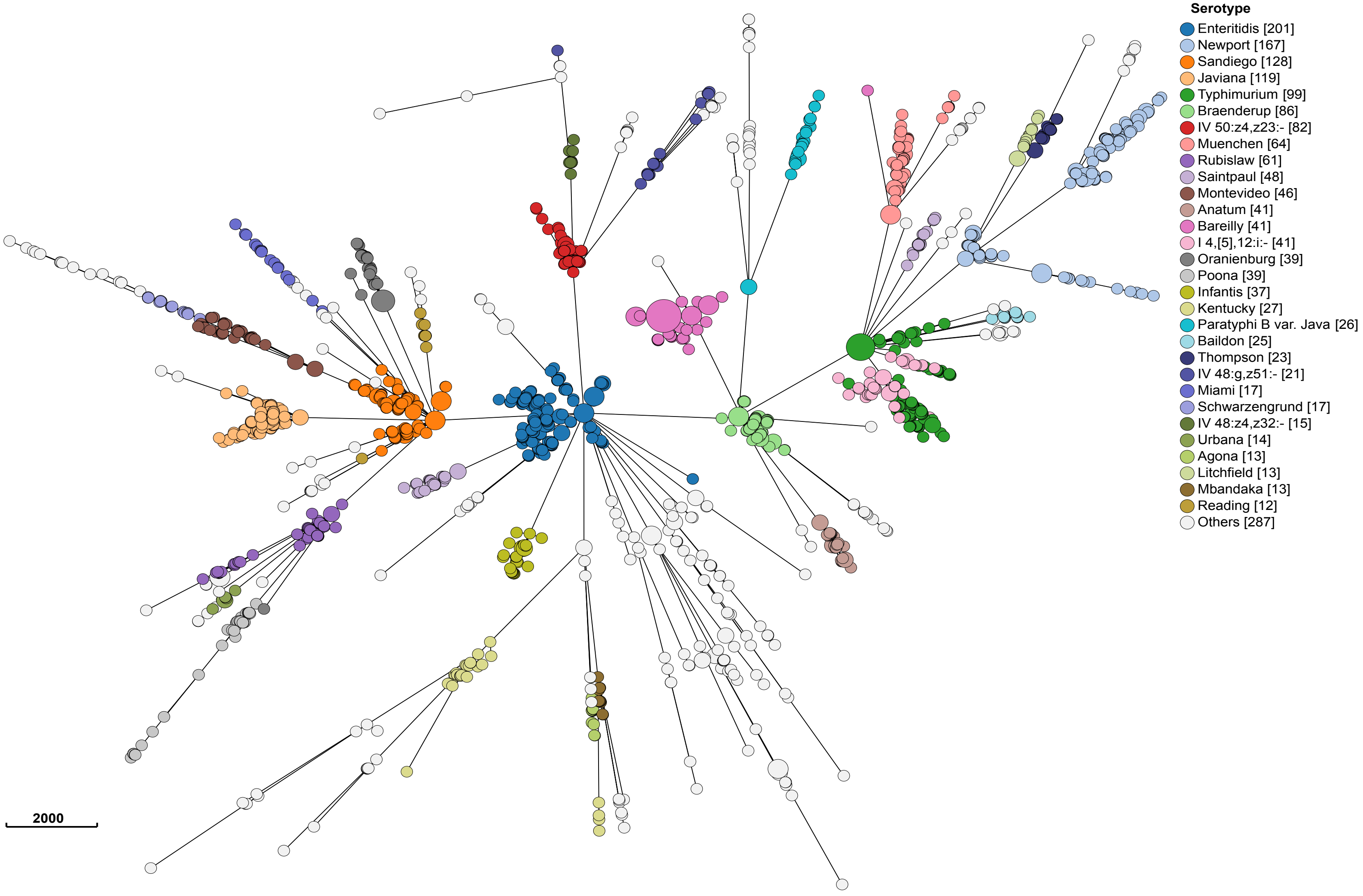

S8.2. Minimum Spanning tree of 1632 clinical and 230 non-clinical isolates in Florida 2017-18, nodes are color coded by Serotype and legend shows the corresponding number of isolates in each group

Supplementary Figure S9:

Heatmaps of **clinical** and **non-clinical** isolates of **S. Enteritidis** across Florida, drawn using pairwise SNP distance for HC5 clusters (>2 isolates) are shown in S9.1-4:

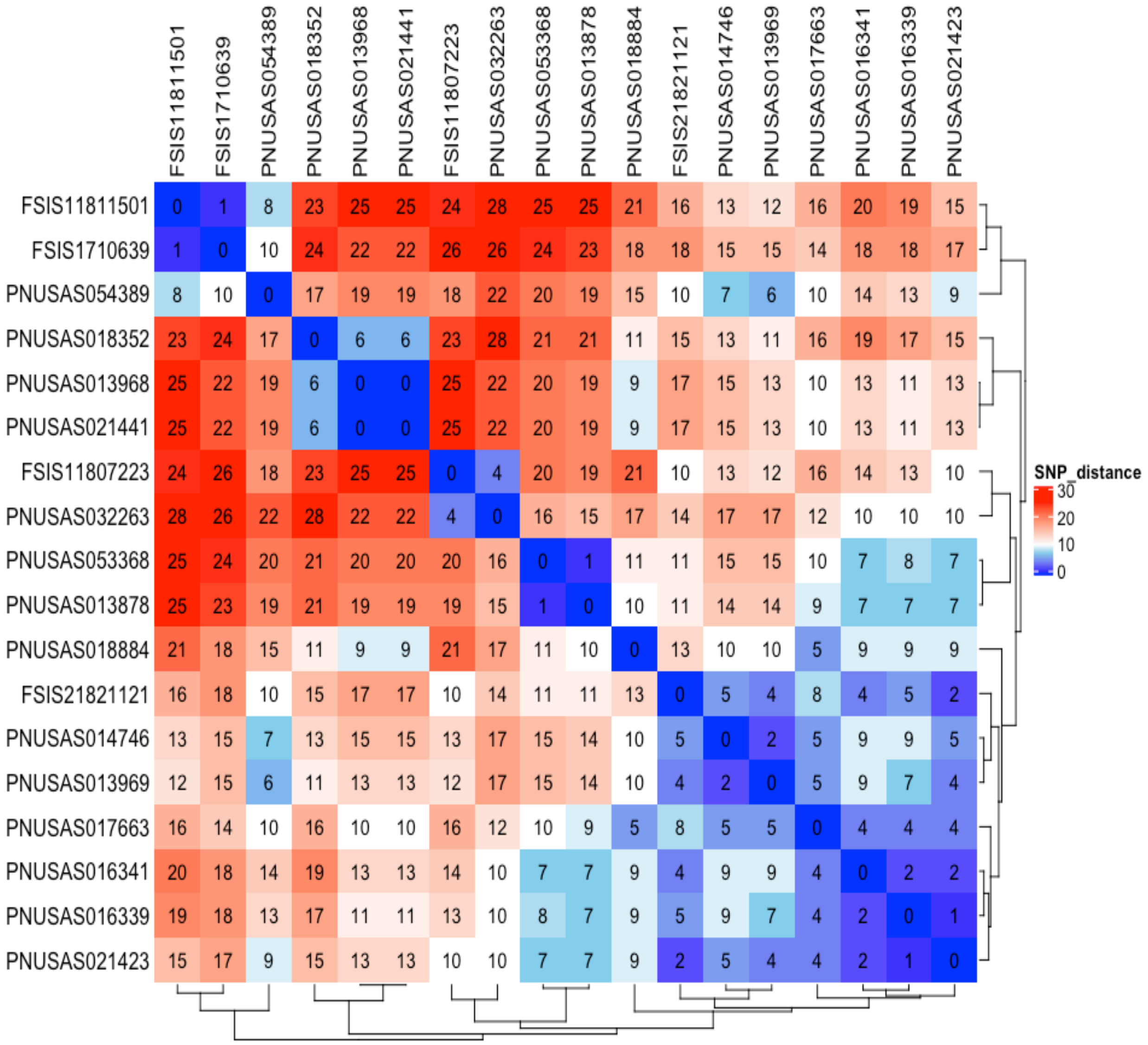

S9.1. SNP-distance Heatmap of HC5 150

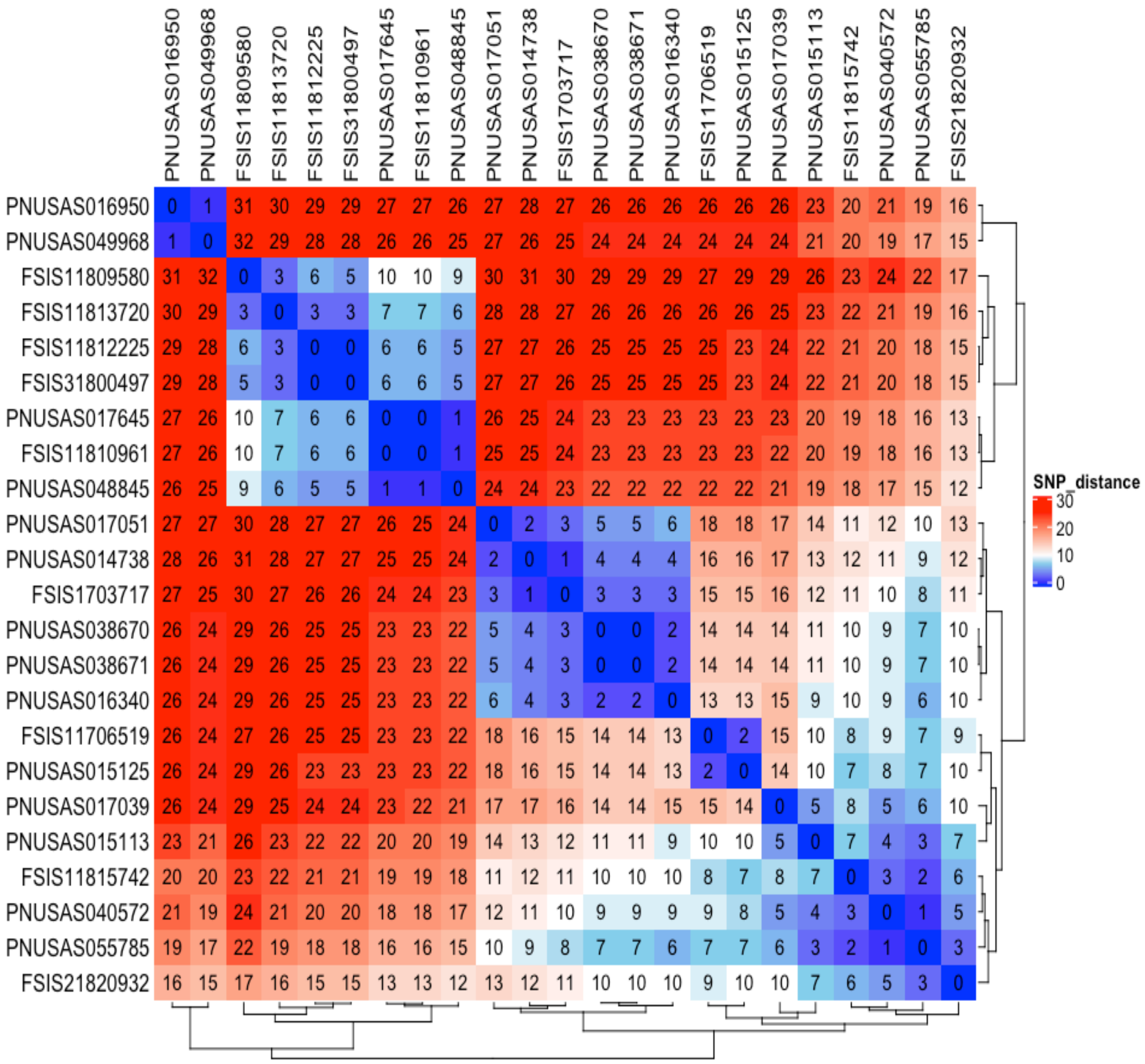

S9.2. SNP-distance Heatmap of HC5 182

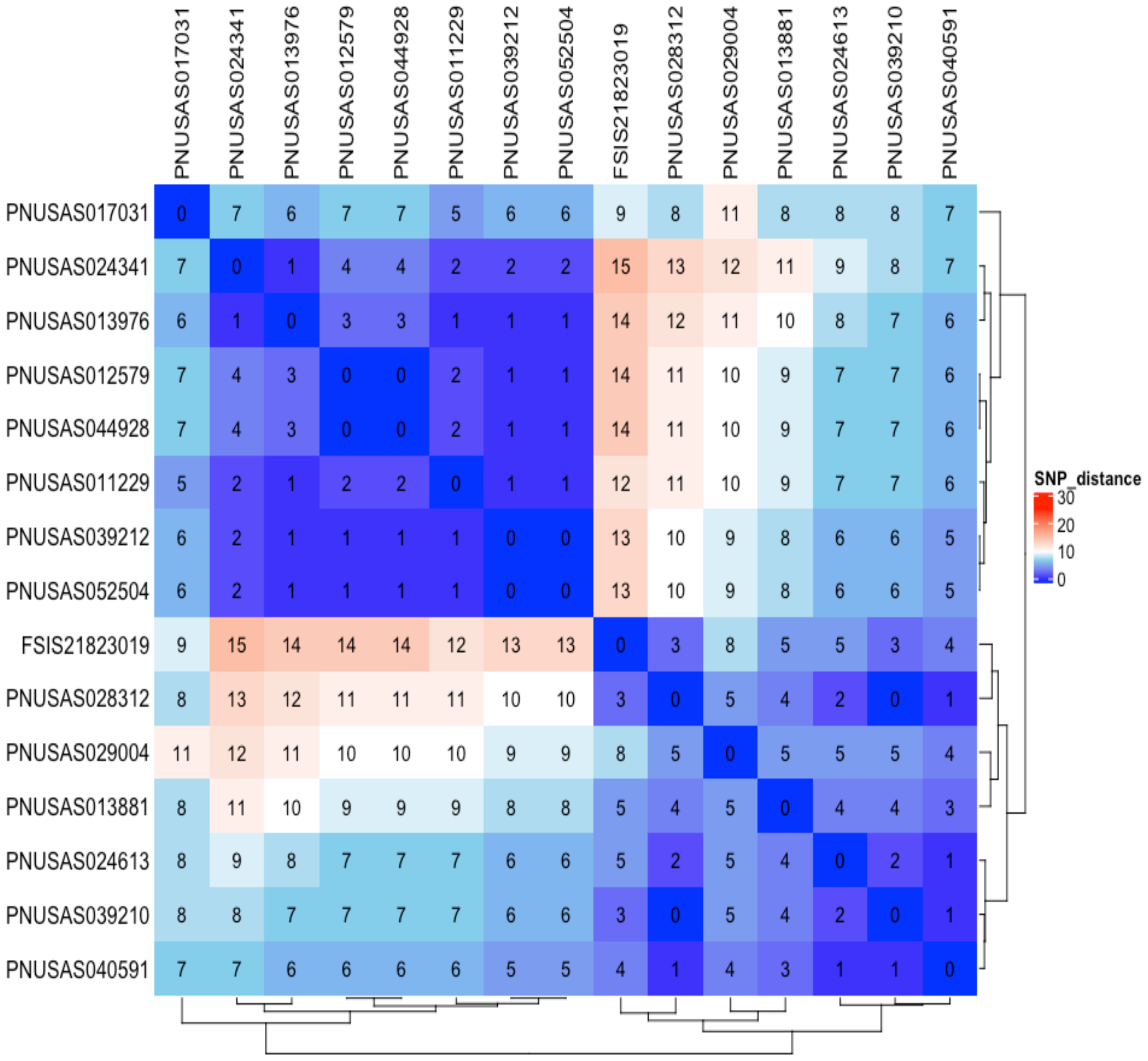

S9.3. SNP-distance Heatmap of HC5 614

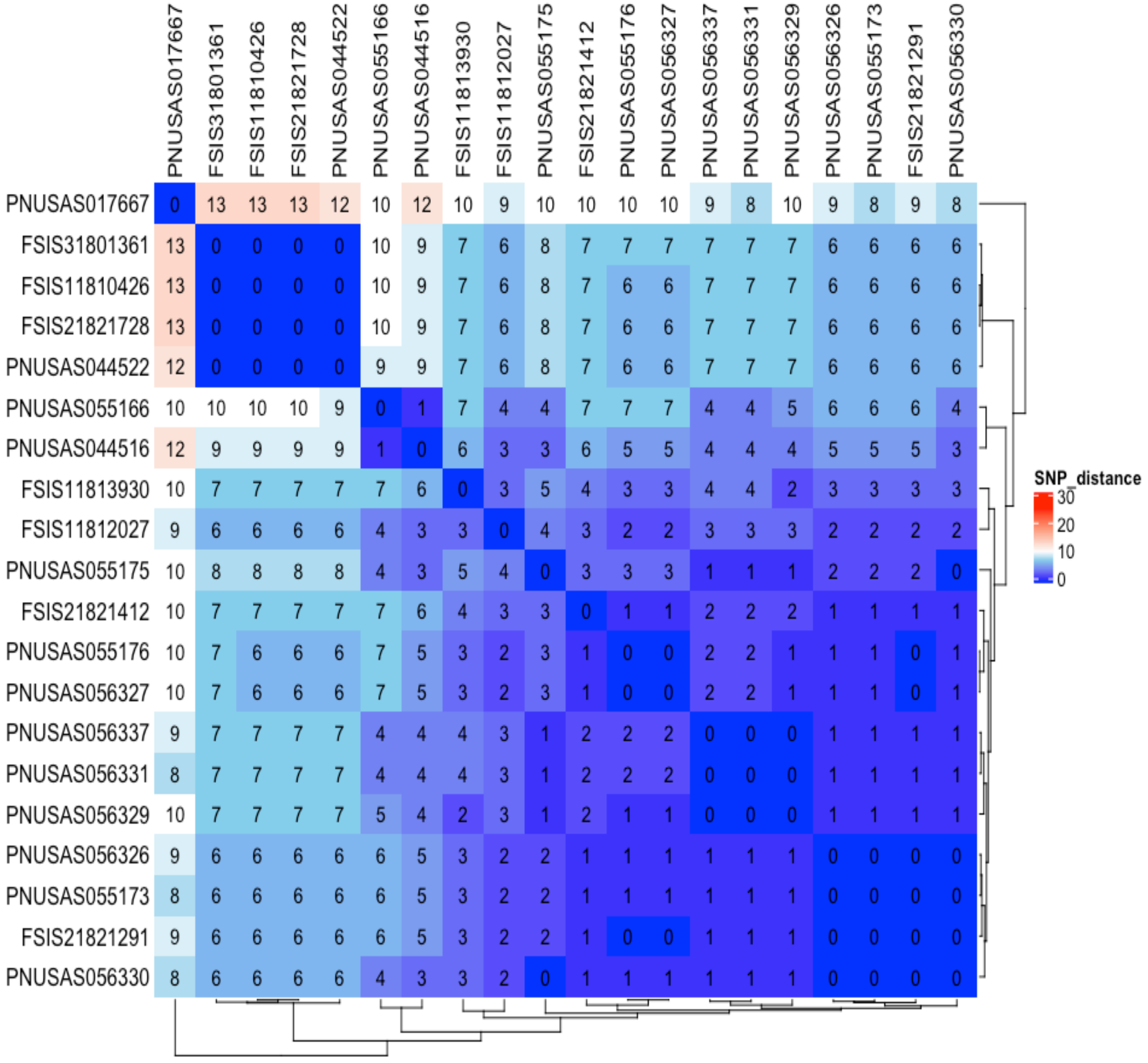

S9.4. SNP-distance Heatmap of HC5 10584

Supplementary Figure S10:

SNP-based Phylogenetic Trees of **clinical** and **non-clinical** isolates of ***S.* Enteritidis** across USA for 7 common HC5 clusters, drawn using pairwise SNP distance for HC5 clusters (>2 clinical isolates) are shown in S10.1-7:

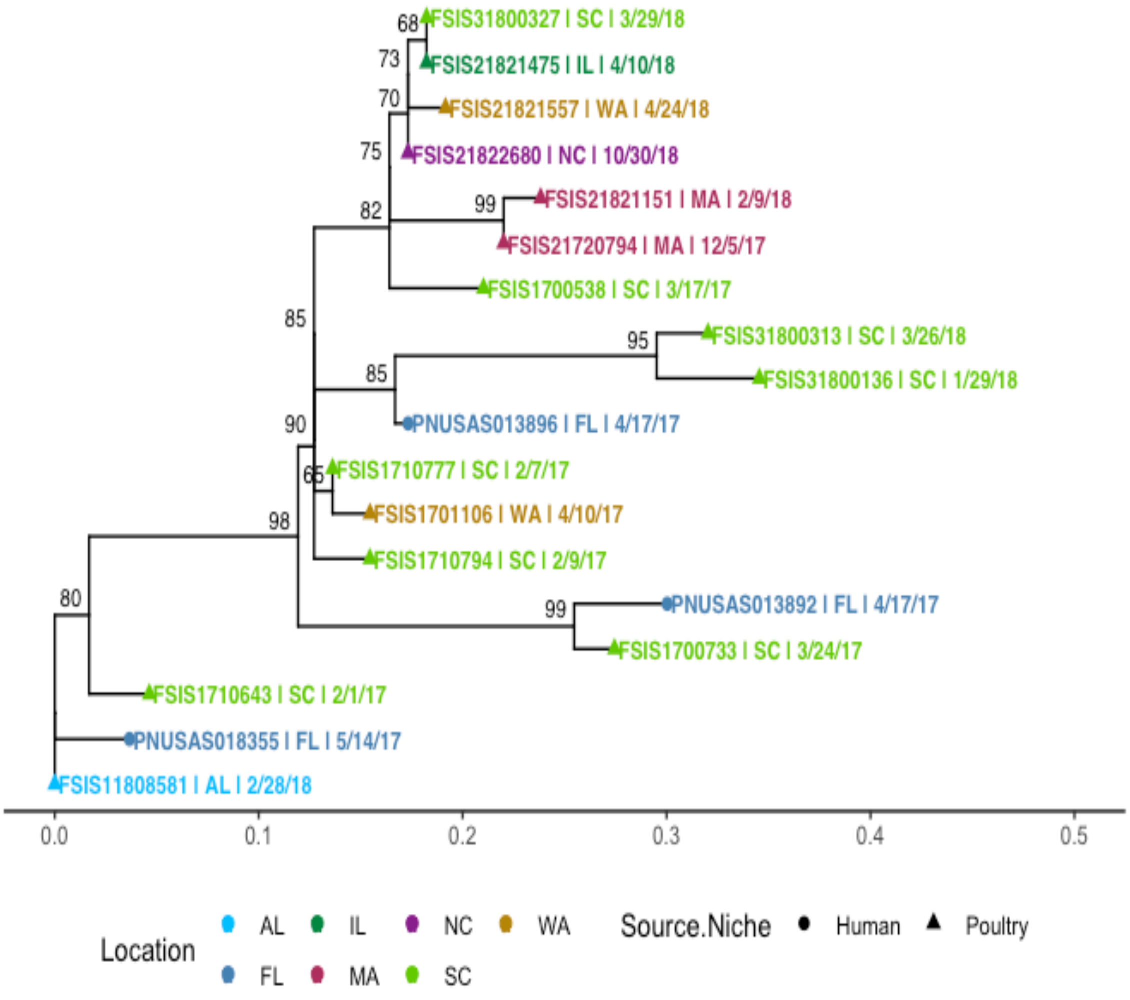

S10.1. SNP-based Phylogenetic Tree of HC5 85

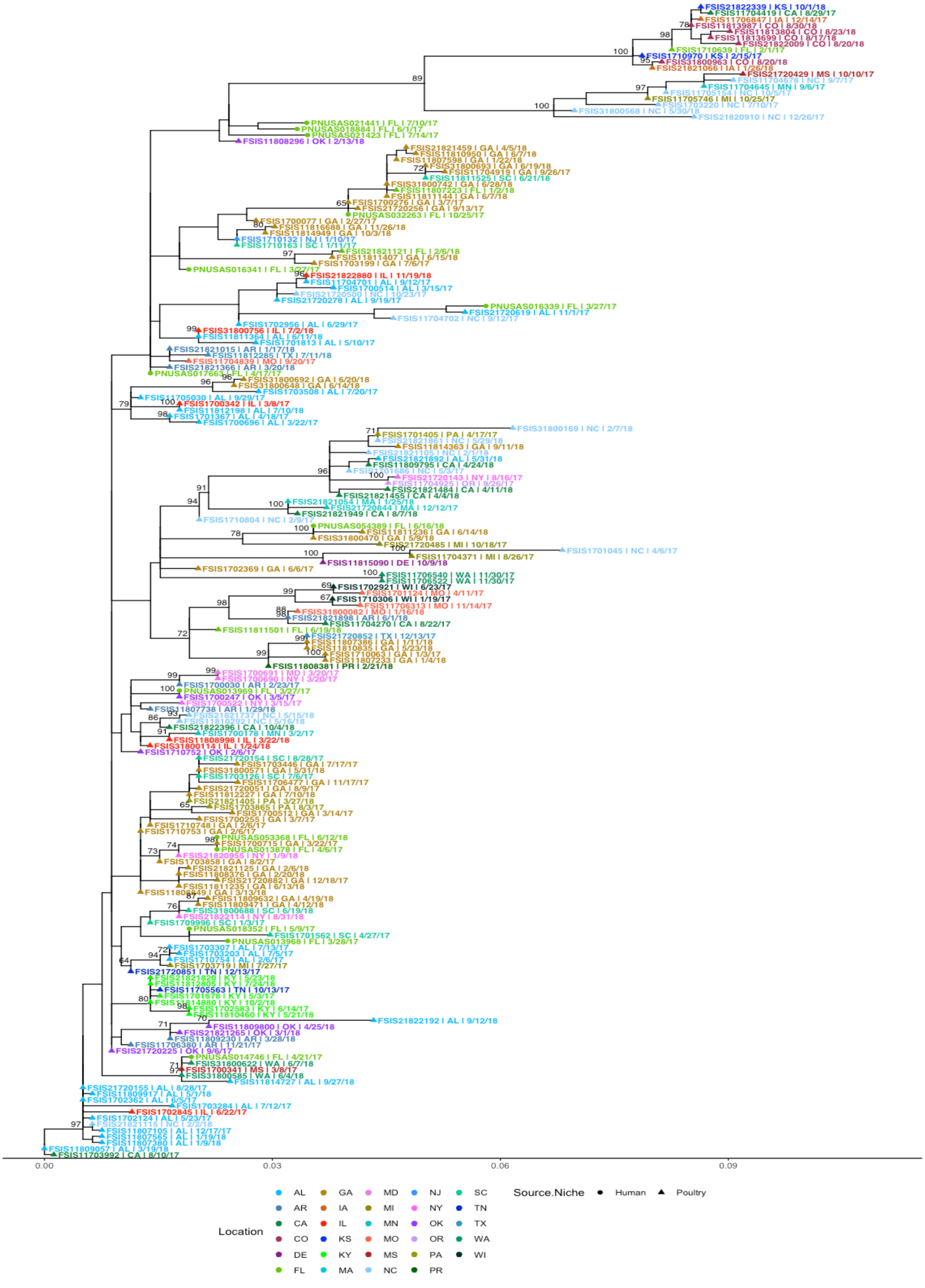

S10.2.SNP-based Phylogenetic Tree of HC5 150(*High resolution and can be zoomed in for details**)

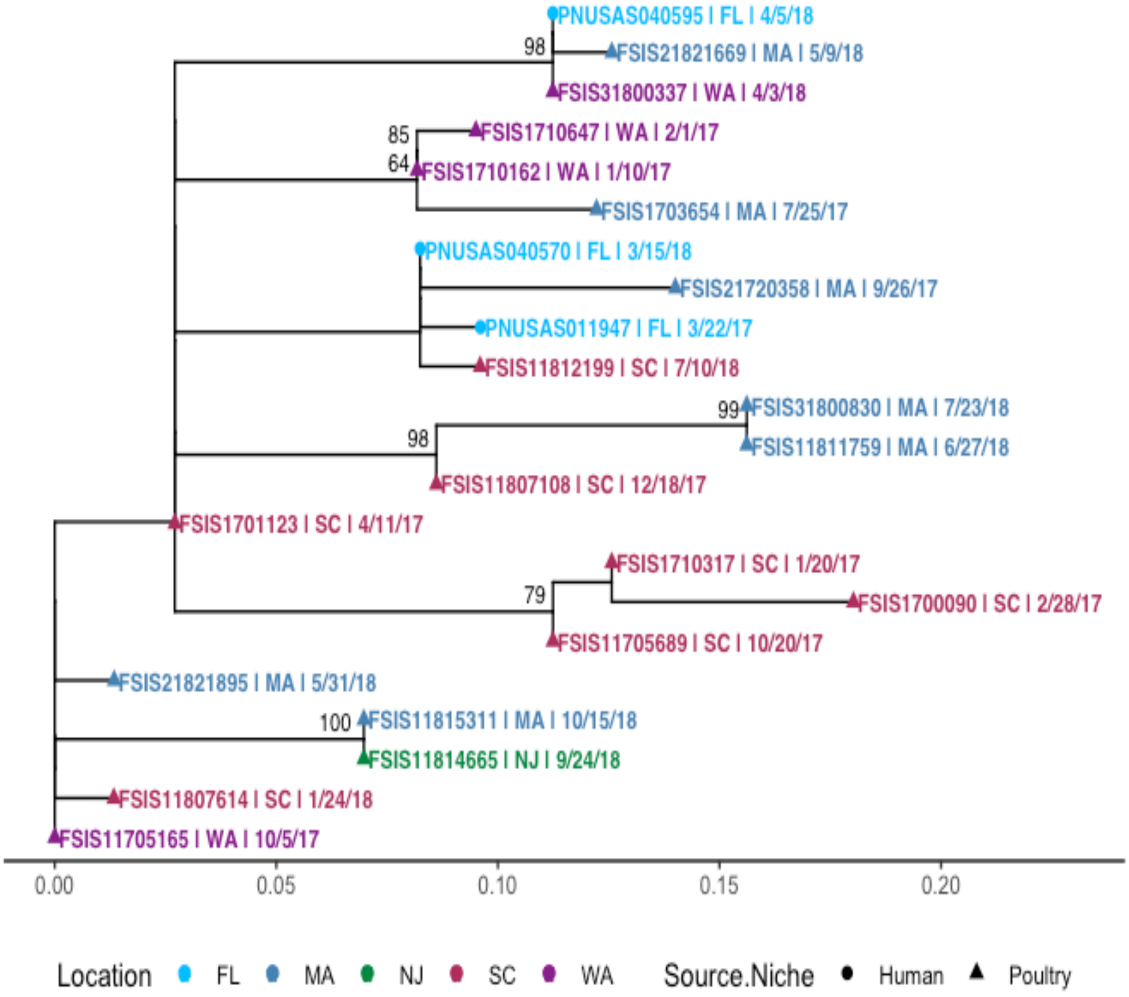

S10.3.SNP-based Phylogenetic Tree of HC5 165

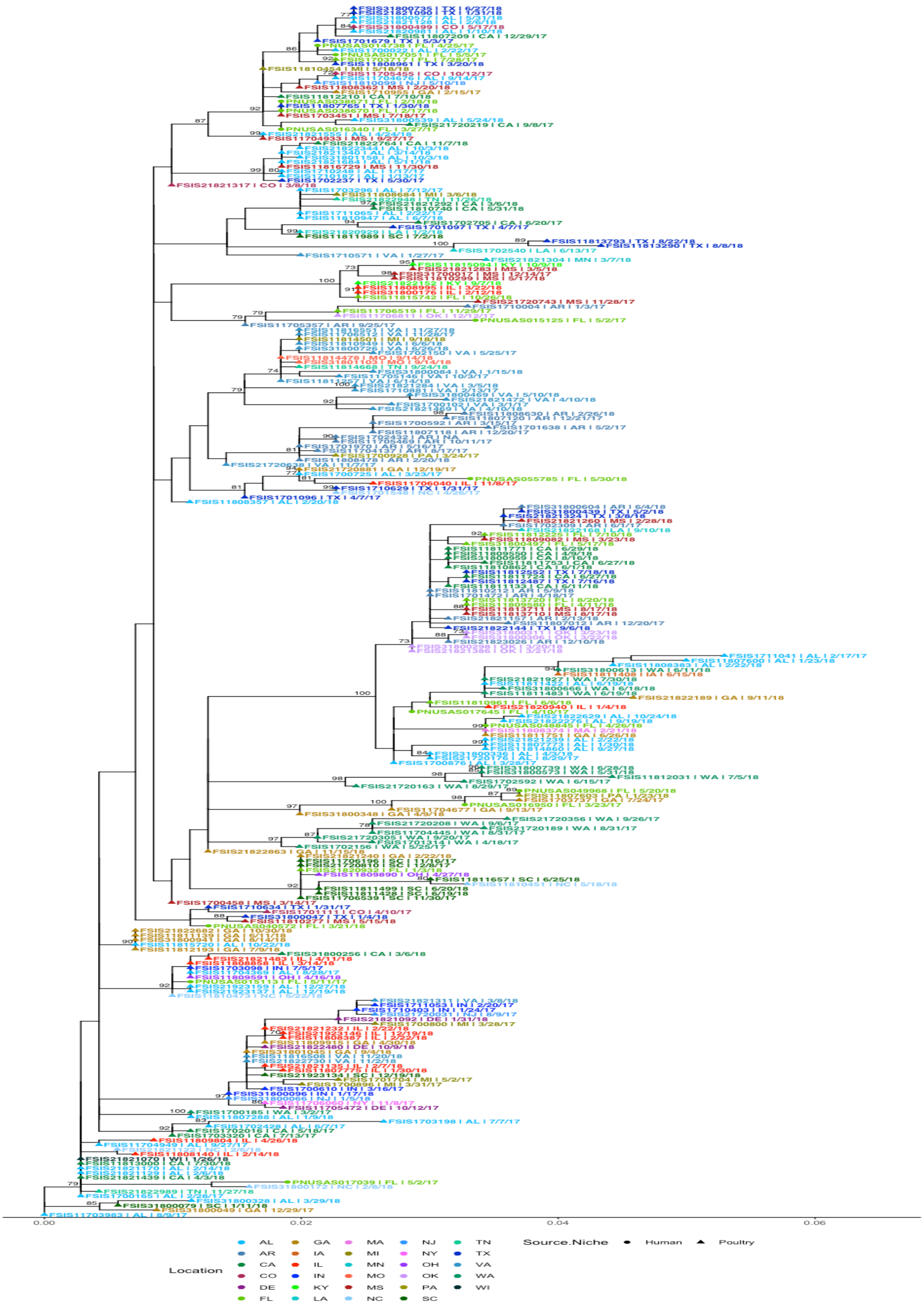

S10.4.SNP-based Phylogenetic Tree of HC5 182 (High resolution and can be zoomed in for details)*

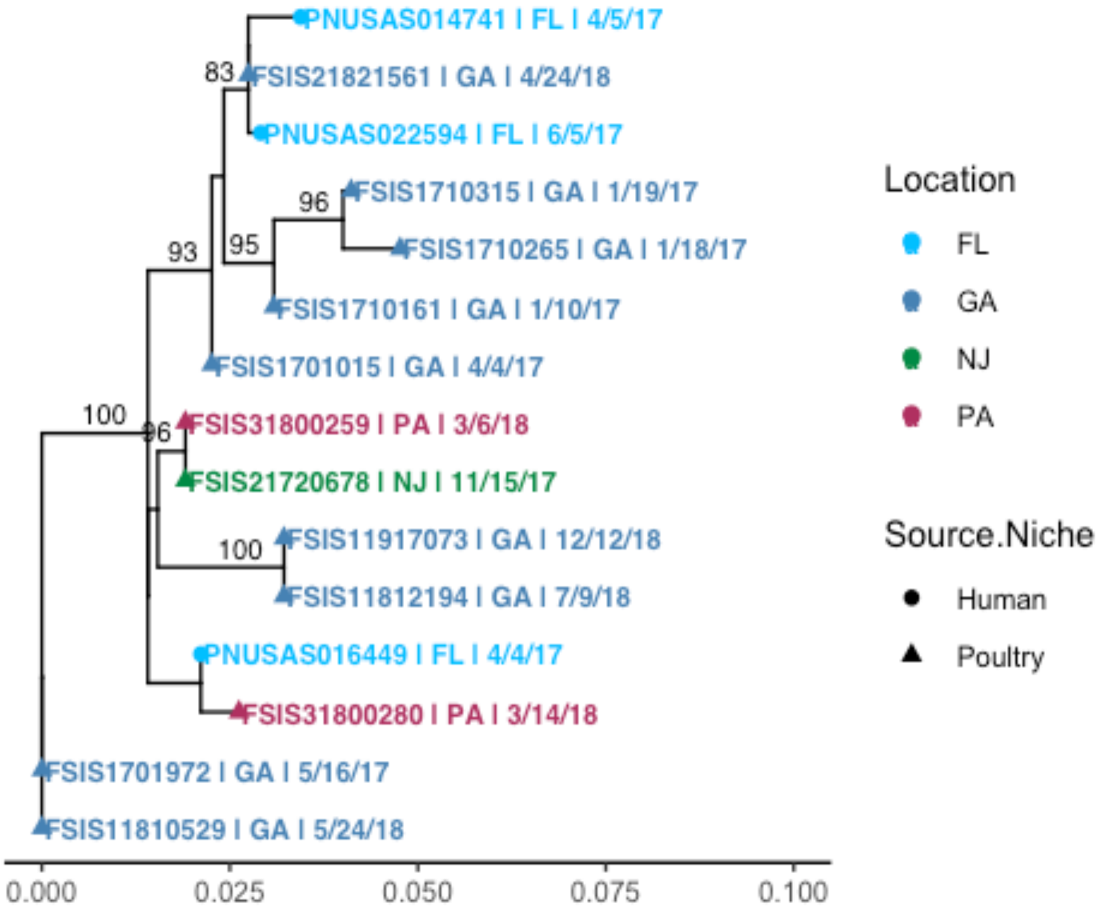

S10.5.SNP-based Phylogenetic Tree of HC5 9391

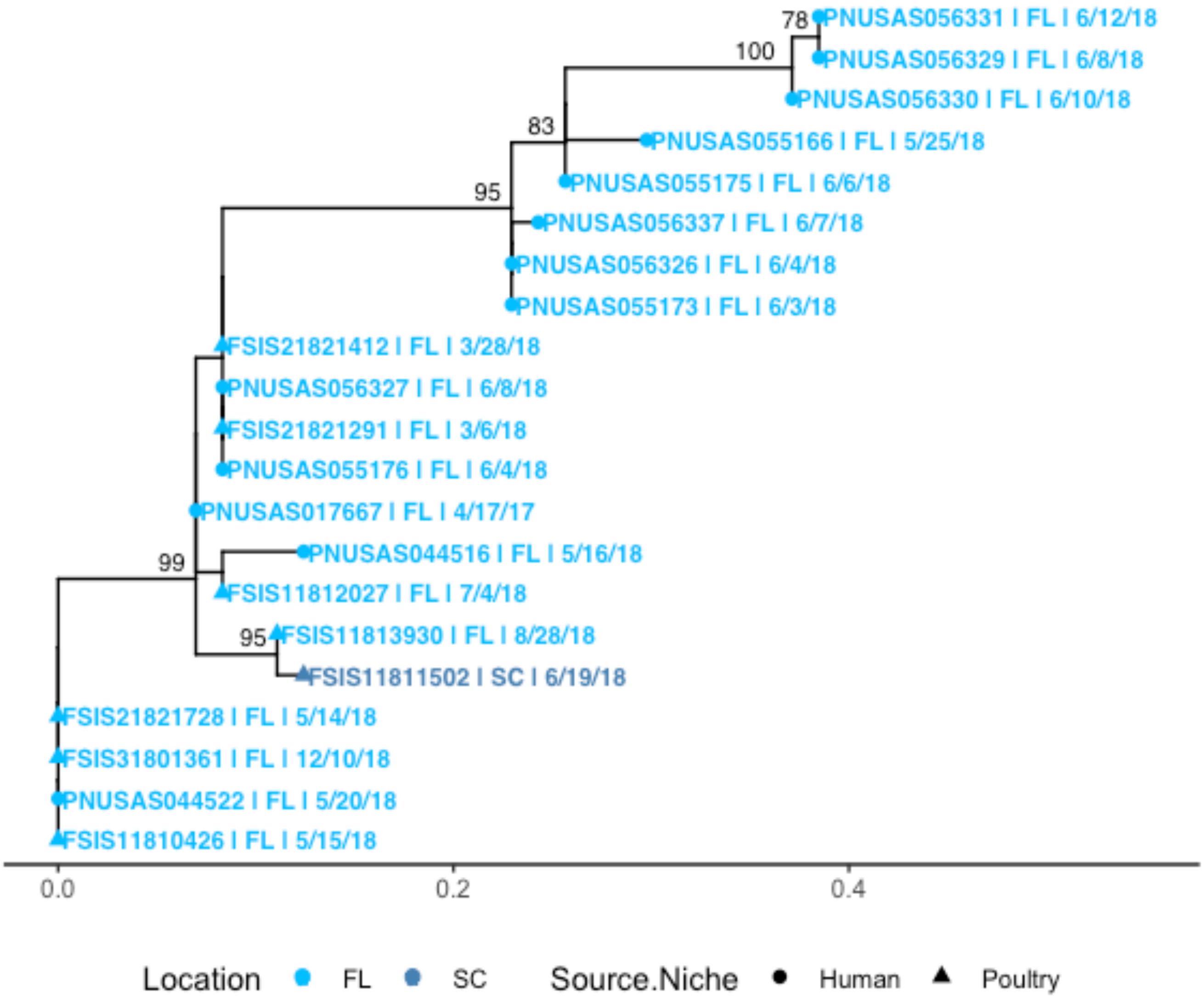

S10.6.SNP-based Phylogenetic Tree of HC5 10584

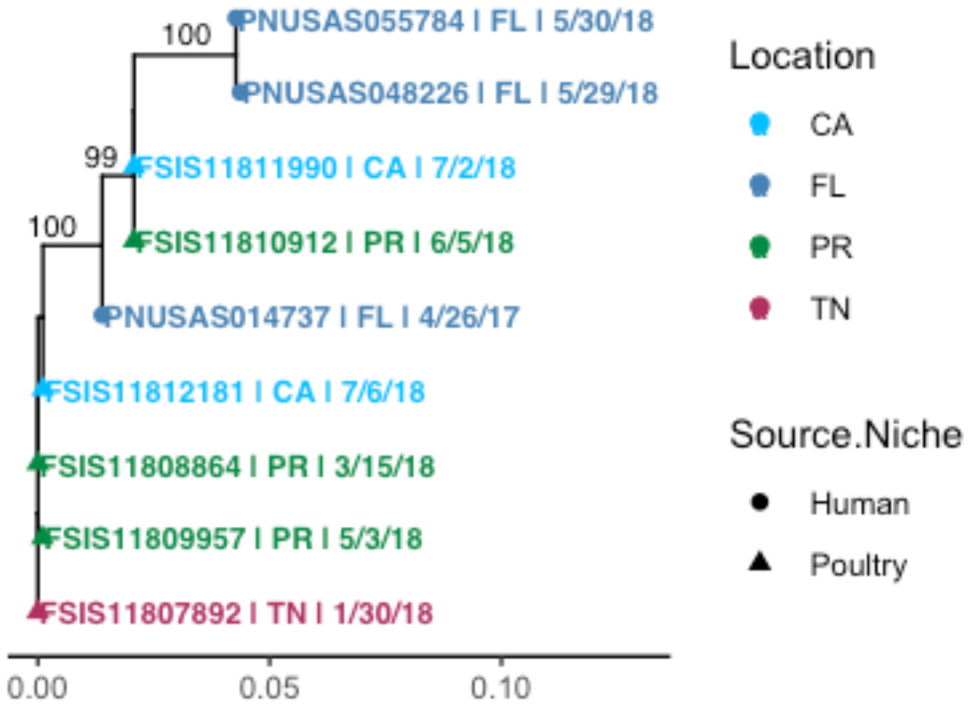

S10.7.SNP-based Phylogenetic Tree of HC5 62607

Supplementary Figure S11:

Heatmaps of **clinical** and **non-clinical** isolates of ***S.* Enteritidis** across USA for 8 common HC5 clusters, drawn using pairwise SNP distance for HC5 clusters (>2 clinical isolates) are shown in S11.1-8:

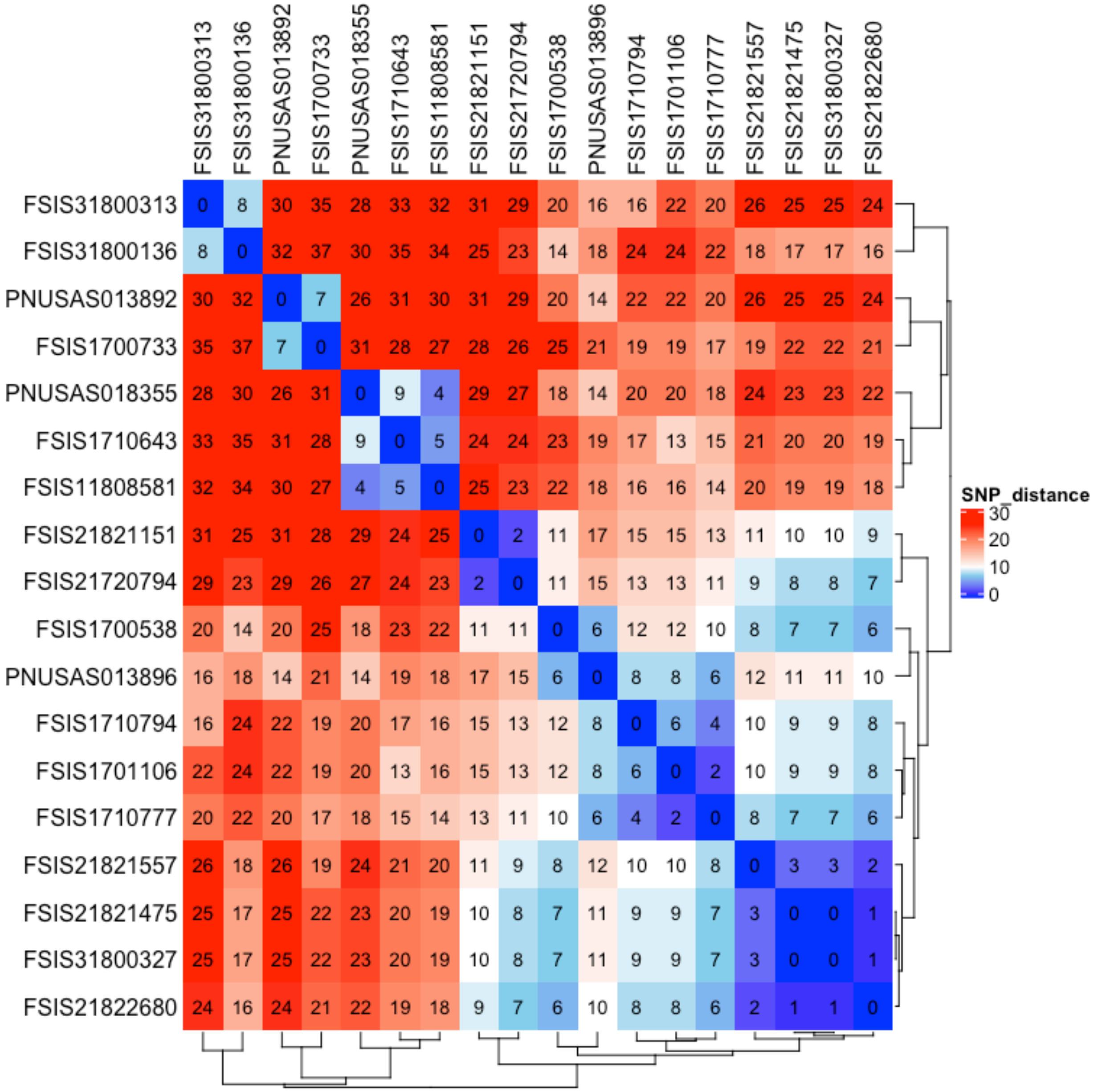

S11.1. SNP-distance Heatmap of HC5 85

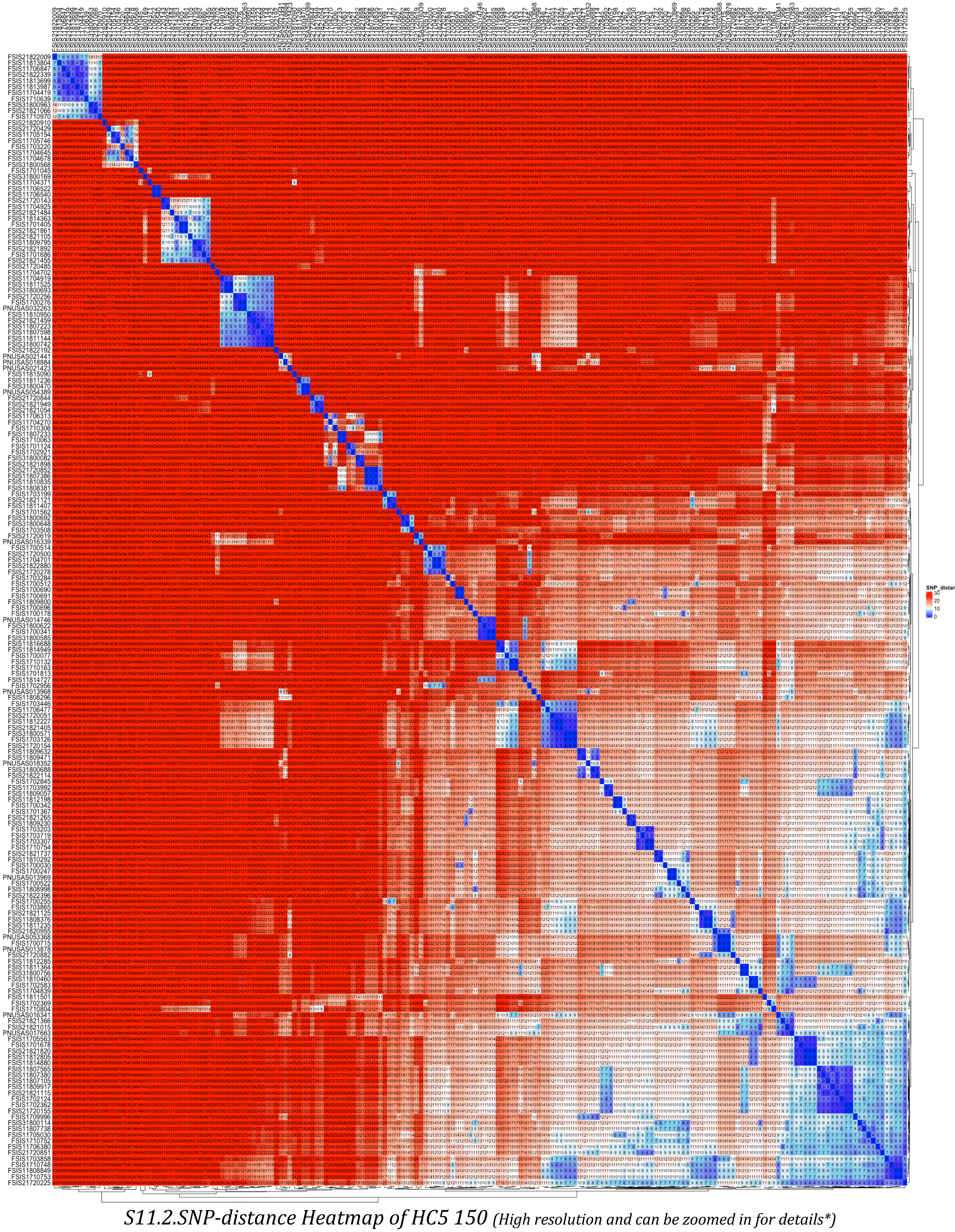

S11.2.SNP-distance Heatmap of HC5 150 *(High resolution and can be zoomed in for details*)*

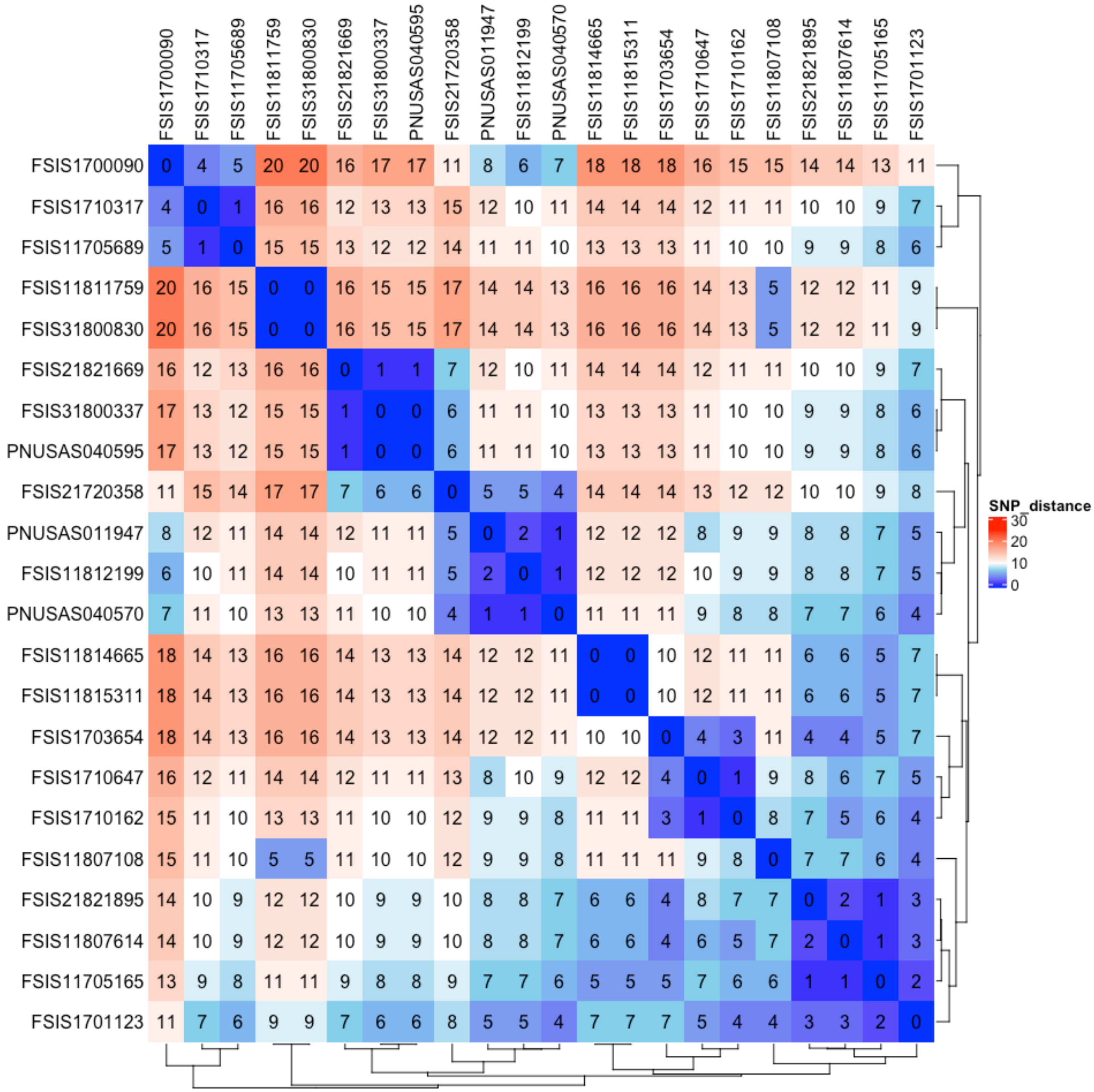

S11.3.SNP-distance Heatmap of HC5 165

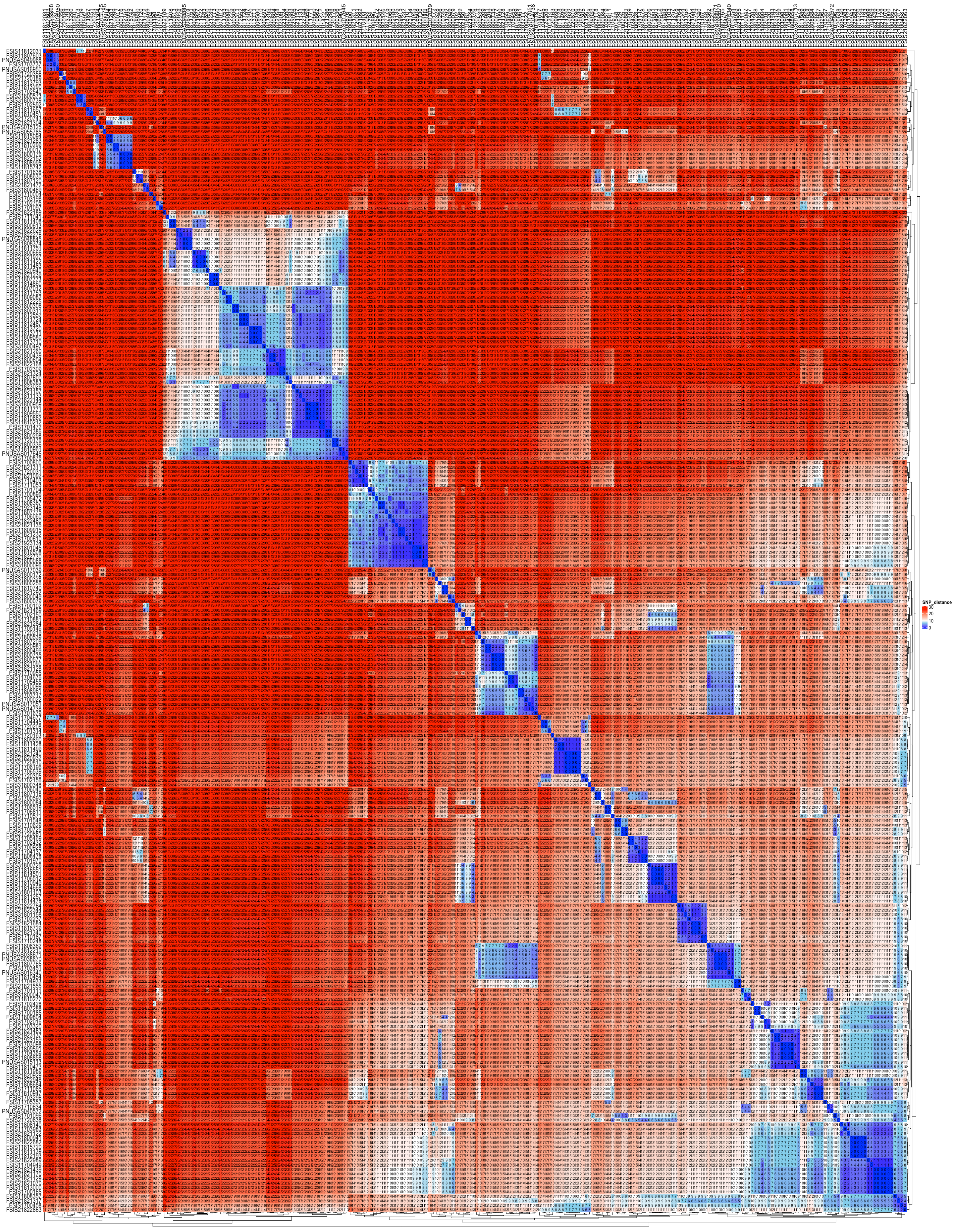

S11.4.SNP-distance Heatmap of HC5 182 *(High resolution and can be zoomed in for details*)*

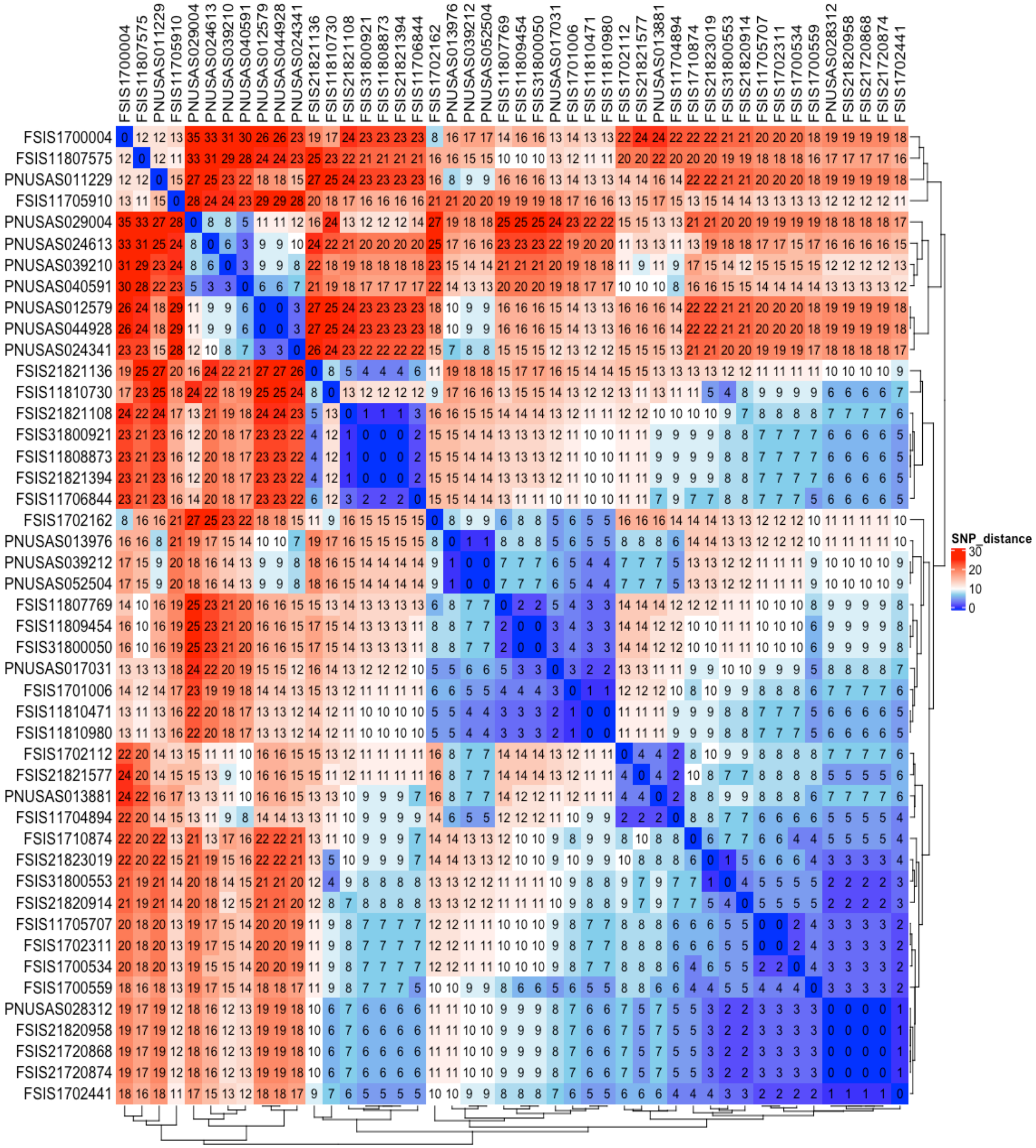

S11.5.SNP-distance Heatmap of HC5 614

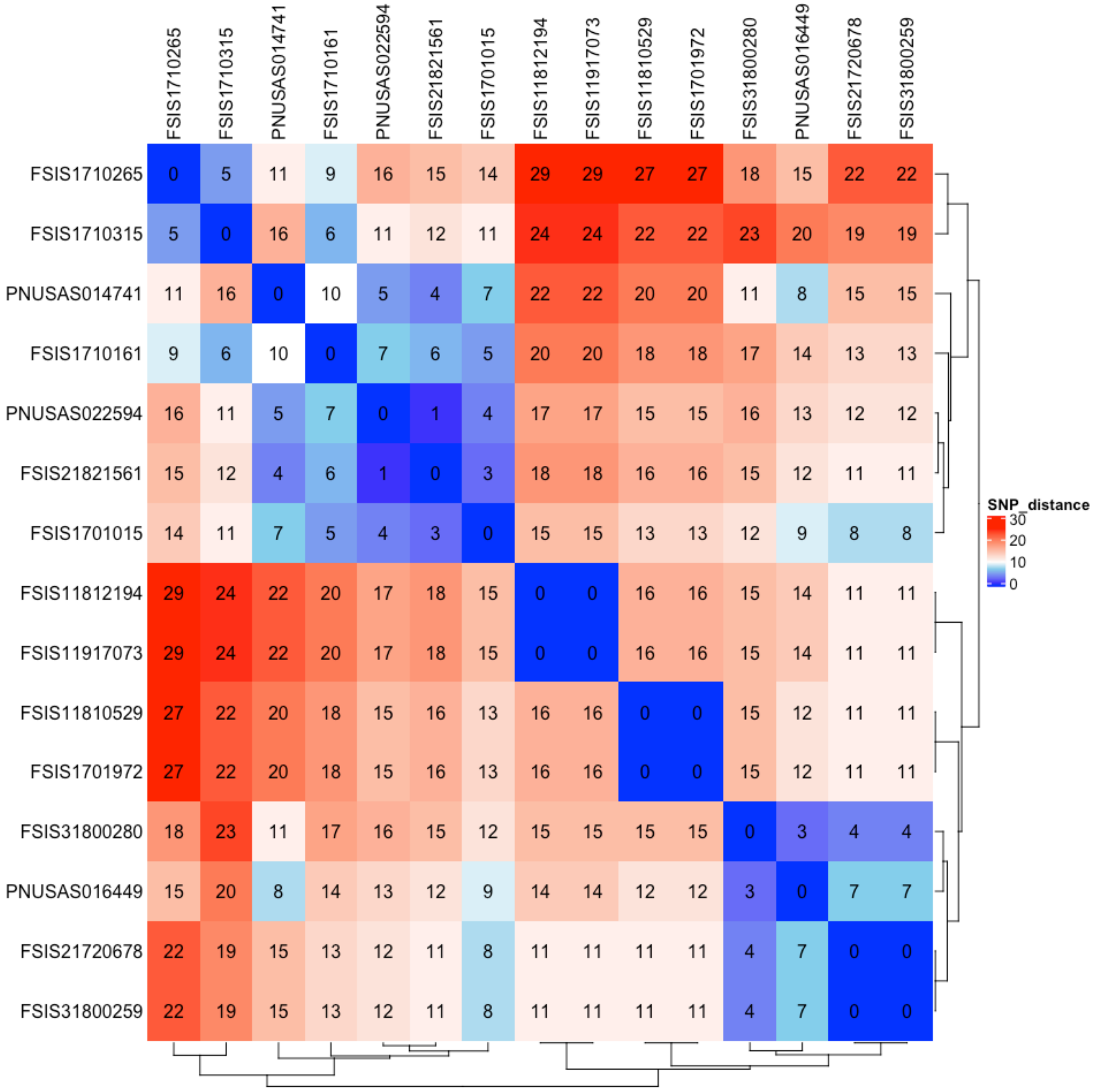

S11.6.SNP-distance Heatmap of HC5 9391

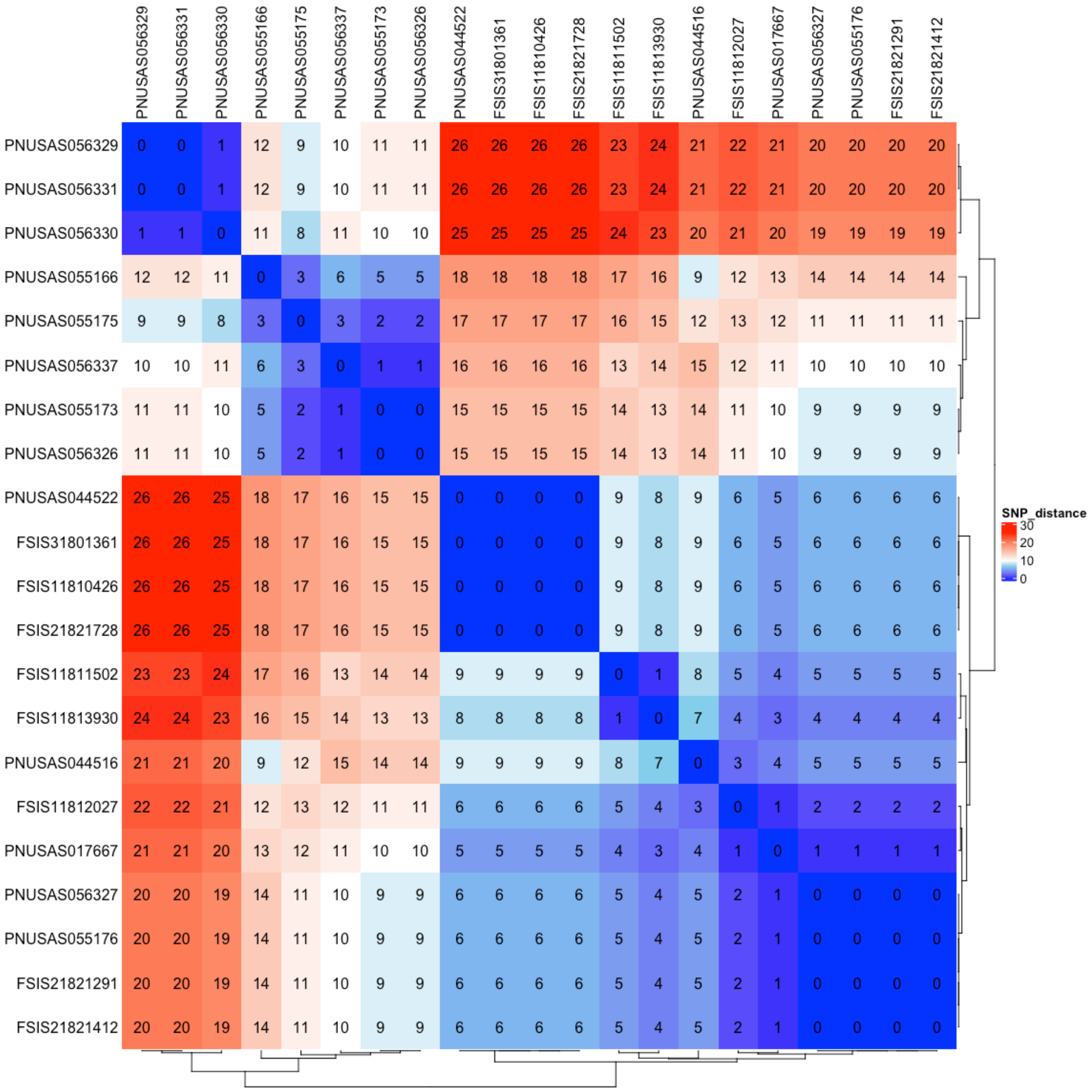

S11.7.SNP-distance Heatmap of HC5 10584

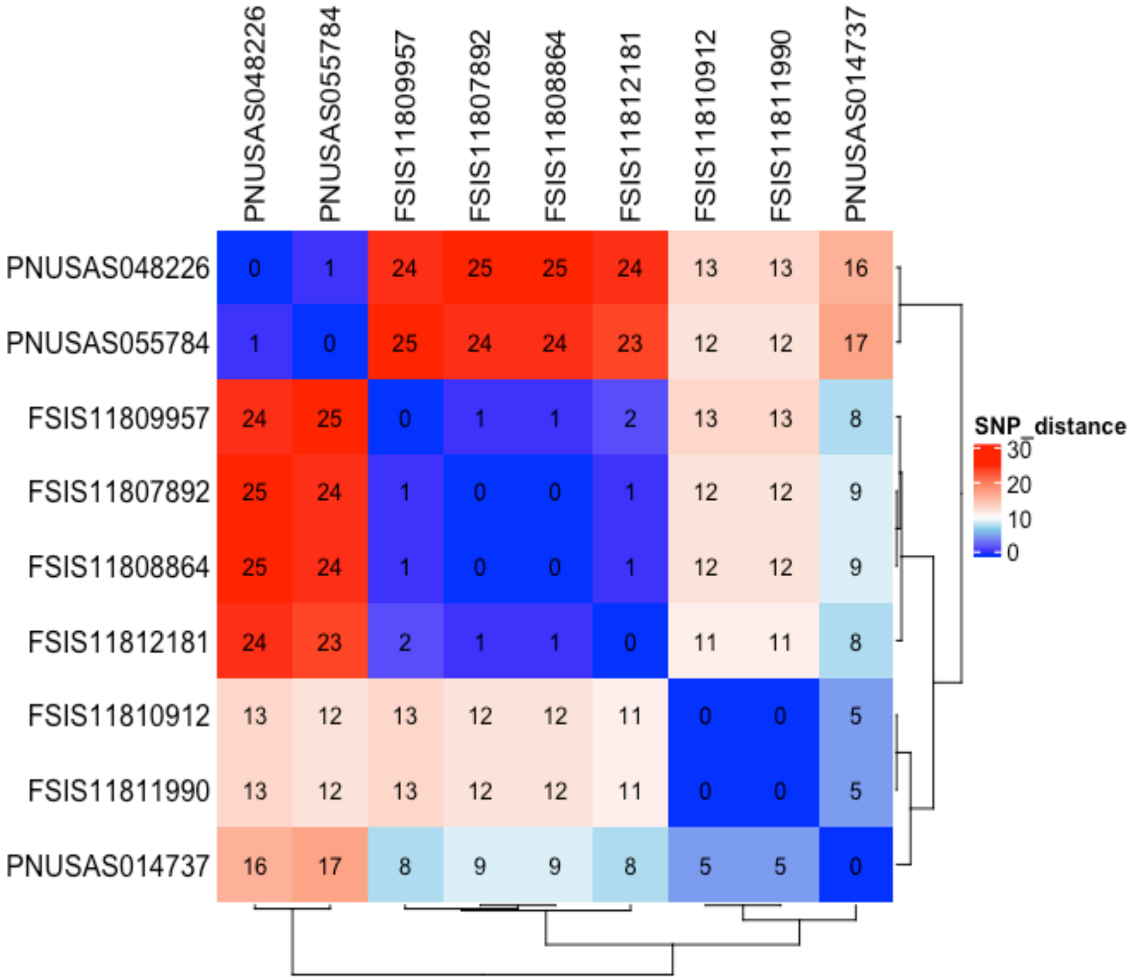

S11.8.SNP-distance Heatmap of HC5 62607
