# Supplementary material for: Molecular Epidemiology of Salmonellosis in Florida, USA, 2017–2018
Source: Front Med (Lausanne). 2021 Apr 22;8:656827. doi: 10.3389/fmed.2021.656827 (PMC8100233; doi:10.3389/fmed.2021.656827)
Supplement: Supplementary file 4 [file Data_Sheet_4.docx]

**Data Dictionary**

**Supplementary Datasheet 2**

| **Column Name** | | **Definition** |
| --- | --- | --- |
| Strain |  | Isolate name, submitted by sequencing lab |
| NCBI_ACCESSION | | Identifying number for NCBI to link with the RAW genome sequence stored in NCBI database |
| SISTR1_Serotype | | Predicted serotype for the Salmonella isolate using WGS data using SISTR ver. 1 tool |
| PFGE XbaI pattern | | PFGE pattern ID |
| ST_7MLST |  | Sequence Type based on legacy MLST type |
| eBG_7MLST |  | e Burts group based on Legacy MLST type |
| HC5_cgMLST | | Hierarchical Cluster (allelic distance <=5 ) profile Number, based on core genome MLST type |
| HC2_cgMLST | | Hierarchical Cluster (allelic distance <=2 ) profile Number, based on core genome MLST type |
| IsolatDate |  | Date the Salmonella isolate was collected |
| Age |  | Age of case, isolate was collected from at the time of illness |
| Gender |  | Gender of case, isolate was collected from |
| Ethnicity |  | Ethnicity of case, isolate was collected from |
| Source Niche | | Source of isolate (human/animal/environmental) |
| Source Site |  | Type of source isolate identified in (Blood, stool, urine, wound) |
| COUNTYNAME | | Name of County (converted form reported zip code) where patient from whome sample have been islated, resides |

**Supplementary Datasheet 3**

| **Column Name** | | **Definition** |  |
| --- | --- | --- | --- |
| Strain |  | Isolate name, submitted by sequencing lab |  |
| NCBI_ACCESSION | | Identifying number for NCBI to link with the RAW genome sequence stored in NCBI database |  |
| SISTR1_Serotype | | Predicted serotype for the Salmonella isolate using WGS data using SISTR ver. 1 tool |  |
| HC5_cgMLST | | Hierarchical Cluster (allelic distance <=5 ) profile Number, based on core genome MLST type |  |
| HC2_cgMLST | | Hierarchical Cluster (allelic distance <=2 ) profile Number, based on core genome MLST type |  |
| Source Niche | | Source of isolate (human/animal/environmental) |  |
| IsolatDate | | Date the Salmonella isolate was collected |  |
| Location |  | USA State of Sample Collection |  |
| Collected By | | Name of agency who collected the sample from collection site |  |
| Virulence_genotypes | | Antimicrobial genotypes predicted by NCBI AMRFinder |  |
| AMR_genotypes | | Antimicrobial genotypes predicted by NCBI AMRFinder |  |
